# FONDUE: Robust resolution-invariant denoising of MR images using Nested UNets

**DOI:** 10.1162/imag_a_00374

**Published:** 2024-11-21

**Authors:** Walter Adame-Gonzalez, Aliza Brzezinski-Rittner, Yashar Zeighami, M. Mallar Chakravarty, Reza Farivar, Mahsa Dadar

**Affiliations:** Integrated Program in Neuroscience, McGill University, Montreal, Quebec, Canada; Cerebral Imaging Centre – Douglas Research University Hospital, Verdun, Quebec, Canada; Department of Psychiatry, McGill University, Montreal, Quebec, Canada; Computational Brain Anatomy Laboratory, Cerebral Imaging Center, Douglas Mental Health University Institute, Montreal, Quebec, Canada; Department of Biological and Biomedical Engineering, McGill University, Montreal, Quebec, Canada; Department of Ophthalmology & Visual Sciences, McGill University, Montreal, Quebec, Canada; Research Institute of the McGill University Health Center, Montreal, Quebec, Canada

**Keywords:** denoising, magnetic resonance images, deep neural networks, UNet

## Abstract

Recent human magnetic resonance imaging (MRI) studies continually push the boundaries of spatial resolution as a means to enhance levels of neuroanatomical detail and increase the accuracy and sensitivity of derived brain morphometry measures. However, acquisitions required to achieve these resolutions have a higher noise floor, potentially impacting segmentation and morphometric analysis results. This study proposes a novel, fast, robust, and resolution-invariant deep learning method to denoise structural human brain MRIs. We explore denoising of T1-weighted (T1w) brain images from varying field strengths (1.5T to 7T), voxel sizes (1.2 mm to 250 µm), scanner vendors (Siemens, GE, and Phillips), and diseased and healthy participants from a wide age range (young adults to aging individuals). Our proposed Fast-Optimized Network for Denoising through residual Unified Ensembles (FONDUE) method demonstrated stable denoising capabilities across multiple resolutions with performance on par or superior to the state-of-the-art methods while being several orders of magnitude faster at low relative cost when using a dedicated Graphics Processing Unit (GPU). FONDUE achieved the best performance on at least one of the four denoising-performance metrics on all the test datasets used, showing its generalization capabilities and stability. Due to its high-quality performance, robustness, fast execution times, and relatively low-GPU memory requirements, as well as its open-source public availability, FONDUE can be widely used for structural MRI denoising, especially in large-cohort studies. We have made the FONDUE repository and all training and evaluation scripts as well as the trained weights available athttps://github.com/waadgo/FONDUE.

## 1 Introduction

Magnetic resonance images (MRIs) are affected by noise originating from various sources such as stochastic variation, physiological processes, eddy currents, magnetic field susceptibilities of adjacent tissues, and motion (Zhu et al., 2009). Recent large-cohort studies such as the Human Connectome Project (HCP) (Van Essen et al., 2012) have explored sub-millimeter voxel sizes to provide more anatomical details. However, despite improving resolution, decreasing the voxel size comes at the cost of higher noise levels (Haouimi & Yeung, 2011). Therefore, the use of high-resolution MRIs for brain morphometry and image segmentation in the context of improved resolution should consider the impact of the higher noise floor (Gordillo et al., 2013).

There are several methods for quantifying relative levels of noise in MRI, with signal-to-noise ratio (SNR) being one of the most commonly used metrics of image quality. SNR in MRI is usually obtained by computing the difference between a region of interest (ROI) selected from an image area containing anatomical information and the background, which should consist of only air, and thus, any signal present in it should be noise. The computed difference is divided by the standard deviation of the signal present in the background. While acquisition parameters can be modified to enhance SNR (Haouimi & Yeung, 2011), this improvement comes at the cost of increased acquisition time and requires additional post-processing steps. In contrast, denoising methods can generate high-SNR images without adding to acquisition time and costs. Therefore, denoising methods are a promising way to address low SNR in high-resolution MRIs, in particular for large datasets.

In MRI, the magnitude images are generated after measuring the real and imaginary signals through a quadrature detector. Each of these two components is contaminated with zero-centered white Gaussian noise. However, the mapping from the real and imaginary images to the magnitude image is non-linear, leading to a non-Gaussian distributed noise in the magnitude image (Edelstein et al., 1986).

Furthermore, each of the components of noise is assumed to be uncorrelated due to the Fourier transform. The amount of noise present in the image will depend on the static magnetic field, the sample volume size, the image voxel size, the receiver bandwidth, and the number of averages in the image acquisition (Edelstein et al., 1986). The probability distribution$p_{M}$for the measured pixel intensityMis given byGudbjartsson and Patz (1995:

$$\begin{array}{c} p_{M}\left(M\right)=\;\frac{M}{\sigma^{2}}e^{-\frac{M^{2}+A^{2}}{2\sigma^{2}}}I_{0}\left(\frac{AM}{\sigma^{2}}\right), \end{array}$$ (1)

where$I_{0}$is the modified zeroth order Bessel function of the first kind and σ denotes the standard deviation of the Gaussian noise in the real and the imaginary images—which is assumed to be equal—and A denotes the voxel intensity in the absence of noise. The equation above is also known as the Rice density, and it is far from being Gaussian for small SNR ($\frac{A}{\sigma }\;$≤ 1). However, for ratios$\frac{A}{\sigma }>3,$it approximates to a Gaussian distribution (Gudbjartsson & Patz, 1995). When the signal is zero (i.e., in the background of the MRI) and only noise is present,Ais 0 and$p_{M}\left(M\right)$describes a Rayleigh distribution. In contrast, when signal is large, then the noise can be considered Gaussian (Gudbjartsson & Patz, 1995):

$$\begin{array}{c} p_{M}\left(M\right)\approx \;\frac{1}{\sqrt{2\pi \sigma^{2}}}e^{-(M-\;\sqrt{A^{2}+\;\sigma^{2})}/2\sigma^{2}.} \end{array}$$ (2)

### 1.1 Related work

Most denoising tools that are currently used in the neuroimaging field involve non-deep learning (NDL) methods that are based on different techniques such as the sparseness of voxel patches and computing the self-similarity between blocks of voxels. Some examples include Adaptive Optimized Non-Local Means (AONLM), Non-Local Means (NLM), Pre-filtered Rotationally Invariant Non-Local Principal Component Analysis (PRI-NLPCA), Block-Matching 4D denoising (BM4D), and Pre-filtered Rotationally Invariant Non-Local Means (PRI-NLM) (Maggioni et al., 2013;Manjón et al., 2008,^2010^,^2012^,^2015^). Sparseness methods work especially well for low noise levels and low resolutions, whereas self-similarity methods work better when a good reference image is available. However, all NDL methods require significant processing times to achieve good results due to their sliding window nature.

Deep learning (DL) denoising methods generate a function capable of mapping a representation to a certain given image. Most DL methods employ Convolutional Neural Networks (CNNs), which learn the transformation mapping of noisy images and their corresponding noise-free counterparts. As universal function approximators, CNNs can learn these transformations through backpropagation. It is important to note that DL approaches have recently benefited from the use of dedicated graphical processing units (GPUs), which make them several orders of magnitude faster than NDL algorithms, even when used on CPUs instead of a dedicated GPU ( Jiang et al., 2018 ). Moreover, DL methods can outperform NDL methods in many medical image-denoising tasks ( Augustin et al., 2022 ; Gurrola-Ramos et al., 2021 ; Roy et al., 2021 ; Tripathi & Bag, 2020 ; K. Zhang et al., 2017 ). Despite the aforementioned advantages of using DL approaches for image denoising, they face the following drawbacks:

- Requiring large amounts of curated data: Having sufficient and representative training and validation data is crucial for optimal training. Adequate variability within the training data enables the network to generalize to unseen data instead of overfitting. The more representative the data used for training is, the better the performance that can be achieved.
- DL denoising methods may introduce undesired blurring on high-frequency details (e.g., cerebellar GM and WM-GM interfaces) due to their convolutional nature and the use of operations such as pooling. This can potentially lead to undesired loss of anatomical information or changes in the anatomy (Yang et al., 2022).
- Need to train on fixed-resolution images: To optimally train the weights in a CNN, images with different resolutions must be resampled to match the requirements of the network so that the anatomical structures are within approximately the same scale in pixel dimensions. Therefore, if it is necessary to denoise images at several resolutions, different networks should be trained for the different resolutions that one would like to denoise images at, limiting the practicality of DL denoising methods (Henschel et al., 2020,^2022^).
- Current hardware used for training DL denoising methods (specifically the GPU memory) does not allow for the handling of common brain structural MRIs. Therefore, there is a need to extract either smaller 3D blocks (Manjón & Coupe, 2018) or whole-size slices or stacks of slices (Jiang et al., 2018;Tripathi & Bag, 2020) referred to as 2.5D (Henschel et al., 2020,^2022^), so that the data fit in the GPU memory.

Different network architectures have been proposed for image denoising in the literature: from the popular ResNet-based method (He et al., 2015), DnCNN (K. Zhang et al., 2017), and its adaptation to MRI denoising MCDnCNN (Jiang et al., 2018), to U-Net-based methods (Ronneberger et al., 2015) for volumetric medical image denoising, as in RDUNet (Gurrola-Ramos et al., 2021), which showed a performance gain over DnCNN. However, it has been shown that a nested, densely connected version of U-Net, that is, U-Net++ (Zhou et al., 2018), can outperform the original U-Net in medical image segmentation tasks, suggesting that there is an improved image feature extraction at the latent space level with the nested approach that could also be used for other tasks such as regression, as in image denoising. Finally, residual CNNs and GANs have shown better performance in denoising tasks than non-residual approaches, since it is easier to learn the latent noise of the noisy input than learning to infer the clean image from it (K. Zhang et al., 2017).

Multiple DL approaches for brain MRI denoising exist in the literature.Jiang et al. (2018)proposed MCDnCNN, a multi-channel (2.5D) version of the DnCNN originally proposed byK. Zhang et al. (2017), in which a stack of 17 convolutional layers, batch normalization layers, and rectified linear units aimed to estimate the residual noise from the middle slice in the 2.5D stack of slices.Manjón and Coupe (2019)proposed a hybrid approach in which a CNN using sliding-window-like 3D patches is used to generate an initial denoised image, then the denoised image would be used as guide image in the final denoising process made by a non-local averaging approach, as an extension of their previous work (Manjón & Coupe, 2018) whose architecture was also based in the work ofJiang et al. (2018).Tripathi and Bag (2020)used a CNN architecture similar to UNet, in which an encoder–decoder network connected through a bottleneck with residual blocks is fed with a 2D noisy image, and the network estimates the residual noise of the input slice.Tian and Song (2021)proposed a conditional GAN in which 2D slices were fed to the generator network (UNet-like architecture), and the generator produced the clean slice. InPlassard et al. (2018)they proposed a denoising autoencoder with an encoder–decoder architecture with skip connections that aimed to generate a denoised image from a noisy 2D input.

In this study, we propose a new, robust method for denoising structural brain MRIs by employing a DL approach that can be used to denoise images from a wide variety of resolutions, scanner vendors, and field strengths, while maintaining the structural integrity of the high-frequency response. Furthermore, our proposed method allows users to obtain denoised images with similar peak SNR (PSNR) values to state-of-the-art denoising methods with often faster processing times when processed on a dedicated Graphical Processing Unit (GPU). We have made the proposed method as well as all its training and evaluation scripts and trained weights available athttps://github.com/waadgo/FONDUE.

## 2 Methods

### 2.1 Datasets

To obtain a robustly trained and generalizable CNN, sufficient brain images and feature variability are required for both training and validation. Therefore, for the methodology design, we included datasets acquired at different magnetic field strengths (1.5T and 3T), scanner vendors (SIEMENS, Phillips, and GE), acquisition protocols, resolutions (from over a millimeter voxel sizes to sub-millimeter voxel sizes), subject ages (from young adults to aged individuals), and diseases (healthy, Mild Cognitive Impairment, Alzheimer’s Disease, Parkinson’s Disease, Schizophrenia, etc.). We also included a balanced number of female and male participants in our dataset. Section A in the Supplementary Materials provides a brief description of the datasets that were used for training our proposed model.Table 1andSupplementary Table S1(see Section B inSupplementary Materials) include a summary of the characteristics of the images included for each dataset.

**Table 1.** Parameter acquisition for the datasets used for training, validating, and testing.

Study	Field strength	Scanner model	Repetition time [ms]	Echo time [ms]	Flip angle [°]	Voxel dimension [mm ^3^ ]
ABIDE-II	3T	Philips, GE, Siemens	3	3.9	8	Multiple
ADNI1	1.5T, 3T	Philips, GE, Siemens	~7–8	~2–4	~8–12	1.0 × 1.0 ×1.2
HCP	3T	Siemens	2.4	2.14	8	0.7 × 0.7 × 0.7
IXI	1.5T	GE, Philips	9.6	4.6	8	1.0 × 1.0 × 1.0
LA5c	3T	Siemens	1.9	2.26	7	1.0 × 1.0 × 1.0
MIRIAD	1.5T	GE	15	5.4	15	0.937 × 0.937 × 1.5
Custom_0.5	3T	Siemens	2750	2.2, 4.63	N/A	0.5 × 0.5 × 0.5
UH_250	7T	Siemens	3580	2.41	5	0.25 × 0.25 × 0.25
UH_500	7T	Siemens	2740	3.24	5	0.5 × 0.5 × 0.5
UH_1000	7T	Siemens	2500	1.91	5	1.0 × 1.0 × 1.0

### 2.2 System

To build FONDUE, we used Python 3.9.7 along with PyTorch 1.10.0, numpy 1.22.2, and nibabel 3.2.1 to handle usage of NIFTI and MGH imaging formats. The script was implemented using Anaconda’s Spyder editor in a Windows 11 system. Our hardware included an Intel Core i7-10700 as CPU, an NVIDIA RTX 3090 for GPU (24GB of VRAM), and 128GB of RAM memory.

### 2.3 Network architecture

**Fig. 1.**
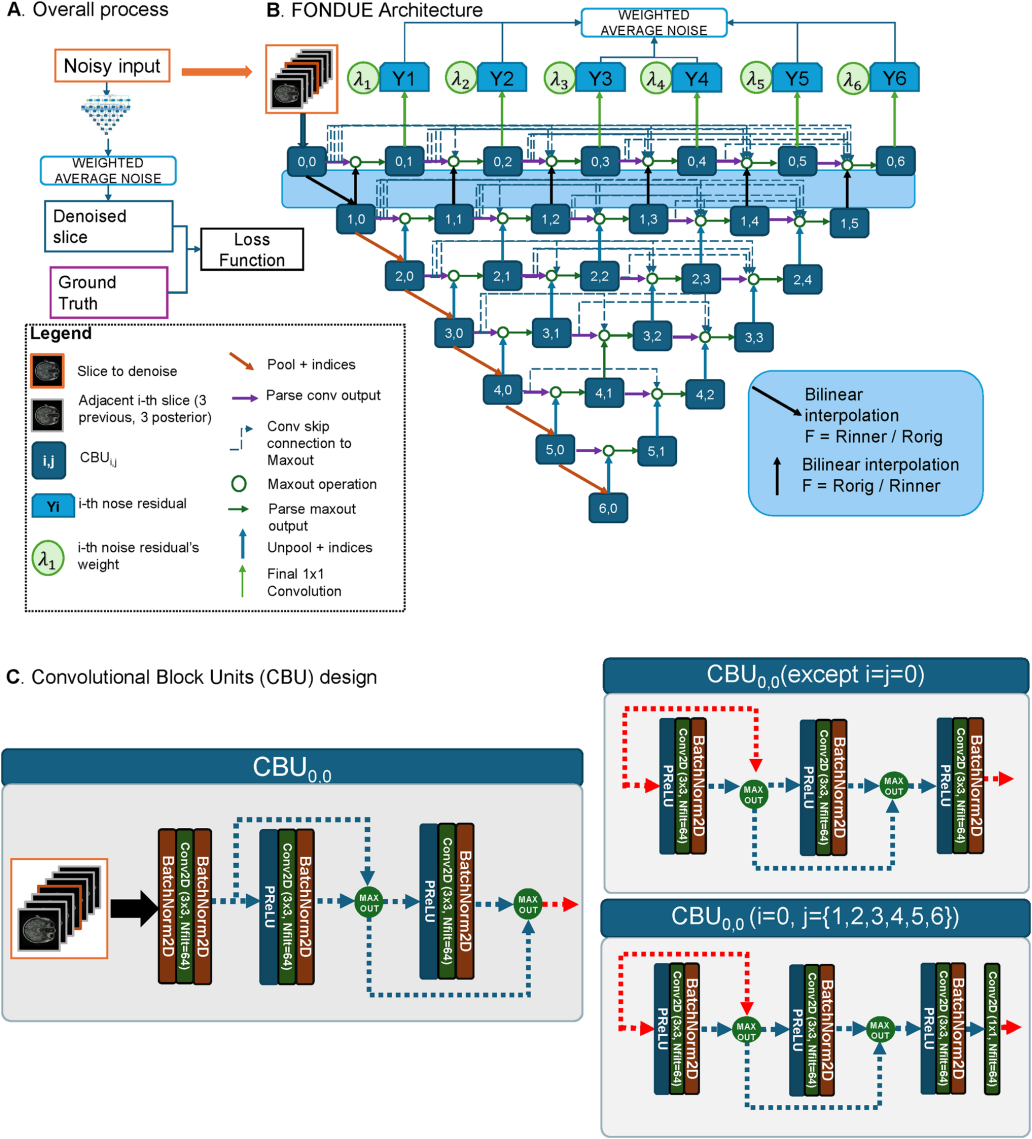
(A) Overview of the denoising process for FONDUE. A stack of seven slices is input into the network, which estimates the noise from the middle slice in the stack through a weighted average of its outputs. (B) Network architecture of the proposed FONDUE method. The network receives the stack of seven noisy slices to denoise the middle slice (orange box, orange and gray contours indicate the middle and adjacent slices, respectively), parsed to the$CBU_{0,0}$, which extracts the features at the native resolution. The normalization layer then rescales the feature maps to the latent inner resolution of 1.0 mm (downward black arrow) and from the latent inner resolution to the native resolution (upward black arrow) using bilinear interpolation. The maxout operation (green circle) receives the concatenated feature maps from two different sources: the previous immediateCBU(purple arrow) as well as all previousCBUs that are at the same level/latent resolution as the maxout operation (blue-dotted arrows), and the resolution-normalized (black arrows for layer 0) or the unpooled feature maps from the immediate inferior layer (blue arrows for layers 5 to 1). As a result, the maxout operation outputs only the$N_{filt}$feature maps that correspond to the features with the maximum value at each spatial location. The leftmostCBUs (encoder bone) parse the downscaled feature maps into the next layer ofCBUs through pooling (orange arrows). At the upper-most level$\left(CBU_{0,N}\;N=\left\{1,\;2,\;3,\;4,\;5,\;6\right\}\right)$, the native resolution$N_{filt}$feature maps are collapsed into one channel (green arrow), which corresponds to the noise candidate YN (blue rectangles). All the noise candidates are then averaged using their respective values of lambda ($\lambda_{\text{N}}$) to produce the final estimated noise (blue and white rectangle). The final estimated noise is then subtracted from the middle slice of the input stack to produce the denoised slice. The loss function receives the denoised slice and the ground truth. (C) Convolutional Block Units (CBU) design. For the input block$CBU_{0,0}$(left figure), the stack of slices is input to the network at native resolution and is parsed into two convolutional layers (green vertical blocks). The output of both convolutional layers is parsed into a maxout function (yellow circle). For the rest ofCBUblocks (top right figure), a series of three convolutional layers with PReLU non-linearities and batch normalization process the respective input. In the last convolutional block (bottom right figure), a 1 × 1 convolution collapses the feature maps into a single output channel.

Figure 1presents the architecture for the proposed FONDUE model. A stack of seven consecutive slices is input into the network to denoise the middle slice. The stack is a tensor of size BS × C × H × W, where BS denotes the batch size, C is the channels (7 in this case), and H and W are the Height and Width of every image being input into the network. The blue blocks denoted by “i,j” refer to the convolutional blocks CBU_i,j_, which are composed of a series of 2D convolutions with 3 × 3 kernels and padding of 1 (to maintain the spatial resolution within each of the CBU). The output to each convolution is parsed into a PReLU unit to introduce a non-linearity, and our CBU may or may not contain 2D batch normalization layers, for which we have tested the implication of the inclusion of the latter unit in the ablation study made inSection 3. For the cases in which batch normalization is included, the CBU blocks are exactly the Competitive Dense Blocks (CBDs) first proposed inEstrada et al. (2018)and adopted byHenschel et al. (2020). The dotted arrows refer to the skip connections which allow for the latent information sharing as described inZhou et al. (2018). The circle denotes the maxout operation, first proposed in MaxOut networks (Goodfellow, I.J. et al., 2013) and adapted to CDBs inEstrada et al. (2018). It allows for local feature competition, in which only the most relevant feature in a given spatial location of the feature maps will be preserved. This approach makes it feasible to train a deep neural network with high-resolution images while preventing a GPU memory overflow since the number of filters is kept throughout the encoder and decoder stages of the network (especially important for MR images) (Henschel et al., 2020,^2022^). The purple arrows denote a simple parsing to the maxout operation from the immediate previous CBU. The black arrows denote the bilinear interpolation operations part of the resolution-normalization step as described in VINNs (Henschel et al., 2020,^2022^), which allows normalizing the voxel size of the input in the latent space, which is related to the concept of feature augmentation first described inDeVries and Taylor (2017)that aims to improve generalization capabilities of a network by introducing random augmentations in latent space rather than the input. This operation is crucial since it allows the learning of features in a normalized image-resolution space while being able to still get native space features used by the first row of CBUs. The orange arrows are the MaxPool2d operations plus indices parsing (accounting for the downscaling operation before going down through the encoder part of the network), and the light-blue arrows pointing upward denote the MaxUnpool2d operations that use the indices previously parsed (to perform the upsampling of the feature maps, which allows the concatenation with the output of the upper-level CBUs). Finally, the red arrows denote the final convolution step with a kernel of size BS × C × H × W and the blue YN units with N = {1,2,3,4,5,6} are the six noise residuals (the six estimated residual noise maps, i.e., the estimated noise extracted from the noisy input) produced by all the sub-networks of FONDUE. This final convolution step (green arrow) performs the conversion from latent space (multiple channels) to the native image space (only one channel) containing the predicted noise residuals. The weighted average of all noise residuals (YN) images is subtracted from the noisy middle slice to produce the denoised image.

Similar to UNet++ (Zhou et al., 2018), we have adopted a densely connected U-shaped encoder–decoder architecture. As opposed to UNet and UNet++, we do not increase the number of filters as we go deeper into the network. We instead maintained the same number of filters at any position of the network, using maxout operations as proposed inHenschel et al. (2020,^2022^) since it helps to significantly reduce the computational load while keeping good performance levels. We took advantage of maxout’s competitive nature to obtain a fast, reliable method that can be widely applicable and used. Additionally, we used convolutional block units (CBUs) similar to the Competitive Dense Blocks (CDB) proposed byEstrada et al. (2018).

It is worth noticing that the internal resolution normalization with an Ri = 1.0 (mm), the usage of a stack of seven consecutive slices as input to the network in a 2.5D manner, keeping the same number of filters throughout the network’s CBUs, and the usage of inSA were ideas directly taken fromHenschel et al. (2020,^2022^). The overall layout of our CBUs was straightly taken from CBD (Henschel et al., 2020,^2022^) with the main observation that we have tested the performances with and without the batch normalization layers since other image regression tasks have reported that improvements in final performance and training speed were gained when removing this layer (Manjón & Coupe, 2019;Umer et al., 2020;Umer & Micheloni, 2020). They have pointed out that the normalization process produces artifacts on the output image, and also that these types of layers work differently during training and inference, causing a performance gap between training and inference. During training, mean and variance statistics are computed over the whole training set, favoring stable training and better convergence. In an ideal case, the batches are large and representative enough to keep the mean and variance estimates stable during each iteration during training. However, since we are using small batch sizes during training, these parameters (mean and variance) tend to lead to sub-optimal performance during inference.

After${\text{CBU}}_{0,0}$, the feature maps are resampled by performing bi-linear interpolation using a factorF=RoRi, where$R_{o}$is the original resolution in terms of the voxel size in millimeters and$R_{i}$is the internal resolution of the network, which in this case was set to 1.0, but can be adjusted to any desired value. After filter bilinear interpolation, the resulting feature maps are fed into the${\text{CBU}}_{1,0}$. We then consecutively compute${\text{CBU}}_{\text{i},\text{j}}$withi={2, 3, 4, 5, 6}andj={0}, performing a MaxPooling operation with kernel size = (h, w) with h = 2 and w = 2, stride = 2 and padding = 0. Each MaxPooling operation reduces the dimensions of the filters to half.${\text{CBU}}_{\text{i},\text{j}}$withi={2, 3, 4, 5}andj=[1, 6-i] is computed by performing the maxout operation between the MaxUnpool2d operation of${\text{CBU}}_{\text{i}+1,\text{j}-1}$and all${\text{CBU}}_{\text{i},\left[0,\text{j}-1\right]}$. Finally, we compute${\text{CBU}}_{\text{i},\text{j}}$withi=0andj=[1, 6]by performing maxout of the bilinearly interpolated CBU_i+1,j-1_(using a factor of1F, i.e.,RiRo) and all$CBU_{i,\left[0,j-1\right]}$.

### 2.4 Weighted average of noise candidates

We trained six scalar parameters as weights for each of the noise candidates YN called$\lambda_{N}$with N∈{1, 2, 3, 4, 5, 6}. The final noise candidate Y is the weighted average of all six noise candidates Y_N_:

$$\begin{array}{c} Y=\;\frac{1}{\lambda }\sum_{N=1}^{6}\lambda_{N}YN, \end{array}$$ (3)

with

$$\begin{array}{c} \lambda =\sum_{N=1}^{6}\lambda_{N}. \end{array}$$ (4)

### 2.5 View aggregation

During training of FONDUE, the dataset contained only axial slices. However, during inference, there were visible artifacts on the final denoised volume (visible in the coronal and sagittal planes) when the 3D volume was denoising using only axial slices. Therefore, as proposed to FastSurfer and FastSurferVINN (Henschel et al., 2020,^2022^), we employed a view aggregation strategy, in which the 3D input volume is processed by the CNN three times; that is, once per anatomical plane. A 3D output with the denoised output is then obtained for each—axial, coronal, and sagittal—plane, and the final output is produced after averaging each of these three volumes. In contrast with the original view aggregation technique inHenschel et al. (2020,^2022^), we only have one set of weights produced during the training using axial slices, and during inference, we use this same set of weights for processing all—axial, coronal, and sagittal—planes, and we made a simple average of the three denoised volumes generated in the view aggregation stage. This produced better quality images and removed artifacts as reported in FastSurfer and FastSurferVINN.

### 2.6 Loss functions and image similarity metrics

The following image similarity metrics were used to evaluate the performance of FONDUE and allow for comparison against other denoising methods in the literature.

#### 2.6.1 Mean absolute error (MAE) and mean squared error (MSE)

$$\begin{array}{c} MAE\left(x,y\right)=L1=\;\frac{1}{mn}\sum_{i=0}^{m-1}\sum_{j=0}^{n-1}\left[x\left(i,j\right)-y\left(i,j\right)\right] \end{array}$$ (5)

$$\begin{array}{c} MSE\left(x,y\right)=L2=\;\frac{1}{mn}\sum_{i=0}^{m-1}\sum_{j=0}^{n-1}{\left[x\left(i,j\right)-y\left(i,j\right)\right]}^{2}. \end{array}$$ (6)

#### 2.6.2 Structural similarity index metric (SSIM) and multi-scale SSIM (MSSSIM)

$$\begin{array}{c} SSIM\left(x,y\right)=\;\frac{\left(2\mu_{x}\mu_{y}+c_{1}\right)\left(2\sigma_{xy}+c_{2}\right)}{\left(\mu_{x}^{2}+\;\mu_{y}^{2}+c_{1}\right)\left(\sigma_{x}^{2}+\sigma_{y}^{2}+c_{2}\right)}, \end{array}$$ (7)

where

- $\mu_{x}$and$\mu_{y}$are the pixel-wise mean values of x and y
- $\sigma_{xy}$covariance between x and y
- $\sigma_{x}^{2}$and$\sigma_{y}^{2}$are the variances of x and y
- $c_{1}=\;{\left(k_{1}L\right)}^{2}$and$c_{2}=\;{\left(k_{2}L\right)}^{2},\;L=\;2^{\#bits\_per\_pixel}$and$k_{1}=0.01$and$k_{2}=0.03.$

$$\begin{array}{c} l\left(x,y\right)=\;\frac{2\mu_{x}\mu_{y}+c_{1}}{\mu_{x}^{2}+\;\mu_{y}^{2}+c_{1}} \end{array}$$ (8)

$$\begin{array}{c} c\left(x,y\right)=\;\frac{2\sigma_{x}\sigma_{y}+c_{2}}{\sigma_{x}^{2}+\;\sigma_{y}^{2}+c_{2}} \end{array}$$ (9)

$$\begin{array}{c} s\left(x,y\right)=\;\frac{\sigma_{xy}+c_{3}}{\sigma_{x}\sigma_{y}+c_{3}} \end{array}$$ (10)

$$\begin{array}{c} \begin{array}{l} MSSSIM\left(x,y\right)={\left[l_{M}\left(x,y\right)\right]}^{\alpha M}\prod_{J=1}^{M}{\left[c_{j}\left(x,y\right)\right]}^{\beta j}\\ \;\;\;\;\;\;\;\;\;\;\;\;\;\;\;\;\;\;\;\;\;\;\;\;\;\;{\left[s_{j}\left(x,y\right)\right]}^{\gamma \;j}, \end{array} \end{array}$$ (11)

where

- l(x,y),c(x,y),ands(x,y)are luminance, contrast, and structural similarity measures.
- $\beta_{1}=\;\gamma_{1}=0.0448$,$\beta_{2}=\;\gamma_{2}=0.2856$,$\beta_{3}=\;\gamma_{3}=0.3001$,$\beta_{4}=\;\gamma_{4}=0.2363$,$\beta_{5}=\;\gamma_{5}=\;\alpha_{5}=0.1333.$
- Best results for M = 2.

#### 2.6.3 Learned perceptual image patch similarity (LPIPS) and feature loss ${\text{L}}_{\text{feature}}^{\text{l}}$

$$\begin{array}{c} LPIPS\left(x,\;y\right)=\;\frac{1}{N_{l}}\sum_{1}^{N_{l}}{\left\|\Phi_{l}\left(x\right)-\Phi_{l}\left(y\right)\right\|}_{2}^{2}. \end{array}$$ (12)

In addition to LPIPS, we used a feature loss metric based on the VGG-16 network, previously described in the literature (named after Visual Geometry Group network with 16 layers of weights) as feature extraction method (Mehdizadeh et al., 2021):

$$\begin{array}{c} L_{feature}^{l}\left(x,\;y\right)=\;\frac{1}{N_{l}}\sum_{1}^{N_{l}}\left|\Phi_{l}\left(x\right)-\Phi_{l}\left(y\right)\right|, \end{array}$$ (13)

wherexandyare the two images to compare,$\Phi_{l}$are the pre-trained weights of a CNN at a given convolutional layerl, and$N_{l}$is the number of pixels atl. We used the*vgg16*pre-trained weights from the*torchvision.models*module, withl # {1}.

#### 2.6.4 Peak signal-to-noise ratio (PSNR)

$$\begin{array}{c} PSNR\left(x,y\right)=10\;log_{10}\left(\frac{MAX_{I}^{2}}{MSE\left(x,y\right)}\right), \end{array}$$ (14)

where$MAX_{I}$is the maximum voxel intensity value (e.g., 255 for unit 8 image format), andMSE(x,y)is defined inEquation (6).

#### 2.6.5 On the election of the performance metrics

As described in previous sections, multiple metrics were used to measure similarity between the images in different ways, to reflect different aspects of image quality, and enable comparisons with the literature. MAE (L1) and MSE (L2) are widely used loss functions for denoising CNN (Gurrola-Ramos et al., 2021;Jiang et al., 2018;Manjón & Coupe, 2018;Tian & Song, 2021;Tripathi & Bag, 2020;Yang et al., 2022;K. Zhang et al., 2017) and measure the average distance in terms of pixel intensity between two images. While computationally inexpensive, they are prone to producing blurring in the output images since they are not blur invariant. Surprisingly, MAE is known to produce better results than MSE (Ding et al., 2021). PSNR is intuitively one of the most widely used performance metrics in image denoising methods (both DL and non-DL) since it is a measure of how different two images are in a pixel-wise manner. Although PSNR is sensitive to the presence of noise, it is not invariant to pixel shifts or blur (Mehdizadeh et al., 2021), and may not represent how human vision perceives similarity. SSIM was proposed to measure the image structure in terms of luminance, contrast, and structure statistics, while MSSSIM is an extension of SSIM that measures the structural similarity at different resolutions transforming the input images into Gaussian pyramids (Ding et al., 2021). Finally, LPIPS is one of the first deep-feature similarity metrics that proposed producing a metric capable of computing image similarity based on CNNs. Specifically, they found out that by comparing the feature maps in the latent space of two image patches using a VGG network trained on a naturalistic image dataset, the produced average metric better resembles human vision even in the presence of multiple distortions (R. Zhang et al., 2018). As such, all feature maps$f_{i}\left(I_{A}\right)$produced by the filters in the feature extraction network for the image I_A_are compared against the same feature map f_i_from the image$I_{B}\;\left(f_{i}\left(I_{B}\right)\right)$through an MSE operation. Therefore, each of the feature maps plays a role in the deep-feature extraction to compute the similarity metric, as opposed to the pixel-wise similarity metrics in which the difference is computed in the pixel space. Based on this information, we opted for assessing the performance with the commonly used metrics PSNR and SSIM, but also included MS-SSIM and LPIPS, as they are known to be more robust in the presence of distortions.

### 2.7 Statistical analysis

To perform statistical significance tests between the compared methods, we have used RStudio 2023.12.1 Build 402 for paired t-tests. The resulting p-values were then corrected for multiple comparisons using the Benjamini–Hochberg to correct for false discovery ratio (FDR).

### 2.8 Data preprocessing

As discussed previously, all MRIs inherently contain a certain level of noise. This is also the case for all the datasets to be used for training and testing FONDUE (with the exemption of the IXI dataset, which contains virtually zero noise as noted inManjón & Coupe (2018)). The first step in our preprocessing pipeline is to generate surrogates of noise-free MRIs by using a state-of-the-art denoising algorithm. In this case, we used the PRI-NLPCA algorithm proposed byManjón et al. (2015), which is a method that effectively filters noisy MRIs to produce denoised images. Using denoised images as ground truth is standard practice and PRI-NLPCA was used to generate noise-free surrogates since it has been shown to outperform other widely known methods such as BM4D and NLM (Manjón et al., 2015). We did not perform bias field correction at any point as we wanted the network to learn denoising of both, corrected and uncorrected MRIs. The following additional steps were performed:

- The volumes were resampled to isotropic size (equivalent to the minimum voxel size in the three dimensions) and number of voxels (equivalent to the maximum dimension in the three dimensions) in all three dimensions. In other words, an image with a voxel size of v1, v2, and v3 and dimensions of H, W, and C was resampled to an image with an isotropic voxel size of min(v1,v2,v3) mm^3^and a dimension of max(H, W, C). Resampling operations were made using PyTorch’s interpolate method using “bilinear” mode, without aligning corners and without recomputing the scaling factor.
- The volumes were transformed to have the standard RAS orientation.
- The voxel values were rescaled to unsigned 8-bit integer values in range [0, 255] using a histogram-based approach with 1000 bins in the histogram, cropping the one thousandth most intense voxels to account for possible outliers. The algorithm with the previous three steps is known altogether as “conforming,” and we used an adaptation to Python by Martin Reuter extracted fromHenschel et al. (2020)and originally proposed by FreeSurfer under an Apache License, Version 2.0. Finally, the images were linearly cast to type single and range [0, 1].

### 2.9 Training settings

Using a one-cycle learning rate combined with high learning rates and annealing (such as cosine annealing), one could achieve training convergence considerably faster (Smith & Topin, 2017). This approach (called Super Convergence) has been used to reduce training time in various applications (Al-Kababji et al., 2022;Smith & Topin, 2017). According to the Super Convergence strategy, OneCycleLR scheduler from PyTorch library was used with the following parameters: max_lr = 0.01 cosine annealing, cycle_momentum = True, pct_start = 0.075, div_factor = 10, and final_div_factor = 100. AdamW optimizer was used with β_1_= 0.9 and β_2_= 0.99. As reported inGugger and Howard (2018), AdamW is superior to Adam and SGD when adopting a Super Convergence one-cycle learning rate policy. Finally, we used PyTorch’s automatic mixed precision through*torch.cuda.amp.autocast()*. This strategy automatically detects the variables and operations that can be done using single precision instead of double precision. This strategy enables lighter and faster training while maintaining similar accuracy to using double-precision variables and nearly halves CUDA memory usage.

#### 2.9.1 Comparing multiple designs: One-step and two-step approaches

We are investigating multiple design settings for the proposed denoising algorithm. First, based on the observations ofMehdizadeh et al. (2021), we wanted to compare whether a pretrained feature extraction network based on VGG-16 or LPIPS would work better as loss functions. The network based on VGG-16 usingEquation (13)was named FONDUE_A, while the one using LPIPS withEquation (12)was named FONDUE_B. Furthermore, inspired on different deep learning implementations for different computer vision applications such as brain MRI denoising (Manjón & Coupe, 2019), text-to-image generation (Ramesh et al., 2022;Rombach et al., 2022), and image inpainting (Rombach et al., 2022), in which they used two or more stages (DL or NDL) for generating the final image, we tested whether the two stand-alone denoising approaches (FONDUE_A or FONDUE_B) would get benefitted from adding a subsequent refinement network. We called the subsequent networks FONDUE_B1 and FONDUE_B2 (a summary of the features of each FONDUE version can be found inSupplementary Table S2). These networks receive the output of FONDUE_B as input, and only differ in the level of noise that they handle: FONDUE_B1 is trained on low-mid noise levels, whereas FONDUE_B2 is trained on mid-to-high noise levels. Finally, as noted byZ. Wang et al. (2020), in other image-to-image translation methods such as super-resolution and deblurring, removing batch normalization layers from the convolutional blocks improved generalization, sped-up training, and prevented artifacts in the output images. As such, we performed an ablation study on whether removing batch normalization from the stand-alone or two-stage networks improved performance.

#### 2.9.2 Stand-alone networks

The stand-alone networks refer to those intended to be used without any pre- or post- processing (i.e., unlike FONDUE_B1 and FONDUE_B2). These are FONDUE_A_BN (with batch normalization), FONDUE_A_noBN (without batch normalization), FONDUE_B_BN (with batch normalization), and FONDUE_B_noBN (without batch normalization). These four networks share the training strategy using Super Convergence described inSection 2.4. These networks were trained with consistent parameters and compared against each other to assess which of the two deep-feature loss functions would be optimal, and how batch normalization affects the performance of the networks in different scenarios. These four networks were considered prototypes and were all trained for 10 epochs. The best performing combination was then trained for 50 epochs, using the same training strategies (referred to as FONDUE_LT). Finally, we also trained two additional networks with the same training parameters and datasets (UNET-VINN and MCDnCNN, described in detail below), and compared them against the prototype versions of FONDUE.

##### 2.9.2.1 FONDUE_A

For the two versions of FONDUE_A,$L_{feature}^{l}$was used as a loss function as described inEquation (13)to preserve the fine details of the denoised images using OneCycleLR learning rate scheduler. The full training set was used as described inSupplementary Table S1.

##### 2.9.2.2 FONDUE_B

Similar to FONDUE_A, FONDUE_B was trained for 10 epochs with OneCycleLR, using LPIPS loss as described inEquation (10). The full training set was used as described inSupplementary Table S1.

##### 2.9.2.3 FONDUE_LT

This version of FONDUE was trained for a total of 50 epochs, with the same training scheduler OneCycleLR as FONDUE_A and FONDUE_B. The prototype version of FONDUE that worked best was FONDUE_B without batch normalization, and we adopted this combination of loss function (LPIPS) with a non-batch-normalized architecture for FONDUE_LT.

##### 2.9.2.4 FONDUE_LT++

The original version of UNET++ (Zhou et al., 2018) doubles the number of filters as the spatial resolution of the filters gets halved. However, FONDUE architecture keeps the number of filters constant throughout the network to maintain memory consumption and processing times as low as possible. In FONDUE_LT++, we have modified the FONDUE network architecture to double the number of filters in a similar manner as the UNET++ architecture, that is, starting with 64 filters in the CBU_0,j_and CBU_1,j_with j ∈ {0, 1, 2, 3, 4, 5, 6}, whereas for the deeper CBU_i,j_units (i ≥ 2), the number of filters gets duplicated as their spatial resolution gets halved. We trained FONDUE_LT++ for 50 epochs using the same training parameters, scheduler, and optimizer as FONDUE_LT. However, we had to reduce the learning rate by a factor of 10 since the weights became “NaN” after 4 epochs, indicating instability and the need to reduce the learning rate. FONDUE_LT++ follows the overall architecture described inFigure 1.

##### 2.9.2.5 UNET-VINN

This network architecture was based on a basic UNet (Ronneberger et al., 2015), in which an encoder (downscaling) backbone is connected to a decoder (upscaling) through a bottleneck unit and through concatenating skip connections and in a residual manner (the output is subtracted from the input). Similar to FONDUE, a seven-channel tensor consisting of the slice of interest and the six proximal neighbor slices (three pre- and three post-) was used. Instead of using single convolutions (as is the case in basic UNet), we adopted the same CBUs as those in FONDUE (seeFig. 1), in which the initial CBU_Input_had 32 filters of size 3 × 3, is followed by a PReLU unit, another convolution, a maxout operation, a second PReLU unit, and a third and final convolution. After the initial CBUInput, the resolution normalization step through VINN (Henschel et al., 2022) is made using an inner resolution of 1.0 mm the same way that is done in FONDUE networks. The CBUs are similar to those in FONDUE (seeFig. 1), but the number of filters is doubled between each CBU. The downscaling step (in the encoder) is made through a*MaxPool2d*operation, keeping the indices for its utilization in the decoder with a*MaxPool2d*operation. In the deepest CBU (bottleneck), the convolutions are made with 512 filters (16 times the filters at 1/16 the size per feature map side). Therefore, the network has five levels (including the bottleneck). In the final CBU, a1×12D convolution is made to produce the noise estimate that will be subtracted from the middle slice of the input to generate the clean output.

##### 2.9.2.6 MCDnCNN

This denoising network (Jiang et al., 2018) was trained using a configuration similar to the original work. Seventeen stacked layers of 2D convolution, batch normalization, and ReLU nonlinearity were employed with uniform convolutions of3×3size and 64 filters, using padding of 1 to keep the tensor dimensionality throughout the network. The input channel is kept as seven (consistent with all other DL methods) and the network is trained in a residual manner, that is, the output of the network is the estimated noise, which is then subtracted from the middle slice from the input to generate a denoised slice.

#### 2.9.3 Second-stage networks

The second-stage networks were intended to work using the output of FONDUE_B networks and further processing them to attempt improving the denoising. The second-stage networks (FONDUE_B1_BN, FONDUE_B1_NOBN, FONDUE_B2_BN, and FONDUE_B2_NOBN) were trained using transfer learning with a fixed learning rate of 1e-6 and LPIPS as the loss function for two epochs. Specifically, the weights of the FONDUE_B model were used as the initialization, with the only modification being the type of noise applied during training (as described in Sections 2.10.2 and 2.10.3).

##### 2.9.3.1 FONDUE_B1 and FONDUE_B2

For these FONDUE versions, OneCycleLR was used for training along with and LPIPS as a loss function, also using the shorter version of the training set for two epochs.

### 2.10 Data augmentation

Data augmentation included random horizontal flips and random rotations ranging between [-5 and 5] degrees. In addition, we adopted the inSA (inner scale augmentation) technique in FastSurferVINN (Henschel et al., 2022) on all versions of FONDUE and also on the UNET-VINN network. This scale augmentation step consists of bilinearly resampling the images to a fixed resolution inside the network, and the resolution was modified by adding a µ value to the rescaling value. µ was set as a random number with a mean of zero and a standard deviation of 0.1. This introduction of scale augmentation can help the network achieve voxel-size invariance.

#### 2.10.1 Noise added to FONDUE_A, FONDUE_B, and FONDUE_LT networks

Variable synthetic Rician noise was added to the training images with sigma values ranging randomly between 0.0% and 9.0%. The variable field consisted of a ×3 multiplier in the center of the image volume and a ×1 in the outermost voxels, using cubic interpolation for intermediate voxels (i.e., maximum standard deviation was 27%).

#### 2.10.2 Noise added to FONDUE_B1 networks

For this refinement version of FONDUE, static Rician noise ranging between 0.0% and 4.0% was used for two epochs. Therefore, during training, each sample was denoised using FONDUE_B with these noise levels, and the output image of FONDUE_B was introduced into FONDUE_B1.

#### 2.10.3 Noise added to FONDUE_B2 networks

In this version, high noise levels (instead of low noise levels) of static Rician noise were used, ranging from 4.0% to 9.0%. Similarly to FONDUE_B1, this network inputs the noisy images into the pre-trained FONDUE_B and uses the denoised output of FONDUE_B as input into FONDUE_B2.

### 2.11 Data and code availability statement

Data used for training and validation were obtained from several open access repositories, listed inSupplementary Table S3.

The source code for FONDUE, installation instructions, and all pre-trained models is available athttps://github.com/waadgo/FONDUE.

### 2.12 Performance evaluation

The performance of FONDUE was compared against five widely used state-of-the-art denoising methods, namely AONLM, MRONLM, ODCT, ONLM, and PRINLM, in four different settings (Alkinani & El-Sakka, 2017;Manjón et al., 2008,^2010^,^2012^,^2015^): (1). Using held-out images from the same datasets FONDUE was trained on; (2). Using high-quality ground-truth images of high resolution (0.5 mm^3^) acquired by averaging 20 repetitions of the same image for 2 participants; (3). Using the BrainWeb (Collins et al., 1998) phantom with different levels of stationary Rician noise to enable comparisons with other published work in the literature; and (4). Using multi-resolution (1 mm^3^, 0.5 mm^3^, and 0.25 mm^3^) data acquired with the previously unseen field of strength (7T) and scanner model obtained from the UH_1000, UH_500, and UH_250, respectively (Lüsebrink et al., 2017,^2018^). Finally, processing times were compared across different methods to provide a benchmark for the performance speed of FONDUE.

### 2.13 Visual assessments

Three axial, coronal, and sagittal slices were extracted at 30%, 50%, and 70% of the axis length from the UH_NNN dataset images (UH_250, UH_500, and UH_1000) denoised using each of the methods in stand-alone and two-stage networks. A grid of shuffled images was generated, assigning a random letter to each image (seeSupplementary Fig. S3), and the raters were asked to visually inspect each of the images, assessing evident noise, preservation of anatomical structures, and overall image quality. Two raters (M.D. and A.B.) with extensive previous experience in image processing and visual quality assessment then independently ranked the top three denoised images for each resolution/dataset and type of denoising method (stand-alone/refinement). Note that the raters were blind to the information on the methods assessed at the time of rating.

## 3 Results

**Lambda values:**Supplementary Table S4presents the obtained$\lambda_{N}$values. The variability across these estimated values indicates that the noise sub-candidates contribute in a different manner to produce the best possible noise final estimation. Surprisingly, the main contributions to the final weighted average were never dominated by a single λ value, suggesting that the initial and intermediate output residuals Y_N_are not negligibly contributing to the optimal output, but they provide with necessary information to the ideal final residual.

### 3.1 Performance on test datasets

To ensure that FONDUE does not overfit to the images that were used for training, its denoising performance was assessed on never-seen-before images (test dataset) from the same six public datasets that were used for training. More specifically, 20 images were used per dataset for ABIDE-II, ADNI1, HCP, IXI, and LA5c, and 14 images from the MIRIAD dataset. This independent test set was used for all the sub-sections withinSection 3.1. The demographic information and image metadata were used to divide the test set according to their diagnosis (healthy vs. diagnostic groups), scanner vendor, resolution, and magnetic field used, in the following sub-sections. The summarized performance of the tested methods in terms of LPIPS across cohorts and stationary Rician noise levels of contamination are shown inFigure 2.

**Fig. 2.**
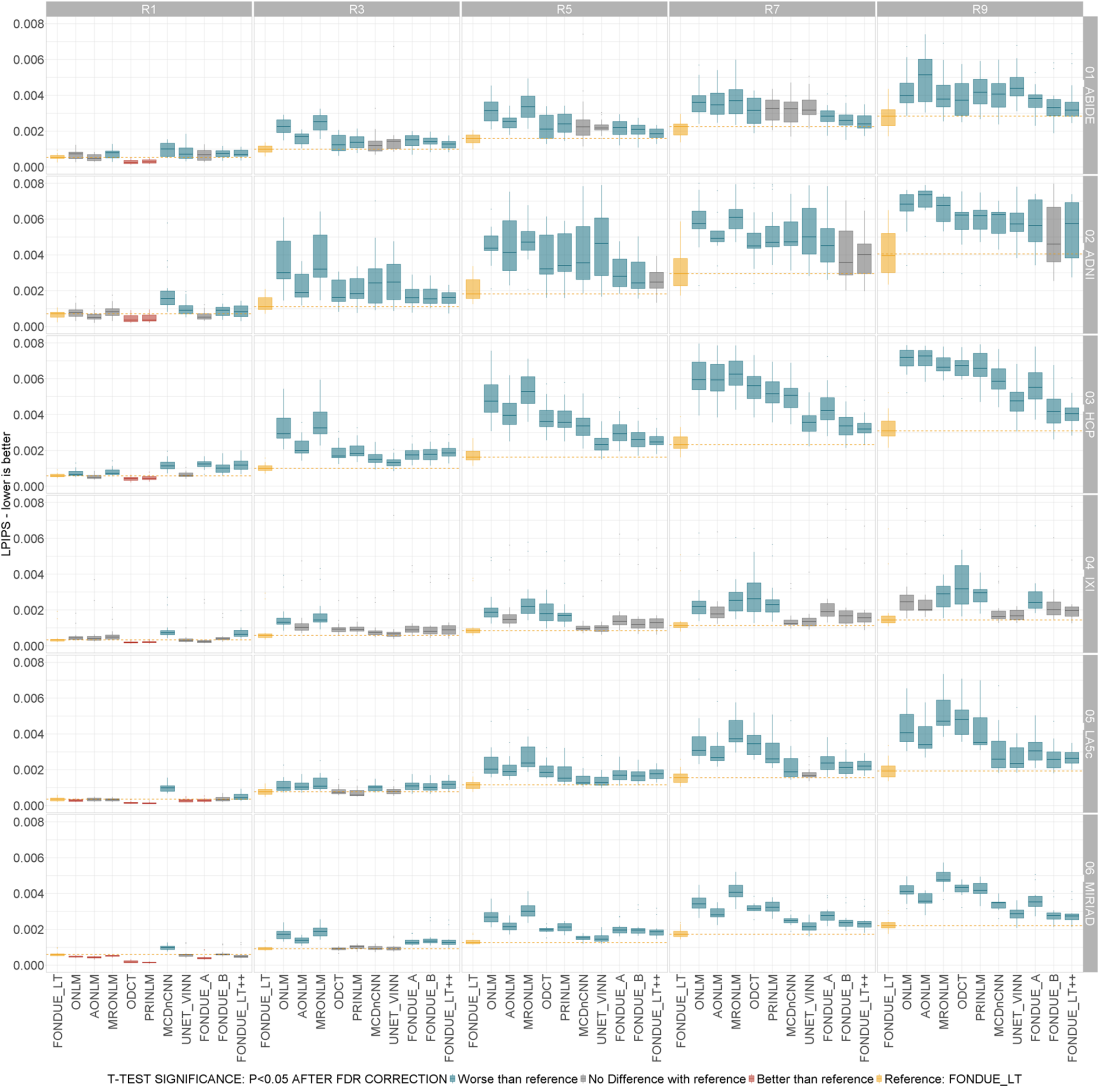
Overall performance of the compared methods on public datasets and stationary Rician noise values from 1% to 9% (as indicated with headers R1–R9 correspondingly from left to right). From top to bottom, rows indicate ABIDE-II, ADNI1, HCP, IXI, LA5c, and MIRIAD. t-Test color code: Yellow: reference method (FONDUE_LT). Blue: method significantly worse than reference. Red: method significantly better than reference. Gray: no significant differences found with respect to reference method. All the p-values in the sub-plots were corrected for multiple comparisons using the FDR method.

#### 3.1.1 Ablation studies

We have compared the performance of different design choices for FONDUE, which are detailed in Section C of Supplementary Materials. Non-batch-normalized networks showed a relatively better performance than their batch-normalized counterparts, the latter produced slightly blurrier images which might favor PSNR, SSIM, and MS-SSIM metrics, but worse in terms of LPIPS (Section C.1 inSupplementary Materials).

When it comes to comparing the two used deep-feature functions, FONDUE_A showed relatively good performance in the non-LPIPS performance metrics compared with FONDUE_B, but the texture of the images can be noted as blurrier and less similar to a noise-free surrogate. LPIPS showed to be a more robust metric with better agreement with perceived image quality as can be noted inSupplementary Figure S1.

FONDUE_LT showed a significant improvement over FONDUE_B, which is expected after training for 5× longer time as noted in Section C.4 inSupplementary Materials.

Finally, two-stage networks (including the usage of FONDUE_LT recursively as FONDUE_LT_×2) showed no improvements over their one-stage network counterparts, despite showing an apparent boost in the images with high levels of noise contamination. This apparent boost appears to be driven by an overall blurring effect introduced by the sequential use of these networks as can be noted inSupplementary Tables S9 to S.14.

#### 3.1.2 Comparisons across all methods, datasets, and noise levels

In this section, we compared the performance of the main FONDUE networks against other widely used denoising methods described in theSection 1: AONLM, MRONLM, ONLM, ODCT, and PRINLM. Since we have already shown the lower performance of the second-stage networks on these test set images, the analyses on this section exclude the results for those networks (FONDUE_B1, FONDUE_B2, and FONDUE_LT_X2—the recursive version of FONDUE_LT) for simplicity purposes, including only the non-batch-normalized networks.

##### 3.1.2.1 LPIPS

Performing paired t-tests across methods followed by FDR correction, our results suggest that ODCT and PRINLM were consistently the best across the methods tested for 1% of noise level (both stationary and variable) in the test datasets. Specifically, ODCT was the best method for ABIDE, ADNI, HCP, and IXI, while for MIRIAD and IXI, PRINLM significantly outperformed the rest of the methods tested. For the rest of the synthetic noise levels tested, FONDUE_LT was most frequently the best method or second best without statistically significant differences to the best method. For noises between 3% and 9% (for both stationary and variable noise), FONDUE_LT was the best method across all datasets.

##### 3.1.2.2 PSNR

Similar to LPIPS, PRINLM was the best performing method at 1% of noise level (both stationary and variable) across all datasets, with ODCT in second place with no statistical significance for ADNI, and ONLM was in second place for the rest of datasets at this noise level. FONDUE_LT was the best method consistently for all datasets for static and variable noises between 3% and 9%.

##### 3.1.2.3 SSIM

At 1% noise level, the best method for ADNI (both stationary and variable) and IXI (stationary noise) was ODCT (with no significant difference to PRINLM in second place), while for IXI, at 1% of variable noise, the best method was FONDUE_LT. For HCP, the best method was PRINLM for 1% noise (both stationary and variable), while for LA5c, PRINLM and ONLM performed best. For MIRIAD with both stationary and variable noise at 1%, the best method was PRINLM. Finally, for noises between 3% and 9% (stationary and variable), FONDUE_LT was the best method across all datasets.

##### 3.1.2.4 MS-SSIM

For 1% noise level, PRINLM was the best method across all datasets for both stationary and variable noises, with the sole exception of LA5c dataset, for which the best method was ONLM, followed by MRONLM (with non-significant differences). For noise levels between 3% and 9%, the best performing method was consistently FONDUE_LT across all datasets.

#### 3.1.3 Denoising performance: Grouping across variables of interest

##### 3.1.3.1 Pathological versus cognitively normal

Brain-related disorders can potentially affect the quality of the images as certain diseases naturally cause the scanned individuals to move more during acquisition than those without pathologies. Such movements can create artifacts and significantly affect the overall image quality. Furthermore, in DL image processing methods, it is important to assess how a given method works under different circumstances, such as those underlying processing of a pathological brain showing high levels of atrophy, as is the case for example for Alzheimer’s Disease (AD) patients. Therefore, it is worthwhile to assess the performance of different denoising methods in individuals with different pathologies. Our included datasets contain data from participants belonging to the cohorts AD, ADHD, AS, Bipolar, MCI, and CN. We assessed the performance of all the described methods within each diagnostic group. The results are summarized inFigure 3.

**Fig. 3.**
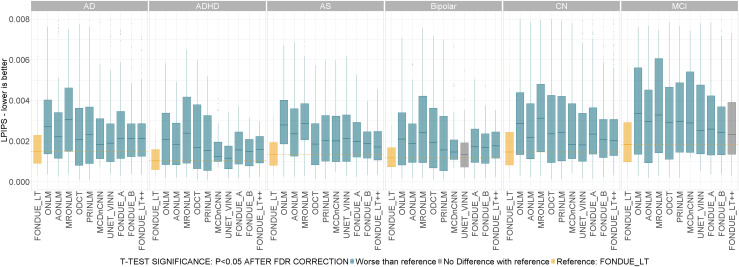
Performance of denoising methods (LPIPS) across diagnostic groups for stationary Rician noise between 1% and 9%. AD: Alzheimer’s Disease, ADHD: attention-deficit/hyperactivity disorder, AS: Autism Spectrum, Bipolar: Bipolar disorder, CN: Cognitively Normal, MCI: Mild Cognitive Impairment. t-Test color code: Yellow: reference method (FONDUE_LT). Blue: method significantly worse than reference. Red: method significantly better than reference. Gray: no significant differences found with respect to reference method. All the p-values in the sub-plots were corrected for multiple comparisons using the FDR method.

FONDUE_LT was consistently the best method across those tested. There were no significant differences between FONDUE_LT and UNET_VINN in the Bipolar cohort and between FONDUE_LT and FONDUE_LT++ in the MCI cohort. FONDUE_LT was significantly better than the rest of the methods in all other cases.

##### 3.1.3.2 Across scanner vendors

Performance of the methods was also evaluated for noise levels between 1% and 9% (stationary Rician noise) for the three different scanner vendors used: GE, Philips, and Siemens. The best denoising method across the three scanner vendors was FONDUE_LT, followed by AONLM and PRINLM, suggesting that the proposed method is robust with respect to scanner vendors (seeFig. 4).

**Fig. 4.**
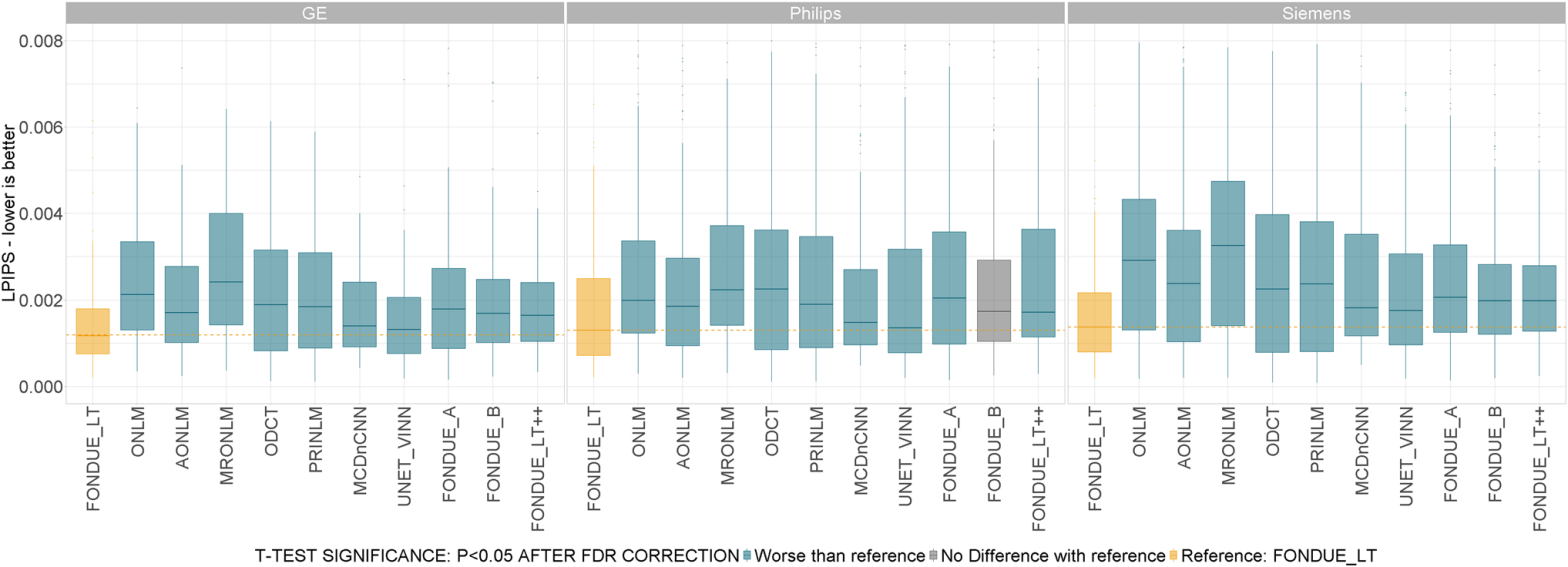
Denoising performance (LPIPS) for different scanner vendors. t-Test color code: Yellow: reference method (FONDUE_LT). Blue: method significantly worse than reference. Red: method significantly better than reference. Gray: no significant differences found with respect to reference method. All the p-values in the sub-plots were corrected for multiple comparisons using the FDR method.

##### 3.1.3.3 Across image resolutions

The performance of the methods was compared across three resolution categories: standard resolution (>=1 mm), high resolution (=0.7 mm), and super-high resolution (=0.5 mm). FONDUE_LT was the best method among those tested across all categories, being ODCT, PRINLM, and FONDUE_LT++ not significantly different than FONDUE_LT only for the super-high-resolution cohort (seeFig. 5).

**Fig. 5.**
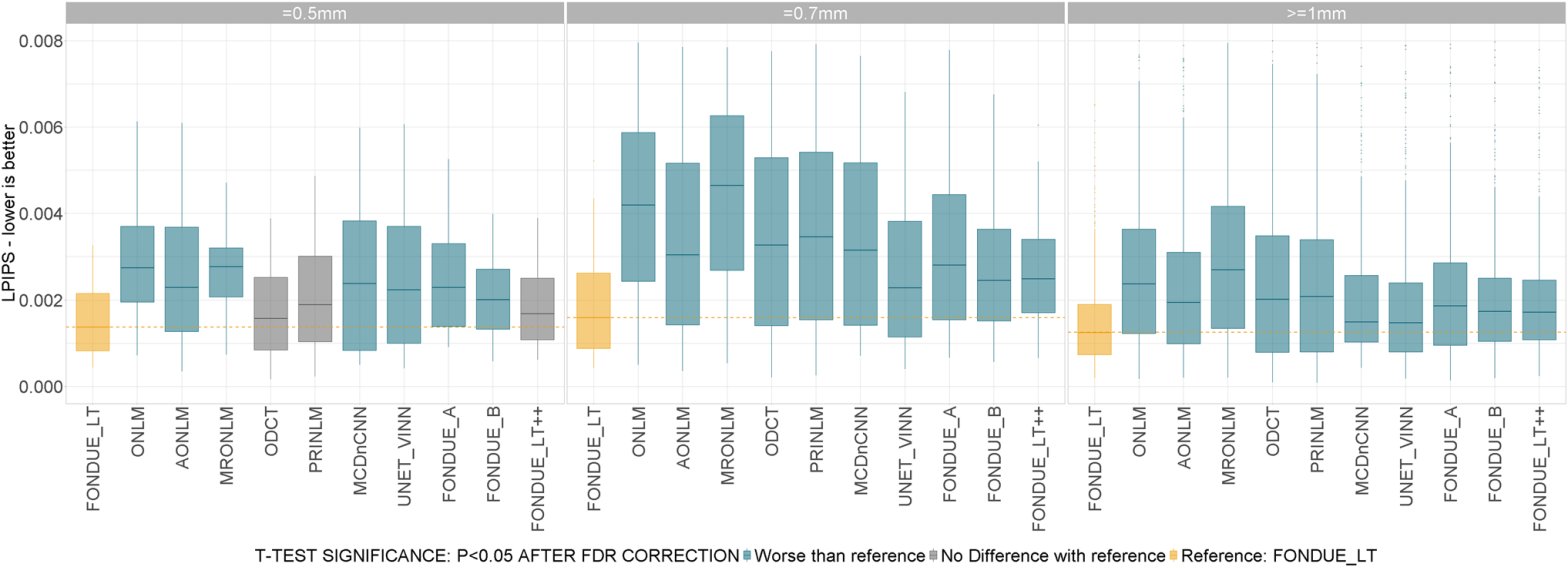
Denoising performance (LPIPS) for different resolutions. t-Test color code: Yellow: reference method (FONDUE_LT). Blue: method significantly worse than reference. Red: method significantly better than reference. Gray: no significant differences found with respect to reference method. All the p-values in the sub-plots were corrected for multiple comparisons using the FDR method.

##### 3.1.3.4 Across magnetic fields at 1 mm isotropic voxel size

This test was performed for the standard resolution (i.e., 1 mm^3^) which contained images with different field strengths (1.5T and 3T), resulting in FONDUE_LT being significantly better than the rest of methods across magnetic field strengths at 1 mm voxel sizes. The results are summarized inFigure 6.

**Fig. 6.**
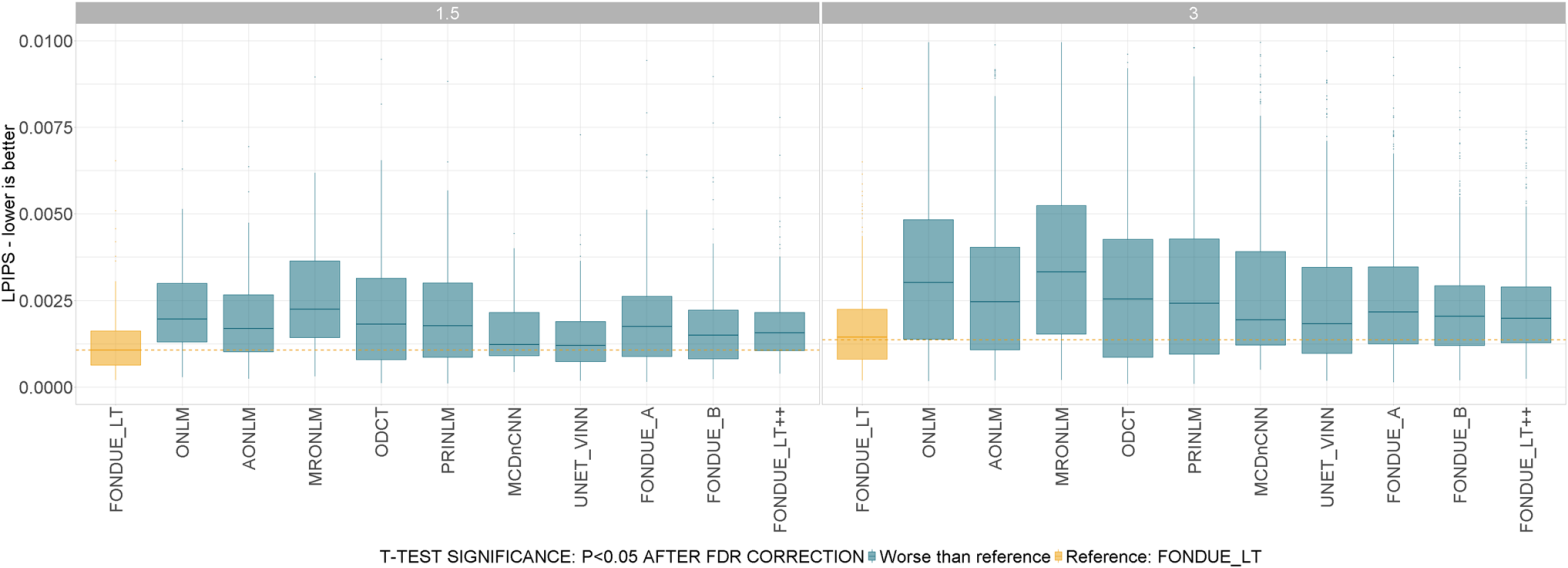
Denoising performance (LPIPS) for different magnetic field strengths (1.5T to 3T) at similar voxel sizes. t-Test color code: Yellow: reference method (FONDUE_LT). Blue: method significantly worse than reference. Red: method significantly better than reference. Gray: no significant differences found with respect to reference method. All the p-values in the sub-plots were corrected for multiple comparisons using the FDR method.

### 3.2 Denoising performance in natural noise (0% noise added raw images)

To assess the performance of the methods in a real-world scenario, that is, removing natural noise from raw images without artificial noise added, we used the same test set described inSection 3.1with no noise added or removed. The denoised images were compared against the surrogate ground truth produced with PRINLPCA.

No significant differences were observed between the tested methods except for HCP datasets where FONDUE_LT was significantly better than ONLM, AONLM, MRONLM, ODCT, PRINLM, MCDnCNN, and FONDUE_A for HCP, and for MIRIAD dataset, where FONDUE_LT was significantly better than ODCT, PRINLM, MCDnCNN, and FONDUE_LT++ (seeFig. 7).

**Fig. 7.**
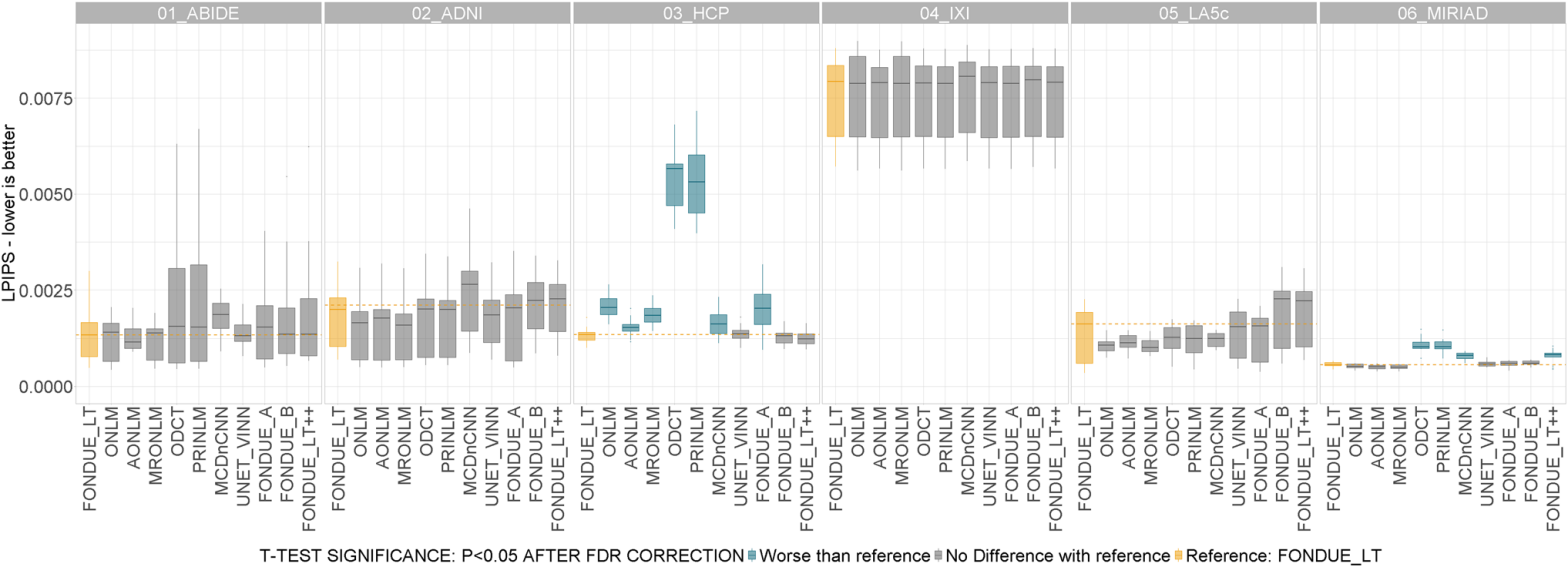
Denoising performance in terms of LPIPS on the six public datasets from the test set using the raw images (no artificial noise added). t-Test color code: Yellow: reference method (FONDUE_LT). Blue: method significantly worse than reference. Red: method significantly better than reference. Gray: no significant differences found with respect to reference method. All the p-values in the sub-plots were corrected for multiple comparisons using the FDR method.

### 3.3 CUSTOM_0.5_20rep dataset with a high-SNR ground truth

Table 2andSupplementary Table S15summarize the results of our experiments assessing the performance of FONDUE on a 0.5 mm^3^dataset (CUSTOM_0.5_20) with a high-SNR ground truth acquired by averaging 20 repetitions of the same image. Quality metrics indicate an increase in similarity with the reference image as the number of repetitions used for averaging increased. For this test, we used the AONLM-filtered version of the 20-repetition high-SNR image for each of the 2 reference images (ground truth). FONDUE_B2 was the best method for all metrics except for PSNR and AONLM. FONDUE_B2 was closely followed by FONDUE_B1, which also achieved good results overall.

**Table 2.** Comparison between denoising single repetition images with the different denoising methods versus averaging multiple acquisitions of the same image (rows 1 to 20) on CUSTOM_0.5_20rep dataset.

Type of technique	Image (number of repetitions averaged)	LPIPS	PSNR (dB)	SSIM	MSSSIM
AVERAGING	1_rep	0.0354	33.5487	0.9615	0.9909
	2_rep	0.0324	33.1320	0.9642	0.9907
	3_rep	0.0218	35.6632	0.9740	0.9940
	4_rep	0.0174	36.0417	0.9785	0.9948
	5_rep	0.0147	36.2304	0.9811	0.9953
	6_rep	0.0127	36.4700	0.9830	0.9956
	7_rep	0.0107	37.9666	0.9858	0.9966
	8_rep	0.0094	38.9519	0.9877	0.9971
	9_rep	0.0086	39.6785	0.9889	0.9974
	10_rep	0.0080	40.2552	0.9897	0.9977
	11_rep	0.0074	40.4186	0.9904	0.9978
	12_rep	0.0070	40.4951	0.9908	0.9978
	13_rep	0.0066	40.3229	0.9912	0.9978
	14_rep	0.0060	41.5080	0.9924	0.9982
	15_rep	0.0056	42.4974	0.9933	0.9985
	16_rep	0.0052	43.3886	0.9941	0.9987
	17_rep	0.0048	44.1294	0.9947	0.9989
	18_rep	0.0045	44.5770	0.9951	0.9990
	19_rep	0.0043	44.7420	0.9955	0.9991
	20_rep	0.0040	45.0122	0.9957	0.9991
NON-DL	AONLM	0.0297 ± 0.00022	21.9 ± 0.766	0.921 ± 0.000795	0.924 ± 0.00493
	MRONLM	**0.014** **±** **0.00101**	**27.3** **±** **0.939**	**0.971** **±** **0.00181**	**0.987** **±** **0.00169**
	ONLM	0.0152 ± 0.000754	27.2 ± 0.799	0.97 ± 0.00161	0.987 ± 0.00148
	ODCT	0.0288 ± 0.00149	24.7 ± 0.716	0.956 ± 0.00181	0.978 ± 0.0026
DL STAND ALONE	UNETVINN	0.00395 ± 0.000895	35.9 ± 1.85	0.985 ± 0.00122	0.996 ± 0.000668
	MCDNCNN	0.00932 ± 0.000826	30 ± 1.15	0.977 ± 0.0015	0.991 ± 0.00117
	FONDUE_A_BN	0.00399 ± 0.00086	34 ± 1.59	0.986 ± 0.00122	0.996 ± 0.000695
	FONDUE_A_NOBN	0.0054 ± 0.00119	34.6 ± 1.66	0.983 ± 0.00177	0.995 ± 0.000773
	FONDUE_B_BN	0.00538 ± 0.000893	33.7 ± 1.73	0.983 ± 0.00132	0.995 ± 0.000904
	FONDUE_B_NOBN	**0.00375** **±** **0.000954**	**36.9** **±** **1.99**	**0.986** **±** **0.00121**	**0.996** **±** **0.000652**
	FONDUE_LT	0.00809 ± 0.00111	30.1 ± 1.44	0.979 ± 0.00177	0.992 ± 0.0013
DL SECOND STAGE	FONDUE_LT_X2	0.00467 ± 0.00095	32.4 ± 2.07	0.984 ± 0.00132	0.994 ± 0.00119
	FONDUE_B1_BN	0.00434 ± 0.000829	33.2 ± 1.83	0.984 ± 0.00155	0.995 ± 0.00104
	FONDUE_B1_NOBN	0.0028 ± 0.000939	**38** **±** **2.15**	0.988 ± 0.00147	0.997 ± 0.000593
	FONDUE_B2_BN	0.00301 ± 0.00097	36.1 ± 1.96	**0.989** **±** **0.00111**	0.997 ± 0.000732
	FONDUE_B2_NOBN	**0.00257** **±** **0.000814**	37.9 ± 2.32	0.989 ± 0.00132	**0.997** **±** **0.000616**

In bold are shown the best methods for each metric for each type of technique, and underlined are the second best methods. Note: Reference image was the average of 20 repetitions with final filtering using AONLM. Results for subject 1 of the validation set. In the names of the DL methods, “A” indicates the use of VGG-16 loss, while the absence of “A” denotes that LPIPS loss was used for training. “_BN” indicates the inclusion of batch normalization, and “_NOBN” denotes its exclusion in the network architectures. If neither “_BN” nor “_NOBN” is indicated, batch normalization is not used.

Among the non-DL methods for both of our subjects, MRONLM was the best method across all the metrics, achieving similar performance than averaging ~5, ~1, ~4, and ~1 repetitions for LPIPS, PSNR, SSIM, and MS-SSIM, respectively, whereas for subject 2 (seeSupplementary Table S15), the best performing method was AONLM. For the standalone DL methods, the best performing method was FONDUE_B_NOBN, achieving performance comparable with averaging ~20, ~6, ~7, and ~7 repetitions in terms of LPIPS, PSNR, SSIM, and MS-SSIM. Finally, for the second stage DL methods, the best performing network was FONDUE_B2_NOBN, obtaining a performance comparable with averaging >20, ~7, ~10, and ~8 repetitions in terms of LPIPS, PSNR, SSIM, and MS-SSIM. It is worth noting that UNET-VINN was the second best performing network overall across the stand-alone DL methods, and that the second-stage DL methods improved the image quality over their stand-alone counterparts.

### 3.4 BrainWeb T1w phantom

Table 3compares the performance of all methods on BrainWeb (Collins et al., 1998) phantom data with different levels of stationary Rician noise. We have used the same custom PSNR and LPIPS Python functions as the ones used for the rest of tests. We conformed the volumes to be a cubic-shaped isotropic voxel sized ones, setting the foreground as those voxels with intensities larger than 10 (Manjón & Coupe, 2019) (in a scale of 0–255 using the same intensity rescaling explained onSection 2.8).

**Table 3.** Comparison of the methods tested on the BrainWeb phantom foreground at different levels of stationary Rician noise.

	1%	3%	5%	7%	9%
FONDUE_A_BN	39.6648 | 0.009	39.6596 | 0.0085	37.393 | 0.0091	37.112 | 0.0111	35.4759 | 0.014
FONDUE_A_NOBN	48.548 | 0.001	43.3743 | 0.0044	40.0836 | 0.0078	37.5498 | 0.0112	35.4133 | 0.0151
FONDUE_B_BN	44.44 | 0.0044	42.183 | 0.005	39.5584 | 0.008	37.2521 | 0.0124	35.3599 | 0.0175
FONDUE_B_NOBN	45.2334 | 0.0032	41.6986 | 0.005	38.5112 | 0.0074	36.2074 | 0.0095	34.2529 | 0.012
FONDUE_B1_BN	44.9437 | 0.0041	42.6502 | 0.0057	40.0726 | 0.0076	37.7945 | 0.01	35.8581 | 0.014
FONDUE_B1_NOBN	44.7503 | 0.0039	41.2764 | 0.0066	38.2736 | 0.009	36.0277 | 0.0111	34.0937 | 0.0132
FONDUE_B2_BN	41.6465 | 0.0086	40.6448 | 0.0092	39.0628 | 0.0088	37.2529 | 0.0101	36.194 | 0.0109
FONDUE_B2_NOBN	43.9822 | 0.0049	41.0628 | 0.0074	38.2083 | 0.0096	36.185 | 0.0116	34.4674 | 0.0137
FONDUE_LT	48.9675 | 0.0015	43.8014 | 0.0029	40.4605 | 0.0061	37.9064 | 0.0107	35.91 | 0.0156
FONDUE_LT_X2	46.1706 | 0.0039	43.1584 | 0.005	40.6826 | 0.0058	38.4267 | 0.007	36.4394 | 0.0088
MCDnCNN	43.1976 | 0.0111	41.2521 | 0.0106	38.8612 | 0.0132	36.6912 | 0.0188	34.7923 | 0.0264
UNET_VINN	43.3911 | 0.0042	39.4887 | 0.0076	37.7009 | 0.0124	35.4727 | 0.0192	33.8765 | 0.0269
PRINLPCA	51.4312 | 5e-04	45.7557 | 0.0021	42.4955 | 0.0042	39.8795 | 0.0062	37.7257 | 0.0081

Results displayed as “PSNR | LPIPS.” In the names of the DL methods, “A” indicates the use of VGG-16 loss, while the absence of “A” denotes that LPIPS loss was used for training. “_BN” indicates the inclusion of batch normalization, and “_NOBN” denotes its exclusion in the network architectures. If neither “_BN” nor “_NOBN” is indicated, batch normalization is not used.

As can be noticed, PRINLPCA is superior in this use case, followed by FONDUE_LT and FONDUE_A (non-BN).

### 3.5 Multi-resolution performance: UH_250, UH_500, and UH_1000 datasets

We used the UH_NNN dataset to perform qualitative visual assessments of the reported denoising methods at multiple resolutions (seeSupplementary Table S1and Section A inSupplementary Materials). UH_NNN consists of a 7T brain MRI of the same individual scanned at three different isotropic resolutions: 0.25 mm^3^(UH_250), 0.5 mm^3^(UH_500), and 1.0 mm^3^(UH_1000) (Lüsebrink et al., 2017,^2018^).

#### 3.5.1 Visual assessment results: Stand-alone networks

For UH_250 dataset, the inter-rater agreement was excellent (88.9%), indicating FONDUE_B_NOBN as the best denoised image, followed by FONDUE_A_BN and UNET_VINN (both with inter-rater agreement of 66.7%). For UH_500, inter-rater agreement on the best denoised image was poor (11.1%), but overall, FONDUE_A_BN and FONDUE_B_NOBN were both ranked as best by either rater in 77.8% of the cases, followed by FONDUE_B_BN. For UH_1000, there was no agreement between the raters on the best denoised image (0%), however, FONDUE_LT was ranked as best by both raters, followed by FONDUE_B_NOBN.Supplementary Table S7summarizes the results of these visual assessments.

#### 3.5.2 Visual assessment results: Two-stage networks

For the UH_250 dataset, FONDUE_B2_NOBN was the best method (voted as best by either rater in 88.9% of the cases), followed by FONDUE_B2_BN and FONDUE_B1_NOBN. For UH_500, FONDUE_LT_X2, FONDUE_B1_BN, and FONDUE_B2_NOBN were the top three methods, followed by FONDUE_B1_NOBN and FONDUE_B2_BN. Finally, for UH_1000, FONDUE_B1_NOBN was the best method (voted as best by either rater in 50% of the cases), followed by FONDUE_B2_BN and FONDUE_B2_NOBN.Supplementary Table S8summarizes the results of these visual assessments.

### 3.6 UH_250 dataset: Quantitative evaluation

Eight raw acquisitions were available from the UH_250 dataset which were rigidly co-registered at 0.3 mm^3^isotropic voxel size resolution. Following co-registration, the eight aligned images were averaged, and this average image was denoised using PRI-NLPCA to generate a ground-truth surrogate. Each of the eight co-registered raw acquisitions was then denoised using both AONLM and FONDUE (A, B, B1, and B2, and LT). Similarity metrics were computed using the ground-truth surrogate as a reference (seeTable 4). FONDUE_B1 outperformed other FONDUE versions as well as AONLM across all metrics, except for PSNR. Other methods (i.e., ONLM, MRONLM, PRINLM, ODCT) were unable to successfully process UH-250 images (seeSection 4).

**Table 4.** Denoising performance comparison using a 0.3 mm^3^dataset versus ground truth.

Type	Name	LPIPS	PSNR	SSIM	MS-SSIM
Non-DL	AONLM	0.0277 ± 0.00975	27.7 ± 2.97	0.961 ± 0.0116	0.977 ± 0.00884
Stand-alone DL	FONDUE_A_BN	0.0163 ± 0.00211	27.5 ± 1.33	0.985 ± 0.00188	0.986 ± 0.00269
	FONDUE_A_NOBN	0.0172 ± 0.00236	27.5 ± 1.28	0.984 ± 0.00237	0.985 ± 0.00271
	FONDUE_B_BN	0.0169 ± 0.00203	27.5 ± 1.33	0.985 ± 0.00192	0.985 ± 0.00272
	FONDUE_B_NOBN	**0.0145** **±** **0.00245**	**29.3** **±** **1.71**	**0.988** **±** **0.00259**	**0.989** **±** **0.00307**
	FONDUE_LT	0.0164 ± 0.0022	27.8 ± 1.39	0.986 ± 0.0022	0.986 ± 0.00279
	MCDnCNN	0.0172 ± 0.00194	27.7 ± 1.37	0.984 ± 0.00182	0.986 ± 0.0027
	UNET_VINN	0.0172 ± 0.00207	27.7 ± 1.33	0.985 ± 0.002	0.986 ± 0.00271
Second-stage DL	FONDUE_B1_NOBN	**0.014** **±** **0.00239**	**29.7** **±** **1.66**	**0.989** **±** **0.00257**	**0.989** **±** **0.00296**
	FONDUE_B2_BN	0.0162 ± 0.00224	27.5 ± 1.33	0.986 ± 0.00219	0.986 ± 0.00279
	FONDUE_B2_NOBN	0.0144 ± 0.00231	29.7 ± 1.63	0.989 ± 0.00257	0.989 ± 0.00293
	FONDUE_LT_X2	0.0163 ± 0.00223	27.8 ± 1.39	0.986 ± 0.00222	0.986 ± 0.00278

In bold and underlined are indicated the best and second best performing methods for each type of network, respectively. In the names of the DL methods, “A” indicates the use of VGG-16 loss, while the absence of “A” denotes that LPIPS loss was used for training. “_BN” indicates the inclusion of batch normalization, and “_NOBN” denotes its exclusion in the network architectures. If neither “_BN” nor “_NOBN” is indicated, batch normalization is not used.

### 3.7 Effect of denoising on downstream full-brain segmentation

To assess the extent to which denoising techniques can impact downstream results, we used FastSurferVINN to perform brain segmentation, using images that were denoised using the previously discussed methods for the HCP images on our test set. Then, we merged the masks into five main regions (see Section D.3 inSupplementary Materials). We then computed both Dice-Kappa and Intersection-Over-Union (IOU) metrics, comparing the segmentation masks obtained based on the ground-truth images against those obtained from applying different denoising methods on data with different noise types and levels. After averaging the mean segmentation metrics (Dice/IOU) of the five generated regions across all datasets, we obtained that for either segmentation metric (Dice or IOU), MRONLM was significantly the best method for 1% (stationary and variable) noise levels followed by ONLM in second place. For 3% (both stationary and variable) the best method was PRINLM followed by FONDUE_LT, and for the rest of the noise levels, FONDUE_LT was significantly the best method (seeFig. 8).

**Fig. 8.**
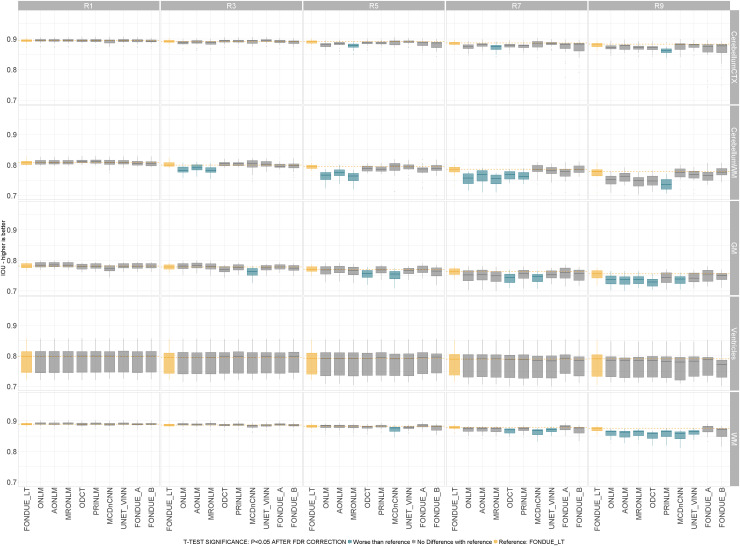
IOU coefficient of the five different brain regions over 1–9% of stationary Rician noise.

### 3.8 Processing times

Table 5shows the processing times for the BrainWeb (Collins et al., 1998) phantom and UH_500 on the described system. The BrainWeb volume contained181×217×181 voxels (1 mm^3^isotropic voxels) and UH_500 had352×416×416voxels (0.5 mm^3^isotropic).Table 5shows both CPU and GPU (CUDA) results for FONDUE to provide a fair look into the processing times. Note that the time reported for FONDUE refers to FONDUE_A and FONDUE_B. FONDUE_B1 and FONDUE_B2 take twice the time reported in this table since it requires two runs of a network with the same number of parameters. All tests were conducted using automatic mixed precision and a batch size of 1.

**Table 5.** Processing time comparison of all the denoising methods in this study using a single sample of BrainWeb and UH_500 datasets.

Dataset	Device	ONLM	AONLM	MRONLM	ODCT	PRINLM	PRINLPCA	FONDUE – Stand-alone	FONDUE – Stand-alone + refinement	FONDUE_LT++
BrainWeb	CPU	76.99s	207.32s	179.91s	14.66s	19.82s	646.6s	792.6s	~1580s	N/A
	GPU	N/A	N/A	N/A	N/A	N/A	N/A	36.1s	~78s	48.7s
UH_500	CPU	673s	3317s	2826s	66.3s	143.3s	~13900s	~4300s	~8500s	N/A
	GPU	N/A	N/A	N/A	N/A	N/A	N/A	136s	~280s	196.1s

It is worth noting as well that FONDUE processing times reported inTable 5include the entire pipeline, including reading the image, preprocessing, denoising in the three anatomical planes (view aggregation), and writing of the final file into the device storage unit. Furthermore, the two-stage FONDUE networks take about twice the time as the FONDUE stand-alone networks since it implies running a similar network twice. To enable a fair comparison, all reported times include reading and writing the files to the disk. FONDUE_LT++ took 48.7s for BrainWeb images and 206s for the UH_500 dataset on the GPU, as opposed to the lighter standard FONDUE versions which took 36.1s and 136s, respectively. Furthermore, in terms of VRAM usage during inference, FONDUE_LT++ took around 12GB and up to 16GB for BrainWeb and UH_500 datasets, respectively, whereas standard FONDUE versions took 1GB for BrainWeb and 2.3GB for UH_500 datasets. FONDUE_LT++ took around 12.6s and 60.1s longer to process the images than FONDUE_LT.

## 4 Discussion

In this paper, we have proposed FONDUE, a human brain structural MRI denoising method capable of handling multiple resolutions, scanner models, and field strengths, even outside the resolutions for which it was trained. The proposed method outperformed or matched the state-of-the-art methods when compared with reference surrogates (silver standard). The usage of the deep perceptual loss functions resulted in a good balance between denoising and detail preservation.

Most denoising techniques use MAE—also called L1—(Gurrola-Ramos et al., 2021;Tripathi & Bag, 2020), and MSE—also called L2—as loss functions (Jiang et al., 2018;Manjón & Coupe, 2018;Tripathi & Bag, 2020). However, L1 and especially L2 loss functions can produce over-smoothing in the output images when used alone (Mehdizadeh et al., 2021). To address over-smoothing, some have combined traditional L1 or L2 functions with structural similarity metrics such as SSIM and MS-SSIM (Augustin et al., 2022). Even though this improves the results, over-smoothing is still present, potentially leading to the loss of information in high-frequency regions (Mehdizadeh et al., 2021). LPIPS is a similarity metric that makes use of already trained classification neural networks that were trained with naturalistic images, comparing each of the filters at every depth of the neural network using L2 (R. Zhang et al., 2018). Then, they fine tuned the weights of the network using a human judgment-based loss so that the metric corresponds better to behavior of human vision. This approach produces a similarity metric that is closer to human judgment and is more sensitive to blurriness that might perceptually alter the image but produce seemingly high MAE values (R. Zhang et al., 2018). LPIPS can detect features such as edges and textures, and as a result, it can compare each of these features at a deeper level than just comparing two images without being filtered by a network. These characteristics made LPIPS a logical avenue to explore for our application of interest.

InMehdizadeh et al. (2021), the authors explored training a denoising CNN using different loss functions, including L1, L2, LPIPS, and L_feat_, as well as different combinations/variations of these loss functions. They showed that when using deep perceptual losses such as LPIPS and L_feat_, the produced denoised image was significantly sharper and less blurry. Additionally, they showed that LPIPS and L_feat_(using all the depths of the feature extraction networks) produced the sharpest images, but the denoised output appeared still slightly noisy on image regions that are expected to be smooth. Moreover, when they used only the first layer of convolutions from L_feat_, the image was sharper than with L1/L2, and the image regions expected to be smooth were smooth. Finally, they showed that pixel-wise losses—for example, L1—produced images with high performance in terms of PSNR but were notably blurry.

The selection of loss function for the second-stage networks (FONDUE_B1 and FONDUE_B2) was influenced by the following facts: (i) FONDUE_A (L_feat_loss) applied to the output of FONDUE_B (LPIPS loss) produced clean images with fine details preserved, while the inverse process produced images practically similar to the output of FONDUE_A with considerably fewer details; (ii) FONDUE_B applied to the output of FONDUE_B in a “recursive” manner did not produce a cleaner image, keeping the noisy appearance on the low-frequency regions; and (iii) we also trained a refinement FONDUE network using LPIPS and FONDUE_B as first denoising step, but the “refinement” was not even able to retain the performance of the first step, instead reducing image quality. We, therefore, concluded that a two-step approach would be optimal. First, using a network trained with the LPIPS loss function to denoise and sharpen the image, especially in highly detailed regions. Second, post-processing with a network trained with the first convolutional layer of L_feat_to preserve fine details and remove residual noise.

The training time was ~6 h per epoch for FONDUE_A and FONDUE_B, and ~2.5 h per epoch for FONDUE_B1 and FONDUE_B2 using a batch size of 2, turning the proposed method into an affordable one. Total training time was less than 3 days on the—still domestic grade—NVIDIA RTX 3090, thanks to the Super Convergence strategy. Including 0% noise levels in training was crucial for obtaining optimal results since the network learned how to not degrade noise-free images, which is a potential issue when not considering training using noise-free images. Similarly, the inclusion of automatic mixed precision (AMP) during the training made it feasible to denoise images with varying resolutions—including ultra-high-resolution images from the UH_250 dataset—using an entry-level NVIDIA RTX 3060 GPU, since AMP uses approximately half the GPU memory and reduces the computational cost without significantly affecting performance.

We further report on the unreliability of using solely PSNR as a quality metric, as it did not monotonically increase as more repetitions were considered for averaging on the CUSTOM_0.5 dataset (seeTable 2). More complex metrics such as LPIPS, SSIM, and MS-SSIM monotonically improved as more repetitions were included for the high-SNR average image. Therefore, our observations agree with the recent literature on the need for using similarity metrics that can be more similar to human visual perception (Andersson et al., 2020;Hore & Ziou, 2010;R. Zhang et al., 2018).

As opposed to the traditional approaches that use UNet/UNet++—like architectures (Gurrola-Ramos et al., 2021;Zhou et al., 2018) in which filter number increases while going deeper into the encoder part of the network, our approach keeps low to very low GPU memory usage by keeping the same number of filters throughout the network. Furthermore, our experiments showed that not all the sub-candidates “YN” produced by the sub-networks should have the same weight when averaging to produce the final result (seeSupplementary Table S4).

Our design choice in which we trained FONDUE_A and FONDUE_B for both non-BN and BN helped us investigate the effect of using BN inside the networks for both networks. It can be clearly noted in Section C inSupplementary Materialsthat non-BN networks produced a better result than the BN counterparts, matching the observation from the most recent literature on image-to-image CNNs (Manjón & Coupe, 2019;Rombach et al., 2022;X. Wang et al., 2018). Furthermore, removing the BN layer also helped the network to generalize better to very high-resolution images (even beyond the resolutions used in our training set), producing in general cleaner and sharper images. The exception to the observation in which removing BN produced a worse outcome than the BN counterpart is for FONDUE_A using voxel sizes of 0.5 mm or smaller. The source of this shifting in performance should be further investigated with a larger sample size.

When comparing FONDUE_A and FONDUE_B in terms of LPIPS, FONDUE_B worked generally better, producing better results than those from FONDUE_A, except when low noise levels and/or standard resolutions are being used. In contrast, FONDUE_A obtained better overall performance in terms of the other three metrics tested (PSNR, SSIM, and MSSSIM). This is no surprise since FONDUE_B was trained using LPIPS as a loss function. However, performance of FONDUE_B was more stable across noise levels and resolutions. Furthermore, when dealing with volumes whose voxel size is equal to or smaller than 0.5 mm isotropic, FONDUE_B performed the best across all the DL stand-alone methods tested.

Visual assessments identified FONDUE_B_NOBN and FONDUE_B2_NOBN as the best stand-alone and two-stage networks, respectively (Sections 3.5.1 and 3.5.2). These findings aligned with our quantitative assessments using a similar resolution (Section 3.6;Table 4).

Overall, UNET-VINN performed well compared with FONDUE_A and FONDUE_B, frequently outperforming MCDnCNN. The inclusion of the normalization layer VINN is likely the main source of generalization capabilities across resolutions, and the semantic compression occurring in this type of architecture compared with the one from MCDnCNN suggests that the deeper—more abstract—layers contribute to the generalization capabilities of the network. This is also supported by the values of the λ values, which were optimized during the training and resulted in a large contribution from the deeper layers in all FONDUE networks.

The use of a second stage network such as FONDUE_B1 or FONDUE_B2 did not improve the performance of the baseline network FONDUE_B for standard or high resolutions.

FONDUE_LT showed an improvement in performance compared with FONDUE_B, producing cleaner and sharper results in standard or high resolutions. However, when very high resolutions with high levels of noise were found, then the performance of the network gets reduced. The output of the images in these scenarios look over sharpened. In these cases, FONDUE_B (and the refinement method FONDUE_B1) is the best method, outperforming AONLM across all the metrics. Other methods (ONLM, MRONLM, PRINLM, ODCT) failed when processing images of this resolution. More specifically, the “noise estimation” function (a common pre-processing step in all four methods) returned a failure message stating that noise estimation failed.

Even if the lambda values are not similar across the networks, the highest value was always found on λ4, λ5, or λ6, and the smallest values were found on λ1, λ2, or λ3 (with exception of FONDUE_A_NOBN in which the smallest value was λ4). This means that the optimal weighted average has a considerable contribution of the deepest—more abstract—layers. Notably, there is a noticeable change in these values when comparing FONDUE_B_NOBN and FONDUE_LT: λ5 becomes the highest weight value while the rest of the values decrease when increasing the number of iterations fivefold.

However, our study is not without limitations. FONDUE showed little gain in performance compared with ODCT and PRINLM when 1% and 3% noise was present in low-resolution datasets. This indicates that these non-DL algorithms are especially better for low-noise and low-resolution settings. However, FONDUE did denoise these images as well as other state-of-the-art methods being FONDUE_LT the best runner-up against the non-DL methods. Furthermore, in ultra-high-resolution MRIs contaminated with very large amounts of noise (e.g., raw 0.5 mm^3^MRIs inSection 3.5), FONDUE_LT was outperformed by other methods. This drop in performance might result from overfitting, as FONDUE_LT attempts to keep as many high-frequency anatomical details as possible, and with this specific sequence, noise level, and resolution, the network confounds noise with fine anatomical details. Further strategies can be followed to ameliorate this, such as increasing the number of training images at 0.5 mm^3^, adding dropout to the network, training the network in a GAN approach, and adding more types of latent augmentations.

Based on the presented results, our recommendation on which method to use is summarized inFigure 9:

**Fig. 9.**
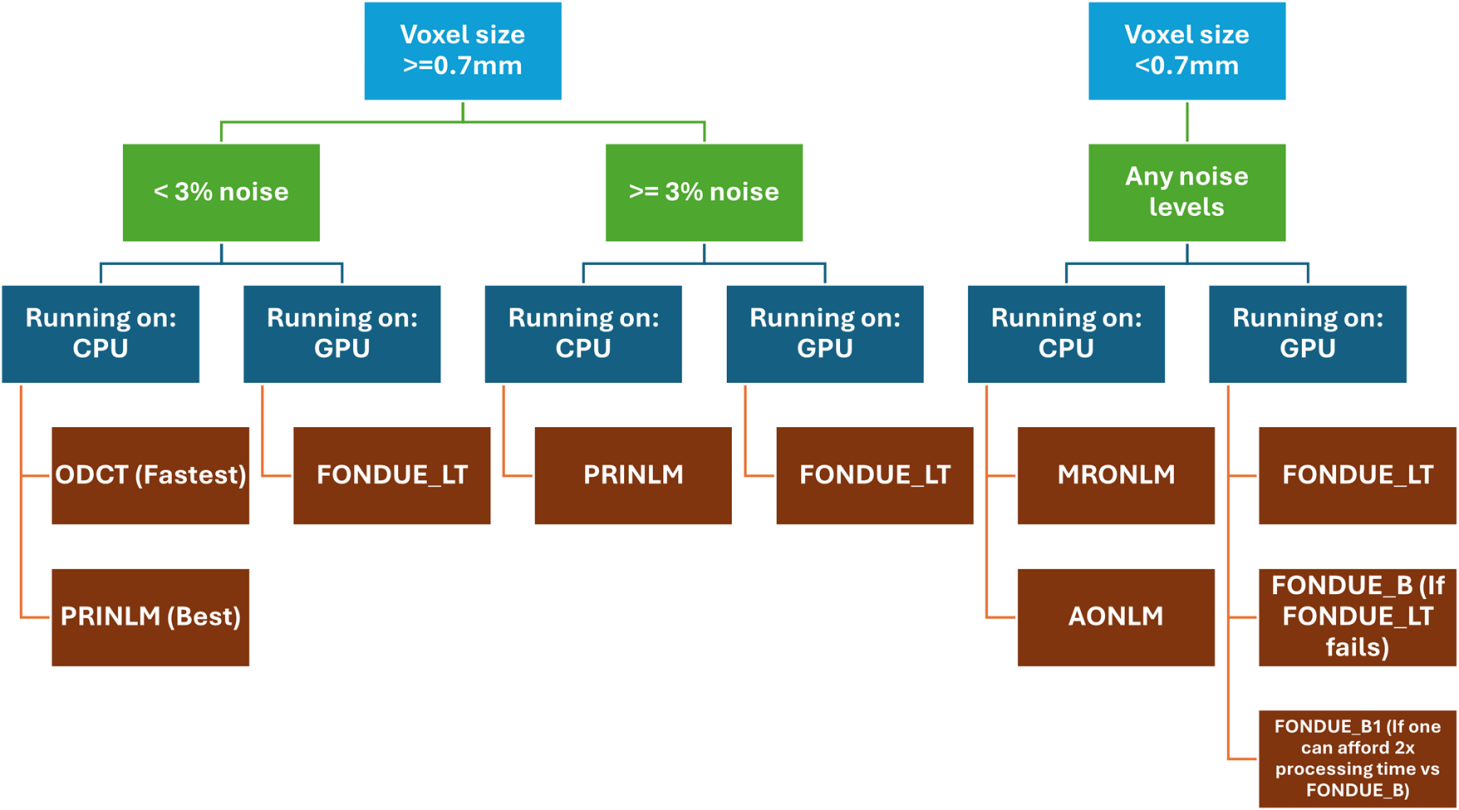
Election of denoising method based on the results of our study. Light blue: input image isotropic voxel size. Green: noise level. Navy blue: processing device. Brown: method of election.

Future work will include training using other perceptual loss functions such as FLIP (Andersson et al., 2020), training specific weights for each anatomical plane instead of using axial weights for all three planes, validating FONDUE on other MRI modalities such as T2w, T2*, FLAIR, and PD, and training a 2D-only version of FONDUE that might be better suited for usage with non-isotropic images.

## Supplementary Material

Supplementary Material

## Data Availability

All the datasets used in this study (except for UH_500 dataset) are publicly available. A detailed list of the URLs to the used datasets is provided onSupplementary Table S3. The code and trained weights are made publicly available onhttps://github.com/waadgo/FONDUE.
