# Supplementary material for: FONDUE: Robust resolution-invariant denoising of MR images using Nested UNets
Source: Imaging Neurosci (Camb). 2024 Nov 21;2:imag-2-00374. doi: 10.1162/imag_a_00374 (PMC12315758; doi:10.1162/imag_a_00374)
Supplement: Supplementary Material [file imag_a_00374-supp.pdf]

Supplementary Materials

A. Datasets description

ABIDE-II: The Autism Brain Imaging Data Exchange II (Di Martino et al., 2017) includes MRIs from individuals with autism spectrum disorder as well as matched controls. Study sites used either 3D magnetization-prepared rapid gradient echo (MPRAGE) sequences or vendor-specific custom variants. While 3T images from GE, Philips, and Siemens vendors were available in this dataset, images acquired with a Philips Achieva scanner will be used for this project in order to balance the number of samples from each vendor and to have native images of varying resolutions, mostly non-isotropic, varying from over to sub-millimeter.

ADNI-1: The Alzheimer's Disease Neuroimaging Initiative 1 (Mueller et al., 2005) includes data from normal aging individuals as well as people with subjective cognitive decline, mild cognitive impairment, and Alzheimer's dementia such as structural and functional MRI, positron-emission tomography (PET), as well as biological biomarkers and clinical assessments; it was collected across 59 study sites and includes 1.5T and 3T MRIs from different vendors. From the ADNI-1, we included MRI scans from GE, Siemens, and Philips scanners with a voxel resolution of 1.0x1.0x1.2mm^3. Due to the numerous testing sites that acquired MRIs for ADNI-1, the acquisition parameters vary from site to site, providing us with a variety of parameter ranges presented in Table 1.

HCP: The Human Connectome Project Young Adult study (Van Essen et al., 2012), is a dataset comprised of brain images from 1200 healthy subjects ranging from 22 to 35 years old acquired using a 3T Siemens scanner with a sub-millimeter voxel size (isotropic 0.7mm^3).

IXI: The IXI Dataset (*IXI Dataset*, n.d.) includes MRI scans from 600 normal healthy subjects from three different hospitals in London, UK. Phillips 3T and 1.5T systems were used at Hammersmith and Guy's Hospitals, respectively.

LA5c: The UCLA Consortium for Neuropsychiatric Phenomics LA5c Study (Gorgolewski et al., 2017) includes scans from 130 healthy controls, 43 individuals with diagnoses of adult Attention-Deficit Hyperactivity Disorder (ADHD), 49 individuals with bipolar disorder, and 50 individuals with schizophrenia. Neuroimaging data were acquired on a Siemens 3T scanner using an MPRAGE sequence.

MIRIAD: The Minimal Interval Resonance Imaging in Alzheimer's Disease (Malone et al., 2013) is a longitudinal study containing T1 MRI scans of 46 patients with Alzheimer's disease at

intervals of 2, 6, 14, 26, 38, and 52 weeks, 18 and 24 months from baseline. The images were acquired with a GE 1.5T scanner using an inversion recovery prepared fast spoiled gradient recall sequence.

Custom_0.5: A custom dataset of 21 individuals ranging from 25 to 50 years old (11 females) acquired at the Montreal General Hospital (McGill University Research Center) was also used as part of the training and validation corpus. We used images from 16 out of the 21 individuals that passed quality control. The dataset was acquired using a 3T Siemens Prisma scanner with a Multi-Echo MPRAGE (MEMPRAGE) sequence using a 64-channel head coil and online GRAPPA acceleration. Importantly, 14 out of the 16 individuals in CUSTOM_0.5 were scanned 6 times, allowing us to generate higher-quality average ground truth images. These individuals formed the dataset named CUSTOM_0.5_6rep, which was further divided for training and testing during training. A subset of 2 individuals (which was left out of the training set) from CUSTOM_0.5_6rep was further scanned another 14 times, generating a total of 20 scans for each of these 2 individuals, referred to as the CUSTOM_0.5_20rep dataset. CUSTOM_0.5_20rep subjects were scanned 20 times in 3 sessions separated by a few months. 6 scans were acquired in the first session and 7 scans in the next two sessions. The objective was to obtain the cleanest possible MRIs for each individual after averaging their respective 20 available scans to be used as the gold standard for validating FONDUE.

UH_NNN: A novel dataset by (Lüsebrink et al., 2017) known for being the highest-resolution in-vivo structural MRI dataset available to date. UH-NNN consists of a single-subject dataset acquired with a 7T Siemens scanner at 0.25 mm^3 isotropic voxel size. They collected 8 acquisitions at this resolution, using motion tracking correction, linear and non-linear co-registration using ANTs, non-homogeneity correction and slight denoising. They provide images of the same subject at three different resolutions: 0.25 mm^3 isotropic (UH_250), 0.5 mm^3 isotropic (UH_500) and 1.0 mm^3 isotropic (UH_1000), with the acquisition parameters summarized in Table 1. We did not use any of these images for training and performed qualitative validation of the performance of FONDUE in denoising a single repetition of the raw 7T images at three different resolutions. We also quantitatively compared the performance of FONDUE against a commonly used NDL method in denoising a single repetition of UH_250. To generate the ground truth for these comparisons, we co-registered the 8 repetitions (rigid followed by nonlinear registration as was done in the original article, rescaled to 0.3 mm^3 isotropic voxel size).

B. Training insights

Table S.1: *Summary of the datasets used to train and validate FONDUE.*

Study	Cohort(s)	Training FONDUE A & B [N]	Training FONDUE B1 & B2 [N]	Validation sample all NETWORKS [N]	Testing sample [N]
ABIDE-II	AS	169	50	14	20
ADNI1	HA, MCI, AD	131	50	11	20
HCP	HA	174	50	15	20
IXI	HA	407	50	25	20
LA5c	HA, SZ, ADHD, Bipolar	89	50	16	20
MIRIAD	AD	48	48	7	14
Custom_0.5	HA	12	12	3	3
UH_250	HA	0	0	0	1
UH_500	HA	0	0	0	1
UH_1000	HA	0	0	0	1
Acronyms: AS = Autism Spectrum, HA = Healthy Adults, MCI = Mild Cognitive Impairment, AD = Alzheimer's Disease, ADHD = Attention-Deficit / Hyperactivity Disorder, SZ = Schizophrenia, Bipolar = Bipolar Disorder<br>[N] = Sample size by number of subjects.					

Table S.2: *Summary of the characteristics of each FONDUE variant*

FONDUE version	Batch-normalized?	Loss function	Noise added during Training	Trained epochs	Training sample size	Stand alone?
A_BN	Yes	L_{feat}	0-9% variable Rician	10	1030	Yes
A_noBN	No	L_{feat}	0-9% variable Rician	10	1030	Yes
B_BN	Yes	LPIPS	0-9% variable Rician	10	1030	Yes
B_noBN	No	LPIPS	0-9% variable Rician	10	1030	Yes
LT	No	LPIPS	0-9% variable Rician	50	1030	Yes
LT++	No	LPIPS	0-9% variable Rician	50	1030	Yes
LT_X2	No	LPIPS	0-9% variable Rician	50	1030	No: needs FONDUE_LT
B1_BN	Yes	LPIPS	0-4% variable Rician	10	91	No: needs FONDUE_B_BN
B1_noBN	No	LPIPS	0-4% variable Rician	10	91	No: needs FONDUE_B_noBN
B2_BN	Yes	LPIPS	4-9% variable Rician	10	91	No: needs FONDUE_B_BN
B2_noBN	No	LPIPS	4-9% variable Rician	10	91	No: needs FONDUE_B_noBN

Table S.3: *URLs of all the public datasets used on FONDUE training and validation*

Dataset	URL
ABIDE-II	http://fcon_1000.projects.nitrc.org/indi/abide/databases.html
ADNI1	https://ida.loni.usc.edu/login.jsp?project=ADNI
HCP	https://www.humanconnectome.org/study/hcp-young-adult/document/1200-subjects-data-release
IXI	https://brain-development.org/ixi-dataset/
LA5c	https://openneuro.org/datasets/ds000030/versions/00016
MIRIAD	https://www.ucl.ac.uk/drc/research/research-methods/minimal-interval-resonance-imaging-alzheimers-disease-miriad
UH-NNN	https://openneuro.org/datasets/ds003563/versions/1.0.1
BrainWeb	https://brainweb.bic.mni.mcgill.ca/brainweb/selection_normal.html

Table S.4: *Obtained values for λ_N during training for all the FONDUE stand-alone networks*

	FONDUE_A_BN	FONDUE_A_NOBN	FONDUE_B_BN	FONDUE_B_NOBN	FONDUE_LT
$\lambda 1$	0.0360	0.1345	0.0598	0.0730	0.0183
$\lambda 2$	0.0945	0.1022	0.0818	0.0714	0.0149
$\lambda 3$	0.0149	0.2434	0.0412	0.1553	0.0442
$\lambda 4$	0.1987	0.0382	0.2004	0.1780	0.1326
$\lambda 5$	0.2006	0.1312	0.1649	0.1561	0.2127
$\lambda 6$	0.1351	0.0703	0.1557	0.1325	0.0417

C. Ablation studies

1. Prototype networks: to batch normalize or not?

As mentioned in the methods section, we assessed FONDUE_A and FONDUE_B with and without Batch Normalization. Table S.5 summarizes these results, comparing the batch-normalized networks against their non-batch normalized counterparts using paired t-tests and correcting for FDR. This table depict the number of cases where a given method was superior to others, across the 6 datasets and 10 noise types (1, 3, 5, 7, and 9%, both variable and stationary; i.e. the number of times a given method was significantly the best out of 60. The parentheses indicate the percentage of times that a given method was significantly the best.

Table S.5: *Ablation study. Comparing different network designs using T-tests with FDR correction. The results are presented as "N (X%)", where N is the times that for a given test (first column), a method (second column) was significantly better than the rest for a given metric. X represents in percentage the number of times that a given method was significantly superior to the rest for a given metric.*

FONDUE versions compared (test)	Method	Metric			
		LPIPS	MS-SSIM	PSNR	SSIM
A_BN vs A_NOBN (section C.1)	FONDUE_A_BN	10 (23%)	2 (5%)	4 (8%)	2 (4%)
	FONDUE_A_NOBN	33 (77%)	41 (95%)	44 (92%)	48 (96%)
B_BN vs B_NOBN (section C.1)	FONDUE_B_BN	21 (47%)	11 (41%)	11 (38%)	0 (0%)
	FONDUE_B_NOBN	24 (53%)	16 (59%)	18 (62%)	42 (100%)
A_NOBN vs B_NOBN (section C.2)	FONDUE_A_NOBN	8 (19%)	37 (95%)	45 (100%)	39 (91%)
	FONDUE_B_NOBN	35 (81%)	2 (5%)	0 (0%)	4 (9%)
B_NOBN vs B1_NOBN vs B2_NOBN (section C.3)	FONDUE_B_NOBN	50 (100%)	21 (55%)	22 (44%)	27 (66%)
	FONDUE_B1_NOBN	0 (0%)	1 (3%)	3 (6%)	0 (0%)
	FONDUE_B2_NOBN	0 (0%)	16 (42%)	25 (50%)	14 (34%)
B_NOBN vs LT (section C.4)	FONDUE_B_NOBN	0 (0%)	2 (4%)	2 (4%)	1 (2%)
	FONDUE_LT	50 (100%)	48 (96%)	48 (96%)	49 (98%)
LT vs LT_×2 (section C.5)	FONDUE_LT	50 (100%)	24 (80%)	44 (100%)	21 (66%)
	FONDUE_LT_×2	0 (0%)	6 (20%)	0 (0%)	11 (34%)

2. Prototype networks: FONDUE_A vs FONDUE_B

Using paired t-tests, we compared the best versions of FONDUE_A (FONDUE_A_NOBN) and FONDUE_B (FONDUE_B_NOBN), correcting the results for multiple comparisons (see Table S.5)

As summarized in Table S.6 the best method differs based on the choice of metric. For LPIPS, FONDUE_B performed best as expected, as LPIPS was the loss function used for FONDUE_B. For the rest of the metrics, the clear winner was FONDUE_A, showing superiority over FONDUE_B in at least 90% of the cases. Despite FONDUE_A outperforming FONDUE_B in three out of the four metrics, as discussed in the introduction, LPIPS estimates of the similarity between images are more closely related to human perception. Additionally, one of the main objectives of the proposed method was to preserve the fine details in the image, and LPIPS has been shown to be a more sensitive metric for detecting image degradation in high-frequency regions. Therefore, based on the performance in terms of LPIPS, FONDUE_B was selected as the best-performing FONDUE version, and we will refer to FONDUE or FONDUE_B as the non-BN version of FONDUE_B if not indicated.

As can be seen in Figure S.1, FONDUE_B produces textures and high-frequency details that better resemble the ground truth image, followed by FONDUE_A. As a reference of performance of a non-DL method, we have included PRINLM, which produces blurry edges and cartoon-like textures. These features are better reflected by LPIPS, which indicates that the best method was indeed FONDUE_B. On the other hand, PSNR, MS-SSIM, and FLIP indicate that the best image amongst the three included in the figure is that of PRINLM, followed by FONDUE_A, and lastly FONDUE_B. According to SSIM, FONDUE_A was the best, followed by FONDUE_B and PRINLM. These observations led us to choose FONDUE_B as the best prototype network, despite having been trained on the same metric that the network was evaluated on and even though the rest of the metrics indicate FONDUE_A as the best performing network.

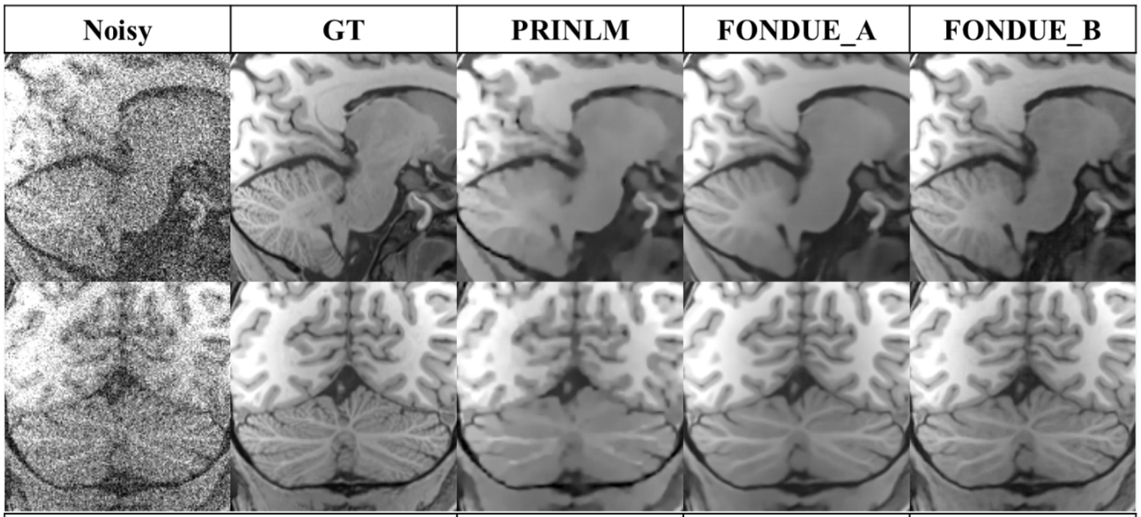

	PRINLM	FONDUE_A	FONDUE_B
**PSNR (dB) ↑**	**43.25**	38.77	38.38
**SSIM (relative) ↑**	0.989	**0.992**	0.990
**MS-SSIM (relative) ↑**	**0.996**	0.996	0.994
**FLIP (relative) ↓**	**0.016**	0.019	0.023
**LPIPS (relative) ↓**	0.009	0.008	**0.006**

Figure S.1: *Comparing performance metrics versus perceived quality. An image from the test set of the HCP cohort with 9% of stationary Rician noise added, denoised with PRINLM, FONDUE_A (non-BN), and FONDUE_B (non-BN) compared to their ground truth (GT) reference. The best result is in bold and the second-best result is underlined. According to PSNR, MS-SSIM, and FLIP, the best image was produced by PRINLM, and the worst one is FONDUE_B. However, the image with perceptually better quality in the cerebellar sulci and gyri was FONDUE_B. Furthermore, the overall texture can be perceived as more similar to the GT image, which relates more to the results reflected by LPIPS*

3. Two-stage networks:

Using paired t-tests followed by FDR correction, we assessed whether adding a second-stage network (FONDUE_B1 and FONDUE_B2) can improve the results of the best-performing stand-alone network (FONDUE_B without Batch Normalization, i.e., FONDUE_B_NOBN). We used the output of FONDUE_B as input to both networks, using only the non-batch-normalized versions of FONDUE_B1 and FONDUE_B2 based on the results of our previous experiments (Table S.5).

As summarized in Table S.5, there was no improvement in terms of LPIPS when using a second-stage network. However, in terms of PSNR, MS-SSIM, and SSIM there was an apparent performance boost in a subset of the cases, more specifically, in images subjected to significant levels of induced noise, specifically stationary Rician noise at concentrations of 7% and 9%, with some instances at 5% (as noted on Tables S.9 to S.14). These results suggest

that there is a smoothening of the images after using the second-stage networks since PSNR/SSIM/MS-SSIM favors smooth textures in images as opposed to LPIPS which captures the similarity in high-frequency image regions better. Therefore, this apparent gain in performance in these three metrics (without any improvement in LPIPS) is likely a product of blurring (see Figure S.5 - Figure S.10). Furthermore, FONDUE_B2 -which was trained on high levels of noise- showed superiority over FONDUE_B1, suggesting that the performance boost in these metrics was a result of blurring in cases where high noise levels are being added.

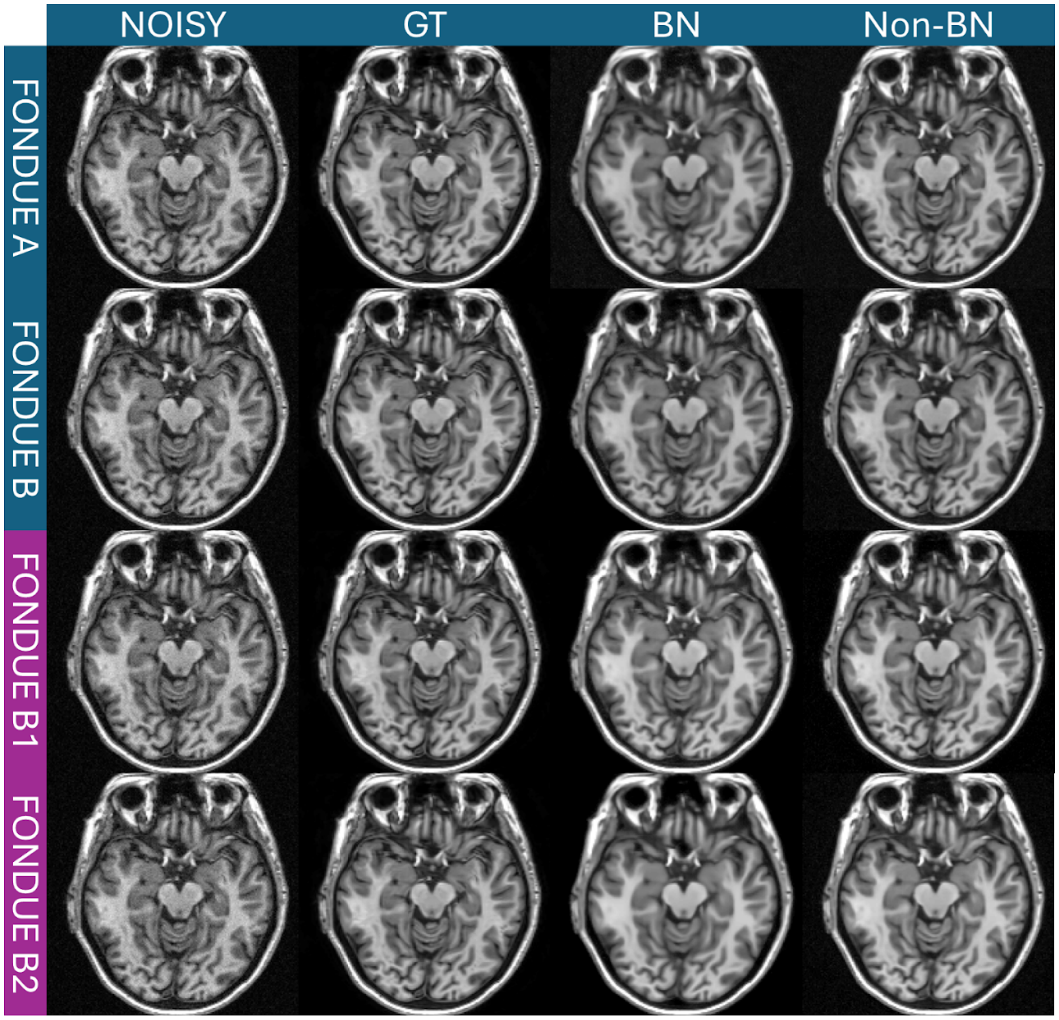

Figure S.2: *Effect of Batch Normalization on the appearance of the images on FONDUE_A, FONDUE_B, FONDUE_B1, and FONDUE_B2: axial slice on an ADNI subject from the test set. The non-Batch-Normalized (Non-BN column) has a sharper appearance than their Batch-Normalized (BN column) counterparts for both FONDUE_A and FONDUE_B, as well as for the second stage networks FONDUE_B1 and FONDUE_B2. The noisy images correspond to 1% added stationary Rician noise.*

4. FONDUE_LT vs FONDUE_B: the effect of training for longer.

Finally, to assess whether the performance of FONDUE_B could be further improved through additional training, FONDUE_B (initially trained for 10 epochs) was compared against FONDUE_LT, which was trained for 50 epochs while keeping all other training parameters consistent. Table S.5 compares the performances of the two networks, using paired t-tests.

For all metrics across all noise levels and datasets on the test set, there was a significant improvement going from FONDUE_B to FONDUE_LT. As such, FONDUE_LT was considered as the final optimal version of FONDUE and was included in the rest of the analyses (excluding

the second-stage methods). It is important to note that convergence was reached in epoch 47, and the weights used were those obtained at the end of that epoch.

5. Recursive use of FONDUE_LT

As discussed above, networks designed as second stages for FONDUE_B (e.g. FONDUE_B2) improved metrics such as PSNR, SSIM, and MS-SSIM. However, this improvement was likely due to over-smoothening of the images, especially in high-noise-level images. As such, any network may cause the same blurring effect when used as a second-stage network, even if not trained to receive the output of another FONDUE network as input. To test this, we recursively processed our test images using FONDUE_LT, i.e., we processed a noisy image twice with FONDUE_LT using the output of the first iteration as input to the second iteration. The results are shown below:

Similar to the results from the two-stage networks, no improvement was observed in LPIPS, with an apparent improvement in SSIM and MS-SSIM, even when the network was not trained for this specific scenario. These results suggest that two-stage network scenarios might inflate certain similarity metrics, while no gain in sharpness or deep-feature quality is achieved on the test-set images.

D. Results

1. Statistical test summary

After performing paired the t-tests described in section 2.7 we have summarized the frequency in which a given method was superior to the rest with statistical significance at $p<0.05$ (non-significant first places were not considered). Table S.6 summarizes this information, showing the frequency in which a given method was the best for a given metric with a p-value<0.05 (after adjusting for multiple comparisons). Based on these results, the best method was FONDUE_LT, followed by PRINLM and ODCT. Note that all methods were included in this comparison, but the table only includes methods that significantly outperformed other methods in at least one of the comparisons.

Table S.6: *Comparisons across methods: frequency of a method being significantly superior to the rest after correcting for FDR*

Method	Metric			
	LPIPS	MS-SSIM	PSNR	SSIM
AONLM	0 (0%)	0 (0%)	0 (0%)	0 (0%)
MRONLM	0 (0%)	0 (0%)	0 (0%)	0 (0%)
ODCT	7 (14%)	0 (0%)	0 (0%)	3 (6%)
ONLM	0 (0%)	0 (0%)	0 (0%)	1 (2%)
PRINLM	6 (12%)	8 (17%)	10 (20%)	7 (14%)
UNET_VINN	0 (0%)	0 (0%)	0 (0%)	0 (0%)
MCDnCNN	0 (0%)	0 (0%)	0 (0%)	0 (0%)
FONDUE_A	0 (0%)	5 (11%)	3 (6%)	12 (24%)
FONDUE_B	0 (0%)	0 (0%)	0 (0%)	0 (0%)
FONDUE_LT	37 (74%)	35 (76%)	37 (74%)	39 (54%)

2. Visual assessments

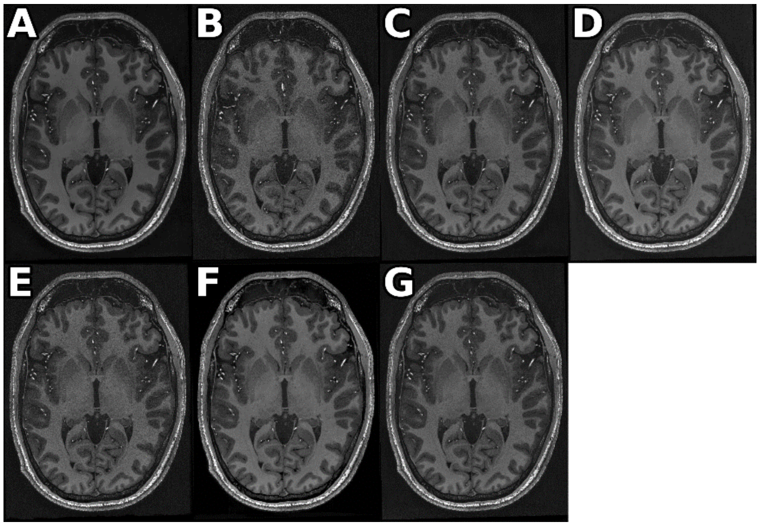

Figure S.3: *Example of a blind visual assessment grid. This image corresponds to the UH_250 dataset for the stand-alone methods. Each letter was assigned randomly to each of the sub-images and the raters blindly classified the 1st, 2nd, and 3rd best-looking image in terms of presence of noise and overall image quality.*

Table S.7: *Visual assessment protocol results: Stand-alone networks. "Best" indicates that the given Rater assessed a given image denoised with the indicated method as the best for a given resolution. "2nd or 3rd best" indicates that a given image denoised with the indicated method was either the 2nd or the 3rd best. "Inter-rater agreement" indicates that both raters agreed in the assessment for a given method.*

Resolution	Method	Best – Rater 1	Best – Rater 2	2nd or 3rd best – Rater 1	2nd or 3rd best – Rater 2	Best - Inter-rater agreement	2nd or 3rd best – Inter-rater agreement
0.25mm³	FONDUE_A_BN	00.0% (0)	00.0% (0)	66.7% (6)	100.0% (9)	00.0% (0)	66.7% (6)
	FONDUE_A_NOBN	00.0% (0)	00.0% (0)	11.1% (1)	00.0% (0)	00.0% (0)	00.0% (0)
	FONDUE_B_BN	00.0% (0)	00.0% (0)	44.4% (4)	00.0% (0)	00.0% (0)	00.0% (0)
	FONDUE_B_NOBN	100.0% (9)	88.9% (8)	00.0% (0)	11.1% (1)	88.9% (8)	00.0% (0)
	FONDUE_LT	00.0% (0)	00.0% (0)	00.0% (0)	00.0% (0)	00.0% (0)	00.0% (0)
	UNET_VINN	00.0% (0)	11.1% (1)	77.8% (7)	88.9% (8)	00.0% (0)	66.7% (6)
	MCDnCNN	00.0% (0)	00.0% (0)	00.0% (0)	00.0% (0)	00.0% (0)	00.0% (0)
0.5mm³	FONDUE_A_BN	22.2% (2)	55.6% (5)	22.2% (2)	44.4% (4)	11.1% (1)	11.1% (1)
	FONDUE_A_NOBN	00.0% (0)	00.0% (0)	11.1% (1)	11.1% (1)	00.0% (0)	11.1% (1)
	FONDUE_B_BN	11.1% (1)	00.0% (0)	44.4% (4)	55.6% (5)	00.0% (0)	33.3% (3)
	FONDUE_B_NOBN	44.4% (4)	33.3% (3)	44.4% (4)	66.7% (6)	00.0% (0)	11.1% (1)
	FONDUE_LT	00.0% (0)	00.0% (0)	11.1% (1)	00.0% (0)	00.0% (0)	00.0% (0)
	UNET_VINN	00.0% (0)	00.0% (0)	44.4% (4)	22.2% (2)	00.0% (0)	11.1% (1)
	MCDnCNN	22.2% (2)	11.1% (1)	22.2% (2)	00.0% (0)	00.0% (0)	00.0% (0)
1.0mm³	FONDUE_A_BN	11.1% (1)	11.1% (1)	11.1% (1)	22.2% (2)	00.0% (0)	00.0% (0)
	FONDUE_A_NOBN	11.1% (1)	11.1% (1)	22.2% (2)	55.6% (5)	00.0% (0)	11.1% (1)
	FONDUE_B_BN	11.1% (1)	00.0% (0)	11.1% (1)	44.4% (4)	00.0% (0)	00.0% (0)
	FONDUE_B_NOBN	22.2% (2)	22.2% (2)	33.3% (3)	22.2% (2)	00.0% (0)	00.0% (0)
	FONDUE_LT	33.3% (3)	22.2% (2)	11.1% (1)	22.2% (2)	00.0% (0)	00.0% (0)
	UNET_VINN	11.1% (1)	11.1% (1)	66.7% (6)	22.2% (2)	00.0% (0)	22.2% (2)
	MCDnCNN	00.0% (0)	22.2% (2)	44.4% (4)	11.1% (1)	00.0% (0)	11.1% (1)

Table S.8: *Visual assessment protocol results: Two-stage networks. "Best" indicates that the given Rater assessed a given image denoised with the indicated method as the best for a given resolution. "2nd or 3rd best" indicates that a given image denoised with the indicated method was either the 2nd or the 3rd best. "Inter-rater agreement" indicates that both raters agreed in the assessment for a given method.*

Resolution	Method	Best – Rater 1	Best – Rater 2	2nd or 3rd best – Rater 1	2nd or 3rd best – Rater 2	Best - Inter-rater agreement	2nd or 3rd best – Inter-rater agreement
0.25mm³	FONDUE_B1_BN	00.0% (0)	00.0% (0)	11.1% (1)	00.0% (0)	00.0% (0)	00.0% (0)
	FONDUE_B1_NOBN	11.1% (1)	11.1% (1)	88.9% (8)	77.8% (7)	00.0% (0)	66.7% (6)
	FONDUE_B2_BN	00.0% (0)	00.0% (0)	88.9% (8)	100.0% (9)	00.0% (0)	88.9% (8)
	FONDUE_B2_NOBN	88.9% (8)	88.9% (8)	11.1% (1)	11.1% (1)	77.8% (7)	00.0% (0)
	FONDUE_LT_X2	00.0% (0)	00.0% (0)	00.0% (0)	11.1% (1)	00.0% (0)	00.0% (0)
0.5mm³	FONDUE_B1_BN	22.2% (2)	33.3% (3)	22.2% (2)	33.3% (3)	22.2% (2)	11.1% (1)
	FONDUE_B1_NOBN	11.1% (1)	00.0% (0)	66.7% (6)	66.7% (6)	00.0% (0)	44.4% (4)
	FONDUE_B2_BN	11.1% (1)	22.2% (2)	77.8% (7)	55.6% (5)	11.1% (1)	55.6% (5)
	FONDUE_B2_NOBN	22.2% (2)	22.2% (2)	33.3% (3)	44.4% (4)	00.0% (0)	22.2% (2)
	FONDUE_LT_X2	33.3% (3)	22.2% (2)	00.0% (0)	00.0% (0)	00.0% (0)	00.0% (0)
1.0mm³	FONDUE_B1_BN	00.0% (0)	44.4% (4)	77.8% (7)	55.6% (5)	00.0% (0)	55.6% (5)
	FONDUE_B1_NOBN	66.7% (6)	33.3% (3)	33.3% (3)	55.6% (5)	11.1% (1)	11.1% (1)
	FONDUE_B2_BN	00.0% (0)	00.0% (0)	33.3% (3)	33.3% (3)	00.0% (0)	22.2% (2)
	FONDUE_B2_NOBN	33.3% (3)	22.2% (2)	55.6% (5)	55.6% (5)	11.1% (1)	33.3% (3)
	FONDUE_LT_X2	00.0% (0)	00.0% (0)	00.0% (0)	00.0% (0)	00.0% (0)	00.0% (0)

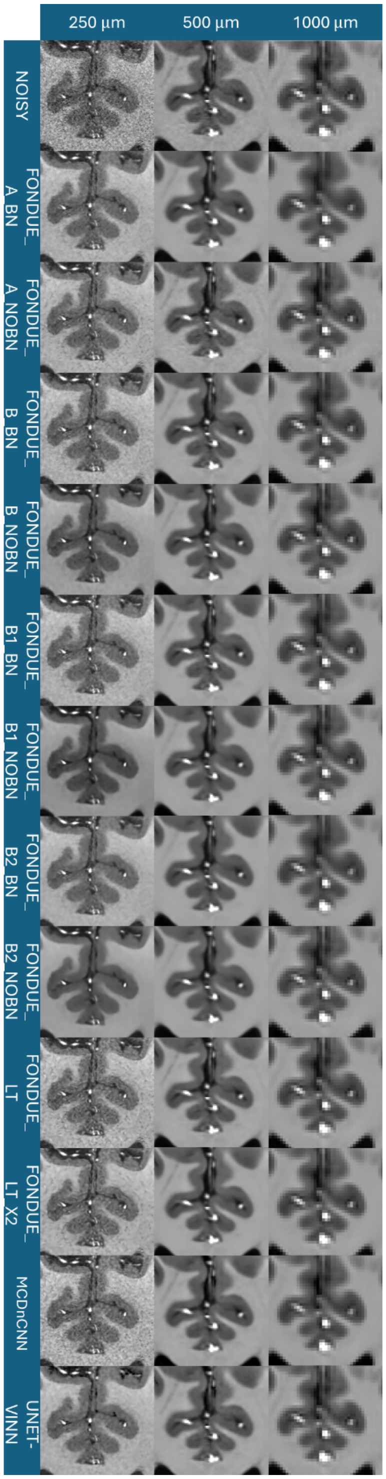

Figure S.4: *UH_NNN dataset denoised at three different resolutions by the different DL versions at 0.25mm isotropic voxel size. First column shows UH_250, middle column shows UH_500, and right column shows UH_1000.*

3. Segmentation after denoising – FastSurfer regions merged

To summarize the results, we merged the segmentation masks into 5 main regions ventricles (regions 4, 5, 43, 44, 14, and 15), cerebellar cortex (regions 8 and 47), cerebellar white matter (regions 46 and 7), white matter (regions 2, 10, 11, 12, 13, 16, 17, 18, 26, 28, 31, 41, 49, 50, 51, 52, 53, 54, 58, 60, 63, and 77), and grey matter (regions 1002, 1003, 2003, 1005, 1006, 2006, 1007, 2007, 1008, 2008, 1009, 2009, 1010, 1011, 2011, 1012, 1013, 1014, 1015, 2015, 1016, 1017, 1018, 2018, 1019, 2019, 1020, 2020, 1021, 1022, 1023, 1024, 1025, 1026, 2026, 1027, 2027, 1028, 1029, 2029, 1030, 2030, 1031, 2031, 1034, 2034, 1035, 2035, 2002, 2005, 2010, 2012, 2013, 2014, 2016, 2017, 2021, 2022, 2023, 2024, 2025, and 2028)

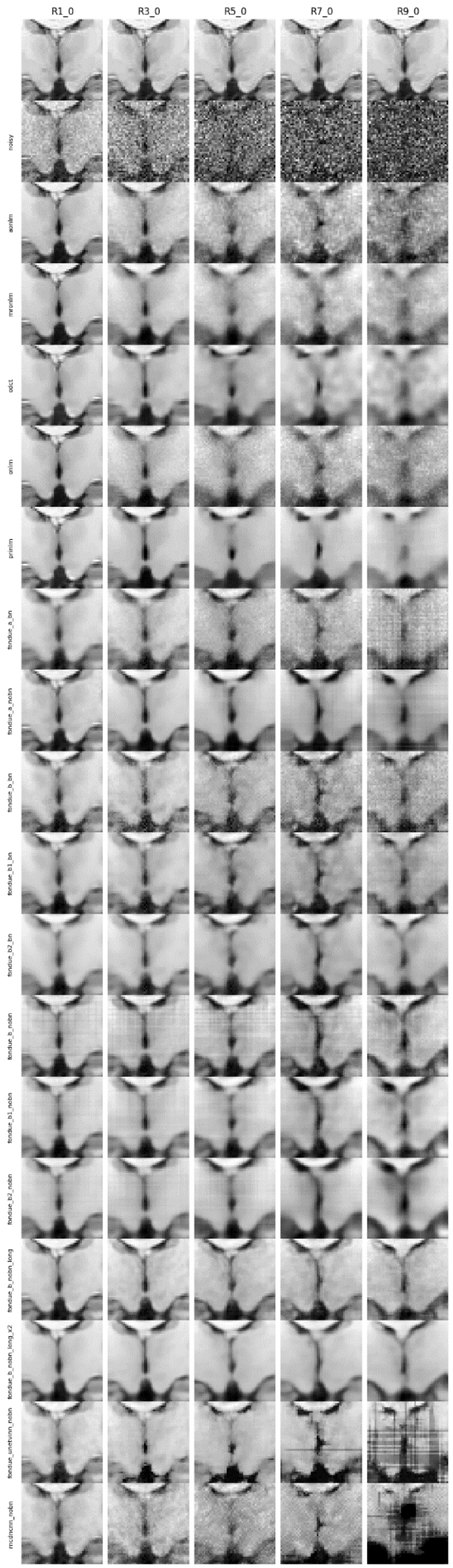

Figure S.5*: Results of all the compared denoising methods for different levels of stationary Rician noise on the ABIDE-II test set. The header column R means stationary Rician noise, and "N_0" represents N% of added noise.*

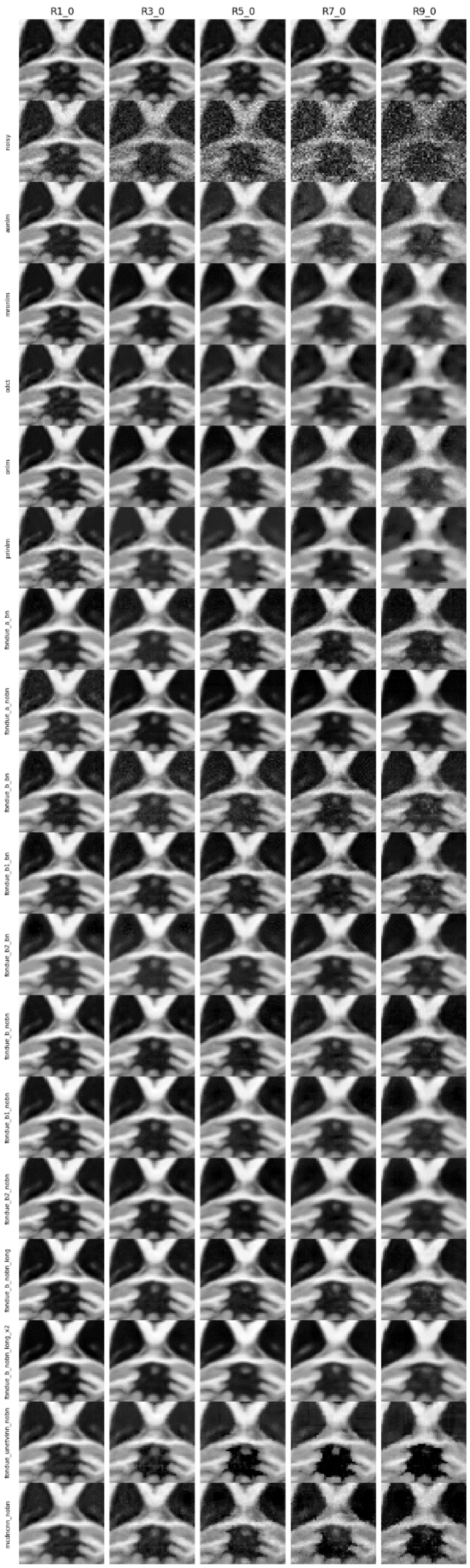

Figure S.6: *Results of all the compared denoising methods for different levels of stationary Rician noise on the ADNI1 test set. The header column R means stationary Rician noise, and "N_0" represents N% of added noise.*

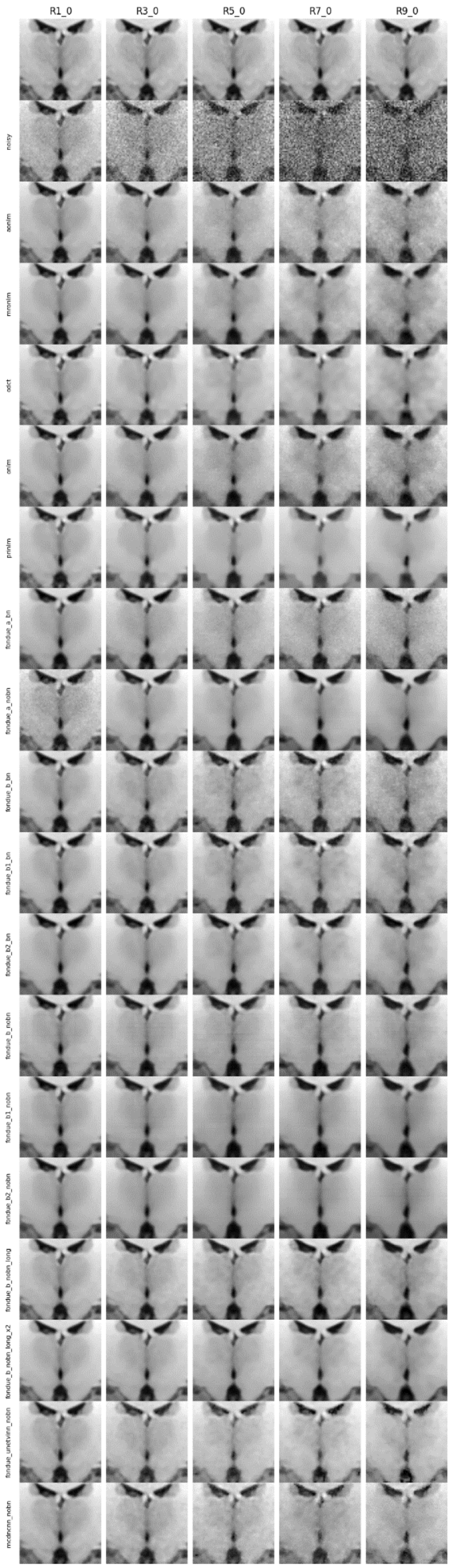

Figure S.7: *Results of all the compared denoising methods for different levels of stationary Rician noise on the HCP test set. The header column R means stationary Rician noise, and "N_0" represents N% of added noise.*

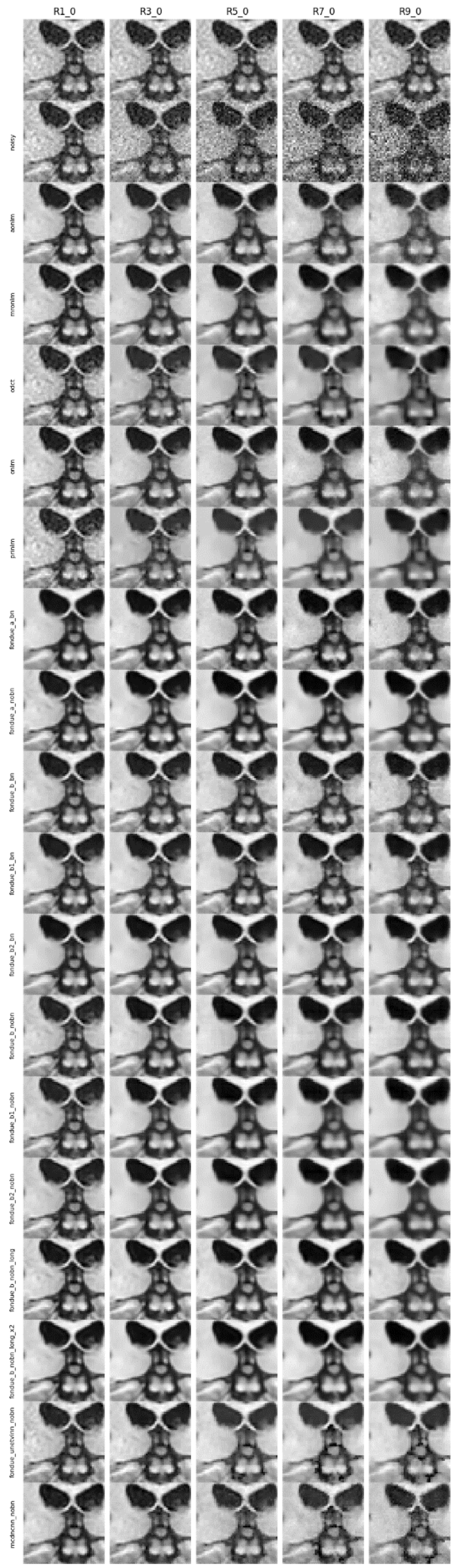

Figure S.8: *Results of all the compared denoising methods for different levels of stationary Rician noise on the IXI test set. The header column R means stationary Rician noise, and "N_0" represents N% of added noise.*

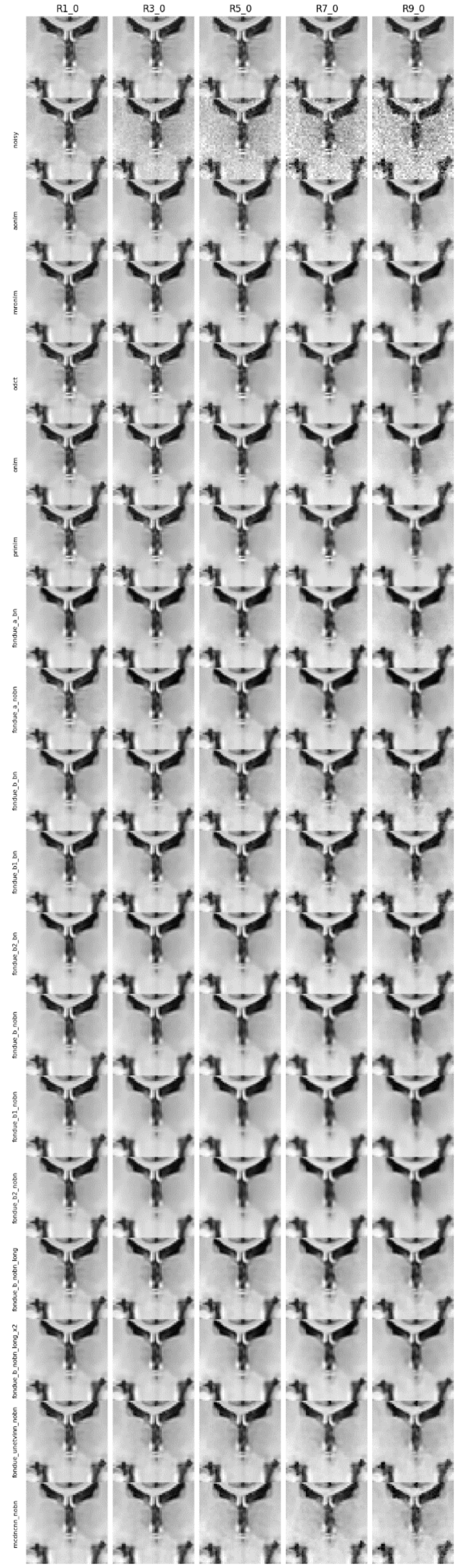

Figure S.9: *Results of all the compared denoising methods for different levels of stationary Rician noise on the LA5c test set. The header column R means stationary Rician noise, and "N_0" represents N% of added noise.*

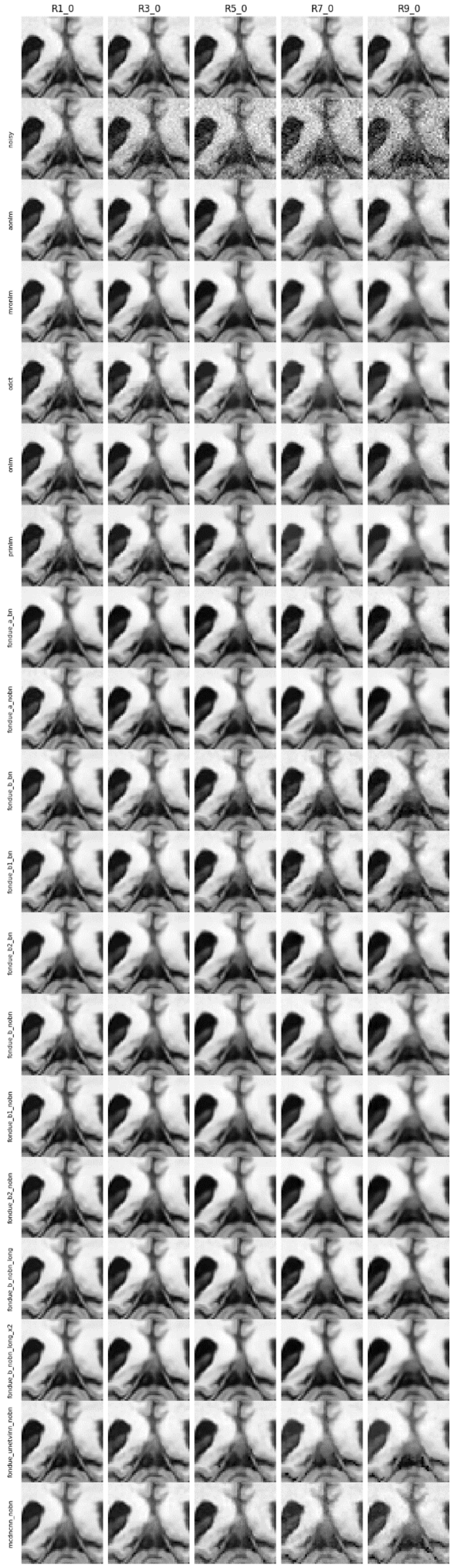

Figure S.10: *Results of all the compared denoising methods for different levels of stationary Rician noise on the MIRIAD test set. The header column R means stationary Rician noise, and "N_0" represents N% of added noise*

Table S.9: *Denoising performance comparison across noise levels, metrics, and methods for added stationary Rician noise on ABIDE-II test set.*

	Method	1%	3%	5%	7%	9%
**LPIPS (lower is better): mean ± standard deviation**	**MCDnCNN**	0.001 ± 4e-04	0.0026 ± 0.0041	0.0058 ± 0.0103	0.0098 ± 0.0175	0.0164 ± 0.0272
	**UNET_VINN**	8e-04 ± 4e-04	0.0019 ± 0.002	0.0041 ± 0.0059	0.0064 ± 0.0086	0.0089 ± 0.011
	**FONDUE_A**	7e-04 ± 3e-04	0.0014 ± 4e-04	0.0021 ± 5e-04	0.0028 ± 6e-04	0.0037 ± 7e-04
	**FONDUE_B**	7e-04 ± 2e-04	0.0014 ± 3e-04	0.002 ± 4e-04	0.0026 ± 6e-04	0.0034 ± 0.001
	**FONDUE_LT**	5e-04 ± 2e-04	0.001 ± 2e-04	0.0016 ± 3e-04	0.0021 ± 4e-04	0.0028 ± 7e-04
	**AONLM**	5e-04 ± 2e-04	0.0016 ± 3e-04	0.0025 ± 4e-04	0.0035 ± 8e-04	0.0051 ± 0.0016
	**MRONLM**	7e-04 ± 3e-04	0.0025 ± 5e-04	0.0034 ± 8e-04	0.0038 ± 0.001	0.0041 ± 0.0011
	**ONLM**	7e-04 ± 3e-04	0.0022 ± 5e-04	0.0031 ± 6e-04	0.0037 ± 8e-04	0.0043 ± 0.0011
	**ODCT**	3e-04 ± 1e-04	0.0013 ± 5e-04	0.0023 ± 7e-04	0.0031 ± 8e-04	0.0038 ± 0.001
	**PRINLM**	0.0271 ± 0.0423	0.0315 ± 0.0422	0.0286 ± 0.041	0.0327 ± 0.0413	0.0441 ± 0.0455
	**FONDUE_A_BN**	0.0036 ± 0.0017	0.004 ± 0.002	0.0061 ± 0.0034	0.0061 ± 0.0025	0.0115 ± 0.0057
	**FONDUE_B_BN**	0.0011 ± 4e-04	0.0013 ± 5e-04	0.0018 ± 4e-04	0.0028 ± 9e-04	0.0037 ± 0.0015
	**FONDUE_B1_BN**	0.0012 ± 4e-04	0.0015 ± 4e-04	0.002 ± 4e-04	0.0027 ± 5e-04	0.0033 ± 8e-04
	**FONDUE_B2_BN**	0.002 ± 5e-04	0.0021 ± 6e-04	0.0025 ± 5e-04	0.0032 ± 6e-04	0.0038 ± 9e-04
	**FONDUE_B1_NOBN**	0.001 ± 3e-04	0.0018 ± 4e-04	0.0025 ± 6e-04	0.0032 ± 7e-04	0.0042 ± 0.0011
	**FONDUE_B2_NOBN**	0.0015 ± 0.0012	0.0024 ± 0.0017	0.0034 ± 0.0025	0.0043 ± 0.0029	0.0054 ± 0.0033
	**FONDUE_LT_X2**	0.0011 ± 3e-04	0.0015 ± 3e-04	0.0021 ± 5e-04	0.0027 ± 7e-04	0.0034 ± 9e-04
**PSNR in dB (higher is better): mean ± standard deviation**	**MCDnCNN**	50.7739 ± 2.2803	47.4151 ± 1.9438	44.5065 ± 2.584	42.5278 ± 3.0258	40.24 ± 3.46
	**UNET_VINN**	49.0948 ± 2.9553	45.4932 ± 2.1658	43.1569 ± 2.0697	41.7122 ± 1.8687	40.3867 ± 1.9372
	**FONDUE_A**	50.8338 ± 2.7677	47.5066 ± 2.4961	46.7028 ± 2.5602	46.1007 ± 2.1898	44.9825 ± 1.545
	**FONDUE_B**	51.5855 ± 3.6407	46.4535 ± 2.7384	45.2155 ± 2.2926	44.9106 ± 1.9618	44.0192 ± 1.5042
	**FONDUE_LT**	51.9746 ± 3.5244	48.7042 ± 2.8113	47.039 ± 2.4394	45.9151 ± 2.0572	44.7587 ± 1.6203
	**AONLM**	48.1715 ± 1.9857	42.27 ± 2.1849	40.6248 ± 1.6657	39.14 ± 1.8242	37.6792 ± 1.6993
	**MRONLM**	46.7732 ± 2.1546	40.5465 ± 1.9432	39.324 ± 1.8076	38.7338 ± 2.1478	37.6 ± 1.5698
	**ONLM**	46.7218 ± 2.195	40.7262 ± 1.7595	39.5177 ± 1.5466	39.1046 ± 1.7499	38.0589 ± 1.2097
	**ODCT**	49.6579 ± 2.1513	43.6065 ± 1.9377	41.7572 ± 1.7726	40.3536 ± 1.5913	38.7093 ± 1.3633
	**PRINLM**	38.9493 ± 15.0358	33.4574 ± 12.7823	34.0256 ± 11.7282	31.1253 ± 11.0842	28.8177 ± 11.3679
	**FONDUE_A_BN**	41.2019 ± 4.7282	40.1438 ± 4.5798	37.581 ± 4.475	37.3607 ± 3.3221	33.9682 ± 4.3719
	**FONDUE_B_BN**	48.6145 ± 2.056	46.5803 ± 1.6869	44.4201 ± 1.7103	42.8433 ± 1.2858	42.2851 ± 1.6794
	**FONDUE_B1_BN**	48.038 ± 2.1549	46.42 ± 2.0306	44.4504 ± 1.9542	43.002 ± 1.4637	42.6161 ± 1.7562
	**FONDUE_B2_BN**	45.7244 ± 2.1827	44.6811 ± 1.9402	43.2959 ± 1.8297	42.0463 ± 1.4487	41.9091 ± 1.699
	**FONDUE_B1_NOBN**	50.7781 ± 3.3487	46.0139 ± 2.5519	44.9827 ± 2.2336	44.7211 ± 1.9038	43.6385 ± 1.5102
	**FONDUE_B2_NOBN**	48.6145 ± 2.8479	44.4318 ± 1.9481	43.4557 ± 1.8626	43.2774 ± 1.7645	42.7088 ± 1.5527
	**FONDUE_LT_X2**	49.3483 ± 3.7654	47.1818 ± 3.2233	45.8252 ± 2.7629	44.81 ± 2.3542	43.8045 ± 1.9294
**SSIM (higher is better): mean ± standard deviation**	**MCDnCNN**	0.9987 ± 4e-04	0.9958 ± 0.0038	0.9915 ± 0.0095	0.9876 ± 0.0142	0.9819 ± 0.0188
	**UNET_VINN**	0.9987 ± 5e-04	0.9968 ± 0.0018	0.9939 ± 0.0053	0.9913 ± 0.0074	0.9883 ± 0.0088
	**FONDUE_A**	0.9986 ± 6e-04	0.998 ± 3e-04	0.9972 ± 4e-04	0.9963 ± 4e-04	0.9953 ± 5e-04
	**FONDUE_B**	0.9989 ± 3e-04	0.9977 ± 3e-04	0.9966 ± 4e-04	0.9955 ± 5e-04	0.9942 ± 9e-04
	**FONDUE_LT**	0.9991 ± 3e-04	0.998 ± 3e-04	0.9969 ± 3e-04	0.9959 ± 4e-04	0.9948 ± 6e-04
	**AONLM**	0.9983 ± 6e-04	0.9952 ± 0.0013	0.9924 ± 0.0019	0.9895 ± 0.0024	0.9866 ± 0.0031
	**MRONLM**	0.9982 ± 6e-04	0.9945 ± 0.0014	0.9927 ± 0.0016	0.9911 ± 0.0017	0.9893 ± 0.0018
	**ONLM**	0.9983 ± 7e-04	0.9947 ± 0.0014	0.9922 ± 0.0017	0.9901 ± 0.0019	0.9879 ± 0.0021
	**ODCT**	0.9986 ± 6e-04	0.9957 ± 0.0011	0.9936 ± 0.0015	0.9916 ± 0.0016	0.9899 ± 0.0017
	**PRINLM**	0.979 ± 0.0308	0.9741 ± 0.03	0.9754 ± 0.0281	0.9712 ± 0.0278	0.965 ± 0.0282
	**FONDUE_A_BN**	0.9954 ± 0.0025	0.9943 ± 0.0026	0.9909 ± 0.0044	0.9902 ± 0.0035	0.9832 ± 0.0078
	**FONDUE_B_BN**	0.9985 ± 4e-04	0.9972 ± 8e-04	0.9957 ± 7e-04	0.9941 ± 0.0011	0.9926 ± 0.0013
	**FONDUE_B1_BN**	0.9985 ± 4e-04	0.9976 ± 4e-04	0.9965 ± 4e-04	0.9952 ± 6e-04	0.9942 ± 8e-04
	**FONDUE_B2_BN**	0.9977 ± 5e-04	0.9972 ± 5e-04	0.9964 ± 4e-04	0.9953 ± 6e-04	0.9945 ± 7e-04
	**FONDUE_B1_NOBN**	0.9988 ± 3e-04	0.9976 ± 3e-04	0.9966 ± 5e-04	0.9956 ± 6e-04	0.9943 ± 0.0013
	**FONDUE_B2_NOBN**	0.9984 ± 9e-04	0.997 ± 0.0013	0.9958 ± 0.0021	0.9947 ± 0.0028	0.9933 ± 0.0037
	**FONDUE_LT_X2**	0.9986 ± 4e-04	0.9978 ± 4e-04	0.9969 ± 4e-04	0.9959 ± 5e-04	0.9948 ± 8e-04
**MSSSIM (higher is better): mean ± standard deviation**	**MCDnCNN**	0.9994 ± 2e-04	0.9977 ± 0.0041	0.9936 ± 0.0138	0.9885 ± 0.0231	0.9786 ± 0.0353
	**UNET_VINN**	0.9991 ± 6e-04	0.9978 ± 0.0024	0.9955 ± 0.0072	0.9934 ± 0.0104	0.9907 ± 0.013
	**FONDUE_A**	0.9995 ± 2e-04	0.999 ± 3e-04	0.9987 ± 3e-04	0.9983 ± 3e-04	0.9976 ± 8e-04
	**FONDUE_B**	0.9995 ± 2e-04	0.9988 ± 3e-04	0.9982 ± 5e-04	0.9978 ± 7e-04	0.9969 ± 0.0013
	**FONDUE_LT**	0.9995 ± 3e-04	0.9991 ± 3e-04	0.9987 ± 3e-04	0.9982 ± 5e-04	0.9976 ± 8e-04
	**AONLM**	0.9996 ± 1e-04	0.9986 ± 7e-04	0.9978 ± 9e-04	0.9968 ± 0.0014	0.9955 ± 0.0025
	**MRONLM**	0.9994 ± 2e-04	0.9982 ± 9e-04	0.9975 ± 9e-04	0.997 ± 9e-04	0.9963 ± 9e-04
	**ONLM**	0.9995 ± 2e-04	0.9983 ± 7e-04	0.9976 ± 8e-04	0.9971 ± 9e-04	0.9964 ± 8e-04
	**ODCT**	0.9996 ± 1e-04	0.9989 ± 3e-04	0.9982 ± 6e-04	0.9974 ± 8e-04	0.9966 ± 9e-04
	**PRINLM**	0.9703 ± 0.0462	0.9636 ± 0.0496	0.9693 ± 0.0455	0.9625 ± 0.0487	0.9517 ± 0.0513
	**FONDUE_A_BN**	0.9947 ± 0.0039	0.9937 ± 0.0041	0.9893 ± 0.0076	0.9903 ± 0.0063	0.9794 ± 0.0148
	**FONDUE_B_BN**	0.9992 ± 3e-04	0.9988 ± 5e-04	0.9981 ± 6e-04	0.9972 ± 0.0012	0.9967 ± 0.0013
	**FONDUE_B1_BN**	0.9991 ± 3e-04	0.9988 ± 4e-04	0.9981 ± 6e-04	0.9974 ± 0.001	0.997 ± 0.0012
	**FONDUE_B2_BN**	0.9986 ± 5e-04	0.9983 ± 7e-04	0.9978 ± 7e-04	0.997 ± 0.0011	0.9967 ± 0.0012
	**FONDUE_B1_NOBN**	0.9994 ± 2e-04	0.9987 ± 4e-04	0.9981 ± 7e-04	0.9976 ± 0.001	0.9964 ± 0.002
	**FONDUE_B2_NOBN**	0.9988 ± 0.0012	0.9978 ± 0.0019	0.997 ± 0.0031	0.9962 ± 0.0042	0.9949 ± 0.0055
	**FONDUE_LT_X2**	0.9992 ± 4e-04	0.9988 ± 4e-04	0.9984 ± 5e-04	0.9979 ± 7e-04	0.9971 ± 0.0012

Table S.10: *Denoising performance comparison across noise levels, metrics, and methods for added stationary Rician noise on ADNI1 test set.*

	Method	1%	3%	5%	7%	9%
**LPIPS (lower is better): mean ± standard deviation**	**MCDnCNN**	0.0019 ± 0.0012	0.007 ± 0.0112	0.0153 ± 0.0251	0.0205 ± 0.0292	0.0268 ± 0.0346
	**UNET_VINN**	0.001 ± 4e-04	0.0032 ± 0.0027	0.0092 ± 0.0126	0.016 ± 0.022	0.0215 ± 0.0282
	**FONDUE_A**	6e-04 ± 2e-04	0.0016 ± 5e-04	0.003 ± 9e-04	0.0046 ± 0.0013	0.0064 ± 0.0023
	**FONDUE_B**	9e-04 ± 4e-04	0.0017 ± 5e-04	0.0027 ± 0.001	0.0039 ± 0.0016	0.0055 ± 0.0024
	**FONDUE_LT**	6e-04 ± 2e-04	0.0012 ± 4e-04	0.002 ± 6e-04	0.0031 ± 0.001	0.0043 ± 0.0015
	**AONLM**	5e-04 ± 2e-04	0.0021 ± 8e-04	0.0048 ± 0.0019	0.0084 ± 0.0036	0.0132 ± 0.0059
	**MRONLM**	8e-04 ± 3e-04	0.0037 ± 0.0014	0.0062 ± 0.0024	0.0078 ± 0.0029	0.009 ± 0.0033
	**ONLM**	8e-04 ± 3e-04	0.0035 ± 0.0014	0.006 ± 0.0024	0.0078 ± 0.0031	0.0096 ± 0.0037
	**ODCT**	4e-04 ± 2e-04	0.0018 ± 7e-04	0.0037 ± 0.0014	0.0057 ± 0.0021	0.0077 ± 0.0028
	**PRINLM**	5e-04 ± 2e-04	0.002 ± 7e-04	0.0038 ± 0.0013	0.0057 ± 0.002	0.0079 ± 0.0029
	**FONDUE_A_BN**	0.0023 ± 6e-04	0.0031 ± 0.0012	0.0045 ± 0.002	0.0077 ± 0.0046	0.0096 ± 0.005
	**FONDUE_B_BN**	0.0013 ± 4e-04	0.0027 ± 0.0017	0.0041 ± 0.002	0.0056 ± 0.0024	0.0076 ± 0.0033
	**FONDUE_B1_BN**	0.0015 ± 5e-04	0.0021 ± 8e-04	0.0034 ± 0.0013	0.0052 ± 0.0019	0.0067 ± 0.0025
	**FONDUE_B2_BN**	0.0026 ± 0.0012	0.0028 ± 0.0012	0.0035 ± 0.001	0.0049 ± 0.0015	0.0063 ± 0.0021
	**FONDUE_B1_NOBN**	0.0012 ± 6e-04	0.0022 ± 8e-04	0.0034 ± 0.0014	0.0047 ± 0.0019	0.0064 ± 0.0028
	**FONDUE_B2_NOBN**	0.002 ± 0.0016	0.0031 ± 0.0021	0.0049 ± 0.0039	0.007 ± 0.0049	0.0092 ± 0.0061
	**FONDUE_LT_X2**	0.0012 ± 4e-04	0.0018 ± 7e-04	0.0027 ± 0.001	0.0038 ± 0.0016	0.0051 ± 0.0023
**PSNR in dB (higher is better): mean ± standard deviation**	**MCDnCNN**	51.0581 ± 1.6358	46.4635 ± 2.5739	43.3681 ± 2.9597	41.1301 ± 2.7424	39.4089 ± 2.8588
	**UNET_VINN**	51.5466 ± 1.8953	46.8249 ± 1.3517	43.6238 ± 2.0353	41.5489 ± 2.3393	40.3689 ± 2.5697
	**FONDUE_A**	54.0321 ± 2.8024	51.0622 ± 2.7848	48.2001 ± 2.4089	46.0651 ± 1.8467	44.5831 ± 1.6728
	**FONDUE_B**	53.4032 ± 3.1824	49.2757 ± 2.7465	46.6746 ± 2.5575	44.8974 ± 2.3864	43.2542 ± 2.2083
	**FONDUE_LT**	55.0641 ± 3.1228	51.4152 ± 2.0992	48.8725 ± 2.1532	46.8746 ± 1.8252	45.4545 ± 1.509
	**AONLM**	55.2957 ± 3.0201	49.9756 ± 1.8311	46.2175 ± 1.3178	43.3823 ± 0.8881	40.703 ± 1.1415
	**MRONLM**	53.4695 ± 3.5909	48.4904 ± 2.6806	46.0016 ± 2.7709	44.5701 ± 2.9682	43.5421 ± 2.699
	**ONLM**	53.4001 ± 3.5368	48.4454 ± 2.6696	45.8675 ± 2.7051	44.4587 ± 2.7811	43.2382 ± 2.5787
	**ODCT**	56.7291 ± 2.0493	51.2214 ± 2.2966	48.4414 ± 2.1816	46.4867 ± 2.1709	45.1533 ± 2.1592
	**PRINLM**	56.8526 ± 2.3208	51.2483 ± 2.1696	48.3031 ± 1.9637	46.3829 ± 2.213	44.7738 ± 1.9889
	**FONDUE_A_BN**	50.3467 ± 1.9449	48.4663 ± 1.522	45.7681 ± 1.4875	43.2706 ± 1.1672	42.87 ± 1.3371
	**FONDUE_B_BN**	51.1723 ± 1.8776	48.3204 ± 1.0978	45.5479 ± 1.0923	43.6729 ± 1.3867	42.8937 ± 1.4858
	**FONDUE_B1_BN**	50.8475 ± 2.2322	48.9475 ± 1.9877	46.1422 ± 1.8368	43.946 ± 1.7364	43.2168 ± 1.8489
	**FONDUE_B2_BN**	48.3894 ± 2.1211	47.3452 ± 1.6967	45.7775 ± 2.1206	43.9999 ± 2.1199	43.2758 ± 1.8737
	**FONDUE_B1_NOBN**	52.1699 ± 2.6301	48.5904 ± 2.5299	46.2073 ± 2.4228	44.6656 ± 2.2444	43.0406 ± 2.3981
	**FONDUE_B2_NOBN**	49.831 ± 1.937	46.9589 ± 2.0025	45.0587 ± 2.2934	43.54 ± 1.9417	42.1526 ± 1.7665
	**FONDUE_LT_X2**	53.2111 ± 3.335	50.7769 ± 2.4985	48.4938 ± 2.3257	46.6471 ± 1.8594	45.1632 ± 1.4364
**SSIM (higher is better): mean ± standard deviation**	**MCDnCNN**	0.9971 ± 0.0015	0.9911 ± 0.0087	0.9848 ± 0.0151	0.9796 ± 0.0179	0.9732 ± 0.0217
	**UNET_VINN**	0.9977 ± 0.0012	0.9939 ± 0.0036	0.988 ± 0.0094	0.9821 ± 0.0145	0.9772 ± 0.0184
	**FONDUE_A**	0.9988 ± 3e-04	0.9977 ± 6e-04	0.9961 ± 8e-04	0.9944 ± 0.0011	0.9926 ± 0.0017
	**FONDUE_B**	0.9987 ± 4e-04	0.9972 ± 6e-04	0.9954 ± 0.0011	0.9934 ± 0.0017	0.9909 ± 0.0028
	**FONDUE_LT**	0.999 ± 3e-04	0.9975 ± 6e-04	0.9962 ± 8e-04	0.9946 ± 0.001	0.9929 ± 0.0015
	**AONLM**	0.999 ± 3e-04	0.9968 ± 7e-04	0.9938 ± 0.0014	0.9902 ± 0.0023	0.9857 ± 0.0038
	**MRONLM**	0.9989 ± 3e-04	0.9964 ± 8e-04	0.994 ± 0.0014	0.9922 ± 0.0018	0.9904 ± 0.0022
	**ONLM**	0.9989 ± 3e-04	0.9964 ± 8e-04	0.9938 ± 0.0014	0.9917 ± 0.002	0.9894 ± 0.0024
	**ODCT**	0.9991 ± 2e-04	0.9973 ± 7e-04	0.9953 ± 0.0012	0.9932 ± 0.0017	0.9912 ± 0.0021
	**PRINLM**	0.9991 ± 2e-04	0.9973 ± 7e-04	0.9953 ± 0.0011	0.9931 ± 0.0017	0.9909 ± 0.0022
	**FONDUE_A_BN**	0.9975 ± 5e-04	0.9961 ± 9e-04	0.9942 ± 0.0013	0.9911 ± 0.0025	0.9895 ± 0.0026
	**FONDUE_B_BN**	0.9979 ± 5e-04	0.9958 ± 0.0014	0.9935 ± 0.0017	0.9913 ± 0.002	0.9894 ± 0.0024
	**FONDUE_B1_BN**	0.9978 ± 7e-04	0.9968 ± 7e-04	0.9949 ± 0.0011	0.9927 ± 0.0016	0.9911 ± 0.002
	**FONDUE_B2_BN**	0.9963 ± 0.0013	0.9957 ± 0.0014	0.9952 ± 9e-04	0.9935 ± 0.0013	0.9919 ± 0.0018
	**FONDUE_B1_NOBN**	0.9982 ± 9e-04	0.9966 ± 9e-04	0.9948 ± 0.0014	0.993 ± 0.002	0.9906 ± 0.0032
	**FONDUE_B2_NOBN**	0.9979 ± 0.001	0.9963 ± 0.0015	0.9942 ± 0.0027	0.9917 ± 0.0038	0.9889 ± 0.0049
	**FONDUE_LT_X2**	0.9984 ± 5e-04	0.9974 ± 6e-04	0.9961 ± 9e-04	0.9946 ± 0.0013	0.9928 ± 0.002
**MSSSIM (higher is better): mean ± standard deviation**	**MCDnCNN**	0.999 ± 7e-04	0.9943 ± 0.0103	0.9851 ± 0.0301	0.979 ± 0.0384	0.9693 ± 0.0552
	**UNET_VINN**	0.9993 ± 3e-04	0.9975 ± 0.0031	0.9922 ± 0.0136	0.9844 ± 0.0279	0.9777 ± 0.0396
	**FONDUE_A**	0.9996 ± 1e-04	0.9992 ± 1e-04	0.9985 ± 4e-04	0.9975 ± 9e-04	0.9963 ± 0.0018
	**FONDUE_B**	0.9995 ± 1e-04	0.9989 ± 3e-04	0.998 ± 6e-04	0.9969 ± 0.0012	0.9953 ± 0.0022
	**FONDUE_LT**	0.9996 ± 1e-04	0.9992 ± 2e-04	0.9987 ± 3e-04	0.9979 ± 8e-04	0.9969 ± 0.0014
	**AONLM**	0.9997 ± 0	0.999 ± 2e-04	0.9978 ± 7e-04	0.9958 ± 0.002	0.9922 ± 0.0051
	**MRONLM**	0.9995 ± 1e-04	0.9987 ± 3e-04	0.9978 ± 5e-04	0.997 ± 7e-04	0.9961 ± 9e-04
	**ONLM**	0.9995 ± 1e-04	0.9987 ± 3e-04	0.9978 ± 5e-04	0.997 ± 7e-04	0.996 ± 9e-04
	**ODCT**	0.9997 ± 0	0.9992 ± 2e-04	0.9985 ± 4e-04	0.9977 ± 7e-04	0.9967 ± 9e-04
	**PRINLM**	0.9997 ± 0	0.9992 ± 2e-04	0.9985 ± 4e-04	0.9977 ± 7e-04	0.9966 ± 9e-04
	**FONDUE_A_BN**	0.9991 ± 3e-04	0.9988 ± 4e-04	0.9978 ± 7e-04	0.9957 ± 0.003	0.9956 ± 0.002
	**FONDUE_B_BN**	0.9992 ± 3e-04	0.9988 ± 4e-04	0.9978 ± 8e-04	0.9964 ± 0.0014	0.9957 ± 0.0014
	**FONDUE_B1_BN**	0.9992 ± 3e-04	0.9989 ± 3e-04	0.998 ± 6e-04	0.9966 ± 0.0013	0.9959 ± 0.0013
	**FONDUE_B2_BN**	0.9985 ± 8e-04	0.9983 ± 9e-04	0.9979 ± 6e-04	0.9969 ± 0.001	0.9961 ± 0.0012
	**FONDUE_B1_NOBN**	0.9993 ± 2e-04	0.9987 ± 4e-04	0.9977 ± 0.001	0.9966 ± 0.0015	0.9949 ± 0.0028
	**FONDUE_B2_NOBN**	0.9988 ± 0.0012	0.9978 ± 0.002	0.9962 ± 0.004	0.9944 ± 0.0053	0.9924 ± 0.0067
	**FONDUE_LT_X2**	0.9995 ± 1e-04	0.9991 ± 2e-04	0.9985 ± 5e-04	0.9977 ± 0.0011	0.9965 ± 0.0021

Table S.11: *Denoising performance comparison across noise levels, metrics, and methods for added stationary Rician noise on HCP test set.*

	Method	1%	3%	5%	7%	9%
LPIPS (lower is better): mean ± standard deviation	MCDnCNN	0.0012 ± 3e-04	0.0016 ± 4e-04	0.0035 ± 9e-04	0.0051 ± 0.001	0.0064 ± 0.0012
	UNET_VINN	6e-04 ± 2e-04	0.0013 ± 3e-04	0.0024 ± 5e-04	0.0036 ± 8e-04	0.0048 ± 0.001
	FONDUE_A	0.0012 ± 2e-04	0.0018 ± 4e-04	0.003 ± 7e-04	0.0044 ± 0.001	0.0058 ± 0.0014
	FONDUE_B	0.001 ± 3e-04	0.0018 ± 5e-04	0.0027 ± 7e-04	0.0034 ± 9e-04	0.0043 ± 0.001
	FONDUE_LT	6e-04 ± 1e-04	0.001 ± 2e-04	0.0017 ± 4e-04	0.0025 ± 5e-04	0.0033 ± 7e-04
	AONLM	5e-04 ± 1e-04	0.0022 ± 5e-04	0.0041 ± 9e-04	0.0064 ± 0.0013	0.0096 ± 0.002
	MRONLM	8e-04 ± 2e-04	0.0035 ± 9e-04	0.0055 ± 0.0012	0.0066 ± 0.0012	0.0076 ± 0.0013
	ONLM	7e-04 ± 2e-04	0.0032 ± 8e-04	0.0049 ± 0.0011	0.0063 ± 0.0012	0.0082 ± 0.0015
	ODCT	4e-04 ± 1e-04	0.0019 ± 5e-04	0.0038 ± 8e-04	0.0057 ± 0.0011	0.0076 ± 0.0014
	PRINLM	5e-04 ± 1e-04	0.002 ± 5e-04	0.0038 ± 8e-04	0.0054 ± 0.0011	0.0071 ± 0.0013
	FONDUE_A_BN	0.0023 ± 5e-04	0.0021 ± 5e-04	0.0024 ± 6e-04	0.0035 ± 8e-04	0.0052 ± 0.0012
	FONDUE_B_BN	0.0013 ± 4e-04	0.0015 ± 4e-04	0.0025 ± 5e-04	0.0041 ± 7e-04	0.0059 ± 0.0011
	FONDUE_B1_BN	0.0014 ± 5e-04	0.0018 ± 5e-04	0.0026 ± 6e-04	0.0035 ± 8e-04	0.0047 ± 0.001
	FONDUE_B2_BN	0.0025 ± 7e-04	0.0026 ± 7e-04	0.0032 ± 8e-04	0.0041 ± 0.001	0.0052 ± 0.0013
	FONDUE_B1_NOBN	0.0015 ± 6e-04	0.0026 ± 8e-04	0.0036 ± 0.001	0.0048 ± 0.0013	0.0063 ± 0.0015
	FONDUE_B2_NOBN	0.0016 ± 6e-04	0.0026 ± 7e-04	0.0036 ± 9e-04	0.0048 ± 0.0012	0.006 ± 0.0015
	FONDUE_LT_X2	0.0012 ± 2e-04	0.0016 ± 3e-04	0.0023 ± 5e-04	0.0031 ± 7e-04	0.0039 ± 9e-04
PSNR in dB (higher is better): mean ± standard deviation	MCDnCNN	50.5655 ± 0.4122	47.7378 ± 0.2965	45.11 ± 0.2886	43.3516 ± 0.2906	41.9364 ± 0.3672
	UNET_VINN	53.8034 ± 0.3327	48.8993 ± 0.3054	46.3045 ± 0.3006	44.3684 ± 0.345	42.8568 ± 0.4017
	FONDUE_A	52.2592 ± 0.2559	49.7204 ± 0.3597	46.8417 ± 0.7145	44.2458 ± 0.9911	41.6312 ± 1.3805
	FONDUE_B	52.5896 ± 0.8682	47.7464 ± 1.0442	44.2683 ± 1.3734	42.0796 ± 1.4034	40.4296 ± 1.3518
	FONDUE_LT	54.2167 ± 0.3442	49.8897 ± 0.3208	47.4679 ± 0.3355	45.7892 ± 0.3537	44.3941 ± 0.4377
	AONLM	51.572 ± 2.0233	48.046 ± 0.5342	45.7198 ± 0.3109	44.0335 ± 0.3405	42.6527 ± 0.2771
	MRONLM	53.5998 ± 0.57	48.2722 ± 0.3335	45.7529 ± 0.3384	44.3701 ± 0.2842	43.2309 ± 0.3173
	ONLM	54.2222 ± 0.3132	48.3085 ± 0.2905	45.7136 ± 0.2741	43.9633 ± 0.3091	42.5316 ± 0.2769
	ODCT	51.1428 ± 1.0178	44.7227 ± 0.9424	41.8816 ± 0.9492	40.3249 ± 1.4411	39.1316 ± 1.1647
	PRINLM	54.5752 ± 0.3547	49.0407 ± 0.2842	46.4161 ± 0.2935	44.6523 ± 0.3032	43.2648 ± 0.3111
	FONDUE_A_BN	50.1202 ± 0.3849	49.0241 ± 0.3637	47.1271 ± 0.3334	45.2679 ± 0.3051	43.9691 ± 0.3024
	FONDUE_B_BN	51.5685 ± 0.3271	48.6731 ± 0.3153	46.311 ± 0.3381	44.6712 ± 0.3323	43.441 ± 0.3148
	FONDUE_B1_BN	52.3529 ± 0.4027	49.1093 ± 0.322	46.8605 ± 0.3489	45.2621 ± 0.3528	44.058 ± 0.3413
	FONDUE_B2_BN	49.6893 ± 0.3709	48.5053 ± 0.3627	47.0203 ± 0.3671	45.7597 ± 0.3547	44.6613 ± 0.3418
	FONDUE_B1_NOBN	51.8718 ± 1.192	47.313 ± 1.2444	43.927 ± 1.5039	41.3737 ± 1.5534	39.0637 ± 1.4912
	FONDUE_B2_NOBN	52.1569 ± 0.8068	48.498 ± 0.766	45.3058 ± 1.1301	42.9671 ± 1.3687	40.9618 ± 1.4965
	FONDUE_LT_X2	52.0351 ± 0.3861	49.3107 ± 0.3574	47.2282 ± 0.3834	45.6181 ± 0.4227	44.2406 ± 0.5402
SSIM (higher is better): mean ± standard deviation	MCDnCNN	0.9983 ± 3e-04	0.9959 ± 6e-04	0.9925 ± 0.0011	0.9896 ± 0.0013	0.9869 ± 0.0018
	UNET_VINN	0.9987 ± 3e-04	0.9967 ± 4e-04	0.9948 ± 6e-04	0.993 ± 9e-04	0.9911 ± 0.0012
	FONDUE_A	0.9972 ± 3e-04	0.9974 ± 4e-04	0.996 ± 6e-04	0.9947 ± 9e-04	0.9932 ± 0.0013
	FONDUE_B	0.9985 ± 4e-04	0.9969 ± 5e-04	0.9952 ± 8e-04	0.9935 ± 0.0012	0.9919 ± 0.0015
	FONDUE_LT	0.9988 ± 2e-04	0.9973 ± 3e-04	0.9959 ± 5e-04	0.9946 ± 7e-04	0.9933 ± 9e-04
	AONLM	0.9988 ± 2e-04	0.9962 ± 5e-04	0.9933 ± 9e-04	0.9902 ± 0.0012	0.9866 ± 0.0016
	MRONLM	0.9989 ± 2e-04	0.9961 ± 6e-04	0.9937 ± 9e-04	0.9917 ± 0.001	0.9896 ± 0.0011
	ONLM	0.9989 ± 2e-04	0.996 ± 7e-04	0.9933 ± 9e-04	0.9907 ± 0.0011	0.9877 ± 0.0014
	ODCT	0.9988 ± 3e-04	0.9964 ± 5e-04	0.9939 ± 7e-04	0.9916 ± 9e-04	0.9893 ± 0.0012
	PRINLM	0.999 ± 2e-04	0.9968 ± 5e-04	0.9946 ± 7e-04	0.9925 ± 9e-04	0.9904 ± 0.0012
	FONDUE_A_BN	0.9977 ± 3e-04	0.9968 ± 4e-04	0.995 ± 6e-04	0.9926 ± 9e-04	0.9904 ± 0.0012
	FONDUE_B_BN	0.9982 ± 3e-04	0.9965 ± 4e-04	0.9941 ± 6e-04	0.9916 ± 9e-04	0.989 ± 0.0012
	FONDUE_B1_BN	0.9983 ± 4e-04	0.9968 ± 4e-04	0.9952 ± 5e-04	0.9934 ± 7e-04	0.9917 ± 8e-04
	FONDUE_B2_BN	0.9973 ± 5e-04	0.9967 ± 5e-04	0.9955 ± 6e-04	0.9943 ± 7e-04	0.993 ± 9e-04
	FONDUE_B1_NOBN	0.9982 ± 5e-04	0.9967 ± 6e-04	0.9951 ± 0.001	0.9933 ± 0.0014	0.9914 ± 0.0019
	FONDUE_B2_NOBN	0.9981 ± 5e-04	0.9967 ± 6e-04	0.9954 ± 8e-04	0.9939 ± 0.0012	0.9923 ± 0.0016
	FONDUE_LT_X2	0.9983 ± 3e-04	0.9973 ± 4e-04	0.9961 ± 5e-04	0.9949 ± 7e-04	0.9937 ± 9e-04
MSSSIM (higher is better): mean ± standard deviation	MCDnCNN	0.9995 ± 1e-04	0.999 ± 2e-04	0.9982 ± 3e-04	0.9975 ± 5e-04	0.9966 ± 8e-04
	UNET_VINN	0.9997 ± 0	0.9992 ± 1e-04	0.9986 ± 2e-04	0.998 ± 3e-04	0.9974 ± 5e-04
	FONDUE_A	0.9995 ± 1e-04	0.9993 ± 1e-04	0.9988 ± 3e-04	0.9981 ± 5e-04	0.9971 ± 0.001
	FONDUE_B	0.9996 ± 1e-04	0.9991 ± 2e-04	0.9983 ± 5e-04	0.9974 ± 9e-04	0.9964 ± 0.0012
	FONDUE_LT	0.9997 ± 0	0.9993 ± 1e-04	0.9989 ± 2e-04	0.9984 ± 3e-04	0.9978 ± 5e-04
	AONLM	0.9996 ± 1e-04	0.999 ± 2e-04	0.9982 ± 3e-04	0.9974 ± 5e-04	0.9963 ± 6e-04
	MRONLM	0.9996 ± 1e-04	0.9989 ± 2e-04	0.9982 ± 3e-04	0.9975 ± 4e-04	0.9968 ± 5e-04
	ONLM	0.9996 ± 0	0.9989 ± 2e-04	0.9982 ± 3e-04	0.9975 ± 4e-04	0.9967 ± 5e-04
	ODCT	0.9996 ± 1e-04	0.9989 ± 1e-04	0.998 ± 2e-04	0.997 ± 4e-04	0.9959 ± 7e-04
	PRINLM	0.9997 ± 0	0.9991 ± 1e-04	0.9985 ± 2e-04	0.9978 ± 4e-04	0.997 ± 5e-04
	FONDUE_A_BN	0.9994 ± 1e-04	0.9992 ± 1e-04	0.9988 ± 2e-04	0.9982 ± 3e-04	0.9976 ± 4e-04
	FONDUE_B_BN	0.9995 ± 1e-04	0.9992 ± 1e-04	0.9986 ± 2e-04	0.9979 ± 3e-04	0.9972 ± 4e-04
	FONDUE_B1_BN	0.9995 ± 1e-04	0.9992 ± 1e-04	0.9987 ± 2e-04	0.9981 ± 3e-04	0.9976 ± 4e-04
	FONDUE_B2_BN	0.9993 ± 1e-04	0.9991 ± 1e-04	0.9988 ± 2e-04	0.9983 ± 3e-04	0.9978 ± 4e-04
	FONDUE_B1_NOBN	0.9995 ± 1e-04	0.999 ± 3e-04	0.9982 ± 6e-04	0.9971 ± 0.0011	0.9956 ± 0.0016
	FONDUE_B2_NOBN	0.9995 ± 1e-04	0.9991 ± 2e-04	0.9984 ± 5e-04	0.9976 ± 8e-04	0.9965 ± 0.0013
	FONDUE_LT_X2	0.9995 ± 1e-04	0.9992 ± 1e-04	0.9988 ± 2e-04	0.9984 ± 3e-04	0.9979 ± 5e-04

Table S.12: *Denoising performance comparison across noise levels, metrics, and methods for added stationary Rician noise on IXI test set.*

	Method	1%	3%	5%	7%	9%
LPIPS (lower is better): mean ± standard deviation	MCDnCNN	8e-04 ± 5e-04	8e-04 ± 5e-04	0.0011 ± 4e-04	0.0014 ± 5e-04	0.0019 ± 6e-04
	UNET_VINN	3e-04 ± 2e-04	8e-04 ± 6e-04	0.0011 ± 6e-04	0.0015 ± 7e-04	0.0019 ± 7e-04
	FONDUE_A	4e-04 ± 5e-04	0.0011 ± 8e-04	0.0016 ± 9e-04	0.0022 ± 0.001	0.0028 ± 0.0011
	FONDUE_B	5e-04 ± 3e-04	0.001 ± 7e-04	0.0014 ± 8e-04	0.0018 ± 9e-04	0.0023 ± 0.001
	FONDUE_LT	4e-04 ± 4e-04	7e-04 ± 5e-04	0.001 ± 6e-04	0.0013 ± 7e-04	0.0016 ± 7e-04
	AONLM	5e-04 ± 5e-04	0.0012 ± 6e-04	0.0017 ± 7e-04	0.002 ± 7e-04	0.0023 ± 5e-04
	MRONLM	6e-04 ± 4e-04	0.0017 ± 7e-04	0.0024 ± 9e-04	0.0028 ± 0.001	0.003 ± 0.0011
	ONLM	5e-04 ± 3e-04	0.0015 ± 6e-04	0.002 ± 8e-04	0.0023 ± 8e-04	0.0026 ± 8e-04
	ODCT	2e-04 ± 0	0.001 ± 4e-04	0.002 ± 0.001	0.0029 ± 0.0012	0.0038 ± 0.0017
	PRINLM	2e-04 ± 1e-04	0.001 ± 4e-04	0.0018 ± 8e-04	0.0025 ± 9e-04	0.0031 ± 0.001
	FONDUE_A_BN	0.0019 ± 7e-04	0.0024 ± 7e-04	0.0027 ± 0.001	0.0025 ± 9e-04	0.0053 ± 0.0015
	FONDUE_B_BN	9e-04 ± 6e-04	9e-04 ± 6e-04	0.001 ± 5e-04	0.0012 ± 5e-04	0.0015 ± 5e-04
	FONDUE_B1_BN	8e-04 ± 6e-04	0.0011 ± 6e-04	0.0012 ± 7e-04	0.0014 ± 7e-04	0.0017 ± 7e-04
	FONDUE_B2_BN	0.0014 ± 8e-04	0.0015 ± 8e-04	0.0016 ± 8e-04	0.0019 ± 9e-04	0.0021 ± 9e-04
	FONDUE_B1_NOBN	5e-04 ± 4e-04	0.0012 ± 8e-04	0.0017 ± 0.001	0.0024 ± 0.0012	0.0032 ± 0.0013
	FONDUE_B2_NOBN	6e-04 ± 4e-04	0.0012 ± 8e-04	0.0017 ± 0.001	0.0023 ± 0.0012	0.0029 ± 0.0013
	FONDUE_LT_X2	7e-04 ± 6e-04	0.0011 ± 7e-04	0.0014 ± 8e-04	0.0017 ± 9e-04	0.0021 ± 8e-04
PSNR in dB (higher is better): mean ± standard deviation	MCDnCNN	52.0872 ± 2.0517	49.6621 ± 1.777	47.3216 ± 1.5419	45.7624 ± 1.6613	44.4517 ± 1.5291
	UNET_VINN	53.5123 ± 2.5886	49.3429 ± 1.9255	47.2724 ± 1.6829	45.6075 ± 1.7504	44.289 ± 2.0142
	FONDUE_A	54.5567 ± 2.1474	50.8091 ± 2.4487	48.7591 ± 2.1142	46.9832 ± 1.671	45.3999 ± 1.2663
	FONDUE_B	54.4173 ± 2.3952	50.1414 ± 2.2227	47.4716 ± 2.0612	45.5319 ± 1.7444	43.8763 ± 1.5629
	FONDUE_LT	54.818 ± 2.5463	51.0071 ± 2.1699	48.9851 ± 1.8994	47.5293 ± 1.6856	46.1977 ± 1.3176
	AONLM	53.0504 ± 3.8315	49.1945 ± 2.5007	47.0496 ± 2.1697	45.4011 ± 1.9534	44.2835 ± 1.7716
	MRONLM	52.5179 ± 4.0467	48.8451 ± 2.3995	46.7053 ± 2.5148	45.7027 ± 2.2555	44.8234 ± 2.3435
	ONLM	54.3884 ± 2.2726	49.4684 ± 1.9277	47.282 ± 1.9342	45.7911 ± 1.9007	44.5834 ± 2.0215
	ODCT	54.6318 ± 1.6032	48.9207 ± 2.5627	44.8688 ± 3.9996	43.4941 ± 4.1188	42.2191 ± 4.3564
	PRINLM	55.3782 ± 1.8084	50.1499 ± 2.3012	47.8647 ± 2.1482	46.3382 ± 2.0107	45.1522 ± 1.9313
	FONDUE_A_BN	44.4441 ± 2.0057	40.8083 ± 1.4923	40.379 ± 2.7502	41.2338 ± 2.7938	34.7579 ± 1.1677
	FONDUE_B_BN	52.2918 ± 2.4904	50.232 ± 2.1514	48.1905 ± 1.856	46.4732 ± 1.8135	45.272 ± 1.886
	FONDUE_B1_BN	52.5072 ± 2.5107	50.2725 ± 2.374	48.5467 ± 2.1931	46.9751 ± 2.2107	45.8138 ± 2.2735
	FONDUE_B2_BN	50.2837 ± 2.6931	49.5676 ± 2.5485	48.3124 ± 2.4834	46.7946 ± 2.6025	45.8969 ± 2.5782
	FONDUE_B1_NOBN	54.2877 ± 2.5138	50.0219 ± 2.4313	47.4403 ± 2.3201	45.0391 ± 1.9688	43.1027 ± 1.8283
	FONDUE_B2_NOBN	53.6003 ± 2.0786	50.1153 ± 2.4713	47.8018 ± 2.3976	46.1121 ± 2.3218	44.4162 ± 1.891
	FONDUE_LT_X2	52.7609 ± 2.6103	50.3407 ± 2.335	48.6765 ± 2.0149	47.402 ± 1.7666	46.1003 ± 1.3299
SSIM (higher is better): mean ± standard deviation	MCDnCNN	0.9983 ± 8e-04	0.9971 ± 8e-04	0.9955 ± 9e-04	0.9941 ± 0.0011	0.9927 ± 0.0012
	UNET_VINN	0.9988 ± 4e-04	0.9973 ± 0.001	0.9962 ± 0.0012	0.9953 ± 0.0014	0.9944 ± 0.0015
	FONDUE_A	0.9987 ± 8e-04	0.9975 ± 0.0013	0.9966 ± 0.0015	0.9957 ± 0.0016	0.9948 ± 0.0016
	FONDUE_B	0.9987 ± 6e-04	0.9974 ± 0.0012	0.9963 ± 0.0014	0.9953 ± 0.0015	0.9943 ± 0.0015
	FONDUE_LT	0.9988 ± 7e-04	0.9976 ± 0.001	0.9967 ± 0.0012	0.9958 ± 0.0013	0.995 ± 0.0013
	AONLM	0.9986 ± 7e-04	0.9969 ± 0.0011	0.9954 ± 0.0012	0.9939 ± 0.0013	0.9924 ± 0.0014
	MRONLM	0.9985 ± 6e-04	0.9965 ± 0.0012	0.9952 ± 0.0016	0.9942 ± 0.0018	0.9933 ± 0.0018
	ONLM	0.9986 ± 5e-04	0.9966 ± 0.0011	0.9953 ± 0.0014	0.994 ± 0.0015	0.9928 ± 0.0016
	ODCT	0.999 ± 2e-04	0.9971 ± 0.0011	0.9953 ± 0.0018	0.994 ± 0.0021	0.9925 ± 0.0027
	PRINLM	0.9989 ± 2e-04	0.9971 ± 0.0011	0.9957 ± 0.0016	0.9945 ± 0.0018	0.9934 ± 0.002
	FONDUE_A_BN	0.9969 ± 0.0012	0.9959 ± 0.0012	0.9948 ± 0.0012	0.9941 ± 0.0012	0.9904 ± 0.0017
	FONDUE_B_BN	0.9982 ± 9e-04	0.9974 ± 0.001	0.9962 ± 0.001	0.9949 ± 0.0011	0.9936 ± 0.0012
	FONDUE_B1_BN	0.9981 ± 0.001	0.9973 ± 0.0012	0.9964 ± 0.0013	0.9956 ± 0.0014	0.9947 ± 0.0015
	FONDUE_B2_BN	0.9972 ± 0.0015	0.9969 ± 0.0015	0.9963 ± 0.0015	0.9957 ± 0.0016	0.995 ± 0.0016
	FONDUE_B1_NOBN	0.9986 ± 7e-04	0.9972 ± 0.0014	0.9962 ± 0.0016	0.9952 ± 0.0018	0.9941 ± 0.0019
	FONDUE_B2_NOBN	0.9984 ± 8e-04	0.9971 ± 0.0014	0.9962 ± 0.0017	0.9954 ± 0.0019	0.9945 ± 0.0019
	FONDUE_LT_X2	0.9981 ± 0.0011	0.9973 ± 0.0014	0.9966 ± 0.0015	0.9959 ± 0.0015	0.9952 ± 0.0015
MSSSIM (higher is better): mean ± standard deviation	MCDnCNN	0.9996 ± 1e-04	0.9993 ± 1e-04	0.999 ± 2e-04	0.9986 ± 2e-04	0.9982 ± 4e-04
	UNET_VINN	0.9996 ± 1e-04	0.9993 ± 1e-04	0.999 ± 2e-04	0.9987 ± 3e-04	0.9983 ± 3e-04
	FONDUE_A	0.9997 ± 1e-04	0.9994 ± 2e-04	0.9992 ± 2e-04	0.9988 ± 3e-04	0.9984 ± 6e-04
	FONDUE_B	0.9997 ± 1e-04	0.9994 ± 2e-04	0.999 ± 2e-04	0.9986 ± 4e-04	0.998 ± 8e-04
	FONDUE_LT	0.9997 ± 1e-04	0.9994 ± 1e-04	0.9992 ± 2e-04	0.9989 ± 2e-04	0.9986 ± 3e-04
	AONLM	0.9996 ± 2e-04	0.9992 ± 2e-04	0.9989 ± 2e-04	0.9984 ± 3e-04	0.998 ± 3e-04
	MRONLM	0.9995 ± 2e-04	0.9992 ± 2e-04	0.9988 ± 3e-04	0.9985 ± 3e-04	0.9982 ± 3e-04
	ONLM	0.9996 ± 1e-04	0.9992 ± 2e-04	0.9989 ± 2e-04	0.9985 ± 3e-04	0.9982 ± 3e-04
	ODCT	0.9997 ± 0	0.9993 ± 2e-04	0.9985 ± 7e-04	0.9979 ± 9e-04	0.997 ± 0.0016
	PRINLM	0.9997 ± 0	0.9993 ± 2e-04	0.999 ± 2e-04	0.9986 ± 3e-04	0.9983 ± 3e-04
	FONDUE_A_BN	0.9986 ± 6e-04	0.9976 ± 9e-04	0.9969 ± 0.0022	0.9971 ± 0.0018	0.9923 ± 0.0036
	FONDUE_B_BN	0.9996 ± 1e-04	0.9994 ± 1e-04	0.9991 ± 1e-04	0.9988 ± 2e-04	0.9984 ± 2e-04
	FONDUE_B1_BN	0.9996 ± 1e-04	0.9994 ± 2e-04	0.9991 ± 2e-04	0.9988 ± 2e-04	0.9986 ± 2e-04
	FONDUE_B2_BN	0.9994 ± 2e-04	0.9993 ± 2e-04	0.9991 ± 2e-04	0.9988 ± 2e-04	0.9986 ± 3e-04
	FONDUE_B1_NOBN	0.9997 ± 1e-04	0.9993 ± 2e-04	0.999 ± 3e-04	0.9984 ± 5e-04	0.9976 ± 0.0012
	FONDUE_B2_NOBN	0.9996 ± 1e-04	0.9993 ± 2e-04	0.999 ± 3e-04	0.9986 ± 4e-04	0.9981 ± 7e-04
	FONDUE_LT_X2	0.9996 ± 1e-04	0.9994 ± 2e-04	0.9992 ± 2e-04	0.9989 ± 2e-04	0.9986 ± 3e-04

Table S.13: *Denoising performance comparison across noise levels, metrics, and methods for added stationary Rician noise on LA5c test set.*

	Method	1%	3%	5%	7%	9%
**LPIPS (lower is better): mean ± standard deviation**	**MCDnCNN**	0.001 ± 2e-04	0.001 ± 2e-04	0.0015 ± 4e-04	0.0022 ± 8e-04	0.0031 ± 0.0012
	**UNET_VINN**	3e-04 ± 1e-04	8e-04 ± 2e-04	0.0014 ± 4e-04	0.0236 ± 0.0252	0.0027 ± 9e-04
	**FONDUE_A**	3e-04 ± 1e-04	0.0011 ± 3e-04	0.0017 ± 4e-04	0.0024 ± 6e-04	0.0031 ± 8e-04
	**FONDUE_B**	3e-04 ± 1e-04	0.0011 ± 3e-04	0.0017 ± 4e-04	0.0022 ± 5e-04	0.0027 ± 7e-04
	**FONDUE_LT**	3e-04 ± 1e-04	8e-04 ± 2e-04	0.0012 ± 3e-04	0.0016 ± 4e-04	0.002 ± 5e-04
	**AONLM**	3e-04 ± 1e-04	0.0011 ± 3e-04	0.002 ± 5e-04	0.003 ± 9e-04	0.004 ± 0.0013
	**MRONLM**	3e-04 ± 1e-04	0.0012 ± 4e-04	0.0027 ± 9e-04	0.0042 ± 0.0011	0.0053 ± 0.0013
	**ONLM**	3e-04 ± 1e-04	0.0011 ± 4e-04	0.0023 ± 8e-04	0.0035 ± 0.0011	0.0046 ± 0.0014
	**ODCT**	2e-04 ± 1e-04	8e-04 ± 2e-04	0.002 ± 5e-04	0.0035 ± 7e-04	0.0048 ± 0.001
	**PRINLM**	1e-04 ± 0	7e-04 ± 3e-04	0.0018 ± 6e-04	0.003 ± 8e-04	0.0042 ± 0.0011
	**FONDUE_A_BN**	0.0019 ± 4e-04	0.0017 ± 4e-04	0.0019 ± 4e-04	0.0023 ± 5e-04	0.0031 ± 7e-04
	**FONDUE_B_BN**	0.001 ± 2e-04	0.0011 ± 2e-04	0.0014 ± 3e-04	0.0019 ± 5e-04	0.0024 ± 7e-04
	**FONDUE_B1_BN**	7e-04 ± 2e-04	0.0011 ± 3e-04	0.0017 ± 4e-04	0.002 ± 5e-04	0.0025 ± 6e-04
	**FONDUE_B2_BN**	0.0016 ± 3e-04	0.0017 ± 4e-04	0.0021 ± 4e-04	0.0025 ± 5e-04	0.003 ± 7e-04
	**FONDUE_B1_NOBN**	4e-04 ± 1e-04	0.0012 ± 3e-04	0.0019 ± 5e-04	0.0027 ± 7e-04	0.0036 ± 9e-04
	**FONDUE_B2_NOBN**	4e-04 ± 2e-04	0.0011 ± 3e-04	0.0019 ± 5e-04	0.0026 ± 6e-04	0.0032 ± 8e-04
	**FONDUE_LT_X2**	7e-04 ± 2e-04	0.0013 ± 3e-04	0.0017 ± 4e-04	0.0021 ± 5e-04	0.0026 ± 6e-04
**PSNR in dB (higher is better): mean ± standard deviation**	**MCDnCNN**	48.7108 ± 1.2155	46.3295 ± 0.9939	43.9388 ± 0.9036	42.1251 ± 0.8962	40.7888 ± 0.8915
	**UNET_VINN**	52.186 ± 1.0364	46.9919 ± 0.9428	44.5164 ± 0.9395	33.3199 ± 10.6676	41.6654 ± 0.9177
	**FONDUE_A**	52.3892 ± 1.248	47.4648 ± 1.2265	45.3315 ± 1.1135	43.8415 ± 1.0069	42.6922 ± 0.8669
	**FONDUE_B**	51.9751 ± 1.2147	46.9901 ± 1.1683	44.7764 ± 1.0673	43.3604 ± 0.8774	42.2185 ± 0.7318
	**FONDUE_LT**	51.9717 ± 1.3657	47.7981 ± 1.1702	45.7081 ± 1.0994	44.3099 ± 1.0486	43.2275 ± 0.98
	**AONLM**	50.4832 ± 2.1998	46.2931 ± 1.2978	43.901 ± 1.1421	42.3611 ± 1.0766	41.1299 ± 1.0046
	**MRONLM**	51.5691 ± 1.5425	46.9358 ± 0.9077	43.9988 ± 0.8187	42.1082 ± 0.964	40.8073 ± 1.1187
	**ONLM**	52.3452 ± 1.1199	47.1967 ± 0.7636	44.2544 ± 0.7571	42.3568 ± 0.8469	40.9083 ± 0.9626
	**ODCT**	50.8075 ± 1.7071	44.3749 ± 1.5696	40.8967 ± 2.1321	38.5144 ± 2.3051	37.8213 ± 2.0236
	**PRINLM**	53.5465 ± 0.732	47.3756 ± 0.8386	44.5468 ± 0.881	42.6792 ± 0.9267	41.3113 ± 0.954
	**FONDUE_A_BN**	46.4784 ± 1.2978	46.0302 ± 1.214	44.6951 ± 1.1552	43.3925 ± 1.1144	42.1912 ± 1.0911
	**FONDUE_B_BN**	48.5814 ± 1.3928	46.6355 ± 1.1673	44.7329 ± 1.0887	43.3194 ± 1.0701	42.2297 ± 1.0416
	**FONDUE_B1_BN**	49.7436 ± 1.3294	46.8074 ± 1.1293	44.7776 ± 1.122	43.4719 ± 1.1046	42.4039 ± 1.1017
	**FONDUE_B2_BN**	46.6762 ± 1.3802	45.8007 ± 1.281	44.5333 ± 1.1951	43.4724 ± 1.1709	42.4963 ± 1.1318
	**FONDUE_B1_NOBN**	51.9232 ± 1.2614	46.9064 ± 1.2051	44.6638 ± 1.0983	43.1336 ± 0.8906	41.8227 ± 0.7918
	**FONDUE_B2_NOBN**	51.593 ± 1.2455	46.9616 ± 1.2497	44.8373 ± 1.1917	43.465 ± 1.0376	42.3638 ± 0.8476
	**FONDUE_LT_X2**	49.4298 ± 1.5	46.7855 ± 1.3452	45.1764 ± 1.2282	44.0035 ± 1.123	43.0412 ± 1.0182
**SSIM (higher is better): mean ± standard deviation**	**MCDnCNN**	0.9989 ± 6e-04	0.9975 ± 7e-04	0.9954 ± 7e-04	0.9936 ± 0.001	0.9919 ± 0.0014
	**UNET_VINN**	0.9995 ± 5e-04	0.9978 ± 6e-04	0.9962 ± 7e-04	0.9639 ± 0.0339	0.9935 ± 0.0011
	**FONDUE_A**	0.999 ± 3e-04	0.9981 ± 6e-04	0.9969 ± 7e-04	0.9959 ± 8e-04	0.995 ± 9e-04
	**FONDUE_B**	0.9993 ± 3e-04	0.9978 ± 6e-04	0.9965 ± 6e-04	0.9954 ± 8e-04	0.9945 ± 8e-04
	**FONDUE_LT**	0.9992 ± 4e-04	0.9981 ± 6e-04	0.9971 ± 5e-04	0.9961 ± 7e-04	0.9952 ± 8e-04
	**AONLM**	0.9993 ± 6e-04	0.9976 ± 6e-04	0.996 ± 7e-04	0.994 ± 0.001	0.9921 ± 0.0012
	**MRONLM**	0.9993 ± 7e-04	0.9977 ± 7e-04	0.9959 ± 0.001	0.9942 ± 0.0011	0.9924 ± 0.0012
	**ONLM**	0.9996 ± 3e-04	0.998 ± 5e-04	0.9961 ± 8e-04	0.9942 ± 0.0011	0.9922 ± 0.0013
	**ODCT**	0.9991 ± 5e-04	0.998 ± 6e-04	0.9961 ± 7e-04	0.9939 ± 8e-04	0.992 ± 0.0011
	**PRINLM**	0.9997 ± 6e-04	0.9982 ± 5e-04	0.9966 ± 7e-04	0.9947 ± 8e-04	0.9932 ± 0.001
	**FONDUE_A_BN**	0.9975 ± 5e-04	0.9973 ± 6e-04	0.9962 ± 8e-04	0.995 ± 8e-04	0.9936 ± 9e-04
	**FONDUE_B_BN**	0.9984 ± 6e-04	0.9975 ± 5e-04	0.9963 ± 6e-04	0.9949 ± 8e-04	0.9936 ± 0.001
	**FONDUE_B1_BN**	0.9989 ± 5e-04	0.9979 ± 7e-04	0.9965 ± 5e-04	0.9953 ± 9e-04	0.9943 ± 9e-04
	**FONDUE_B2_BN**	0.9974 ± 7e-04	0.997 ± 7e-04	0.9963 ± 6e-04	0.9956 ± 7e-04	0.9947 ± 8e-04
	**FONDUE_B1_NOBN**	0.9992 ± 4e-04	0.9978 ± 7e-04	0.9966 ± 7e-04	0.9955 ± 9e-04	0.9945 ± 0.001
	**FONDUE_B2_NOBN**	0.9992 ± 5e-04	0.9977 ± 6e-04	0.9965 ± 7e-04	0.9956 ± 9e-04	0.9948 ± 0.0011
	**FONDUE_LT_X2**	0.9988 ± 6e-04	0.9978 ± 6e-04	0.9969 ± 7e-04	0.9961 ± 9e-04	0.9953 ± 7e-04
**MSSSIM (higher is better): mean ± standard deviation**	**MCDnCNN**	0.9998 ± 1e-04	0.9995 ± 1e-04	0.9992 ± 2e-04	0.9988 ± 2e-04	0.9984 ± 4e-04
	**UNET_VINN**	0.9998 ± 1e-04	0.9995 ± 1e-04	0.9992 ± 1e-04	0.9578 ± 0.0475	0.9986 ± 3e-04
	**FONDUE_A**	0.9998 ± 1e-04	0.9996 ± 1e-04	0.9994 ± 1e-04	0.9991 ± 2e-04	0.9989 ± 3e-04
	**FONDUE_B**	0.9998 ± 1e-04	0.9996 ± 1e-04	0.9993 ± 1e-04	0.999 ± 2e-04	0.9987 ± 4e-04
	**FONDUE_LT**	0.9997 ± 1e-04	0.9996 ± 1e-04	0.9994 ± 1e-04	0.9991 ± 1e-04	0.9989 ± 2e-04
	**AONLM**	0.9997 ± 1e-04	0.9995 ± 1e-04	0.9992 ± 2e-04	0.9987 ± 3e-04	0.9983 ± 4e-04
	**MRONLM**	0.9998 ± 1e-04	0.9995 ± 1e-04	0.9991 ± 2e-04	0.9987 ± 3e-04	0.9983 ± 4e-04
	**ONLM**	0.9998 ± 1e-04	0.9995 ± 1e-04	0.9991 ± 2e-04	0.9987 ± 3e-04	0.9983 ± 4e-04
	**ODCT**	0.9997 ± 1e-04	0.9994 ± 1e-04	0.9988 ± 2e-04	0.998 ± 4e-04	0.9975 ± 5e-04
	**PRINLM**	0.9998 ± 1e-04	0.9996 ± 1e-04	0.9993 ± 2e-04	0.9989 ± 2e-04	0.9985 ± 3e-04
	**FONDUE_A_BN**	0.9995 ± 1e-04	0.9994 ± 1e-04	0.9993 ± 1e-04	0.999 ± 2e-04	0.9987 ± 2e-04
	**FONDUE_B_BN**	0.9997 ± 1e-04	0.9995 ± 1e-04	0.9993 ± 1e-04	0.999 ± 2e-04	0.9987 ± 2e-04
	**FONDUE_B1_BN**	0.9997 ± 1e-04	0.9995 ± 1e-04	0.9993 ± 1e-04	0.999 ± 2e-04	0.9987 ± 2e-04
	**FONDUE_B2_BN**	0.9995 ± 1e-04	0.9995 ± 1e-04	0.9993 ± 1e-04	0.999 ± 1e-04	0.9988 ± 2e-04
	**FONDUE_B1_NOBN**	0.9998 ± 1e-04	0.9995 ± 1e-04	0.9993 ± 2e-04	0.999 ± 2e-04	0.9987 ± 5e-04
	**FONDUE_B2_NOBN**	0.9998 ± 1e-04	0.9995 ± 1e-04	0.9993 ± 1e-04	0.9991 ± 2e-04	0.9988 ± 3e-04
	**FONDUE_LT_X2**	0.9997 ± 1e-04	0.9996 ± 1e-04	0.9994 ± 1e-04	0.9992 ± 2e-04	0.999 ± 2e-04

Table S.14: *Denoising performance comparison across noise levels, metrics, and methods for added stationary Rician noise on MIRIAD test set.*

	Method	1%	3%	5%	7%	9%
LPIPS (lower is better): mean ± standard deviation	MCDnCNN	0.001 ± 2e-04	0.001 ± 1e-04	0.0015 ± 2e-04	0.0025 ± 3e-04	0.0034 ± 3e-04
	UNET_VINN	6e-04 ± 3e-04	0.001 ± 3e-04	0.0015 ± 3e-04	0.0022 ± 3e-04	0.0029 ± 4e-04
	FONDUE_A	4e-04 ± 2e-04	0.0013 ± 2e-04	0.002 ± 3e-04	0.0028 ± 5e-04	0.0036 ± 6e-04
	FONDUE_B	7e-04 ± 2e-04	0.0015 ± 5e-04	0.0021 ± 5e-04	0.0025 ± 5e-04	0.0029 ± 5e-04
	FONDUE_LT	6e-04 ± 2e-04	0.001 ± 2e-04	0.0013 ± 2e-04	0.0018 ± 3e-04	0.0023 ± 4e-04
	AONLM	4e-04 ± 1e-04	0.0014 ± 2e-04	0.0022 ± 3e-04	0.0029 ± 4e-04	0.0036 ± 4e-04
	MRONLM	5e-04 ± 1e-04	0.0019 ± 4e-04	0.0031 ± 5e-04	0.0041 ± 6e-04	0.0048 ± 6e-04
	ONLM	5e-04 ± 0	0.0017 ± 3e-04	0.0027 ± 5e-04	0.0035 ± 5e-04	0.0041 ± 5e-04
	ODCT	2e-04 ± 1e-04	9e-04 ± 1e-04	0.002 ± 2e-04	0.0032 ± 3e-04	0.0043 ± 4e-04
	PRINLM	1e-04 ± 0	0.001 ± 1e-04	0.0021 ± 2e-04	0.0032 ± 4e-04	0.0042 ± 4e-04
	FONDUE_A_BN	0.0023 ± 4e-04	0.002 ± 4e-04	0.002 ± 3e-04	0.0023 ± 3e-04	0.003 ± 4e-04
	FONDUE_B_BN	0.0012 ± 2e-04	0.0012 ± 2e-04	0.0014 ± 2e-04	0.0019 ± 2e-04	0.0025 ± 3e-04
	FONDUE_B1_BN	0.0011 ± 2e-04	0.0014 ± 2e-04	0.0017 ± 3e-04	0.0021 ± 3e-04	0.0026 ± 3e-04
	FONDUE_B2_BN	0.0022 ± 4e-04	0.0022 ± 4e-04	0.0025 ± 5e-04	0.0028 ± 5e-04	0.0032 ± 5e-04
	FONDUE_B1_NOBN	8e-04 ± 3e-04	0.0017 ± 5e-04	0.0024 ± 6e-04	0.0031 ± 7e-04	0.0038 ± 7e-04
	FONDUE_B2_NOBN	9e-04 ± 3e-04	0.0018 ± 5e-04	0.0025 ± 6e-04	0.0032 ± 7e-04	0.0038 ± 8e-04
	FONDUE_LT_X2	0.0013 ± 3e-04	0.0017 ± 4e-04	0.0021 ± 4e-04	0.0025 ± 5e-04	0.003 ± 5e-04
PSNR in dB (higher is better): mean ± standard deviation	MCDnCNN	49.2535 ± 0.299	46.4956 ± 0.274	43.9218 ± 0.2817	42.0485 ± 0.2957	40.7172 ± 0.3139
	UNET_VINN	51.3774 ± 0.8271	46.8442 ± 0.3749	44.4174 ± 0.3558	42.7692 ± 0.3667	41.5016 ± 0.3844
	FONDUE_A	52.0945 ± 0.5114	47.2437 ± 0.3704	45.0495 ± 0.4076	43.5381 ± 0.4524	42.3713 ± 0.4848
	FONDUE_B	51.1578 ± 0.6635	46.5414 ± 0.6286	44.4048 ± 0.5456	43.018 ± 0.5259	41.8998 ± 0.5432
	FONDUE_LT	51.3343 ± 0.5277	47.4013 ± 0.4026	45.2131 ± 0.4224	43.7286 ± 0.4613	42.6066 ± 0.4901
	AONLM	51.1314 ± 0.6292	46.2324 ± 0.4059	43.9775 ± 0.3057	42.3863 ± 0.3394	41.188 ± 0.3304
	MRONLM	51.1167 ± 0.3606	46.3343 ± 0.3607	43.9257 ± 0.4108	42.2743 ± 0.3636	41.1086 ± 0.3367
	ONLM	51.6984 ± 0.2918	46.4109 ± 0.3759	43.9218 ± 0.4004	42.2306 ± 0.3487	40.9034 ± 0.3527
	ODCT	49.7033 ± 1.4584	43.1709 ± 0.8137	40.1755 ± 1.1145	38.1951 ± 1.0796	37.8408 ± 1.2659
	PRINLM	53.0238 ± 0.3647	47.1376 ± 0.2534	44.4441 ± 0.2685	42.673 ± 0.2818	41.3768 ± 0.2966
	FONDUE_A_BN	46.5869 ± 0.4072	45.9271 ± 0.3975	44.5602 ± 0.3681	43.1799 ± 0.3642	41.9977 ± 0.3647
	FONDUE_B_BN	48.7414 ± 0.351	46.544 ± 0.3312	44.5181 ± 0.3239	43.0244 ± 0.3282	41.9049 ± 0.3282
	FONDUE_B1_BN	49.319 ± 0.4833	46.5786 ± 0.3759	44.6193 ± 0.361	43.2158 ± 0.3601	42.1241 ± 0.3583
	FONDUE_B2_BN	46.5151 ± 0.4657	45.5481 ± 0.4619	44.2462 ± 0.4501	43.2239 ± 0.4579	42.279 ± 0.4491
	FONDUE_B1_NOBN	50.8956 ± 0.7412	46.4139 ± 0.6676	44.3015 ± 0.5788	42.8813 ± 0.5598	41.7266 ± 0.5717
	FONDUE_B2_NOBN	50.5161 ± 0.7249	46.4341 ± 0.6782	44.408 ± 0.5978	43.0878 ± 0.5823	42.0526 ± 0.5769
	FONDUE_LT_X2	48.6909 ± 0.6941	46.3803 ± 0.549	44.7829 ± 0.522	43.5496 ± 0.5417	42.5462 ± 0.5655
SSIM (higher is better): mean ± standard deviation	MCDnCNN	0.9984 ± 5e-04	0.997 ± 4e-04	0.9949 ± 6e-04	0.9928 ± 7e-04	0.9911 ± 9e-04
	UNET_VINN	0.9989 ± 6e-04	0.9972 ± 4e-04	0.9953 ± 5e-04	0.9939 ± 5e-04	0.9925 ± 6e-04
	FONDUE_A	0.9991 ± 3e-04	0.9972 ± 5e-04	0.9957 ± 5e-04	0.9943 ± 7e-04	0.9931 ± 8e-04
	FONDUE_B	0.9988 ± 6e-04	0.9969 ± 4e-04	0.9954 ± 6e-04	0.9941 ± 5e-04	0.993 ± 6e-04
	FONDUE_LT	0.9989 ± 5e-04	0.9975 ± 5e-04	0.9959 ± 5e-04	0.9945 ± 5e-04	0.9935 ± 6e-04
	AONLM	0.999 ± 4e-04	0.9967 ± 4e-04	0.9947 ± 5e-04	0.9929 ± 7e-04	0.9911 ± 9e-04
	MRONLM	0.9991 ± 7e-04	0.9963 ± 4e-04	0.9944 ± 6e-04	0.9928 ± 8e-04	0.9914 ± 9e-04
	ONLM	0.9991 ± 3e-04	0.9965 ± 5e-04	0.9945 ± 6e-04	0.9928 ± 9e-04	0.9911 ± 8e-04
	ODCT	0.9989 ± 5e-04	0.9972 ± 4e-04	0.9949 ± 5e-04	0.9929 ± 7e-04	0.9913 ± 7e-04
	PRINLM	0.9993 ± 3e-04	0.9972 ± 4e-04	0.9952 ± 6e-04	0.9934 ± 6e-04	0.9917 ± 7e-04
	FONDUE_A_BN	0.9965 ± 7e-04	0.9962 ± 5e-04	0.9952 ± 7e-04	0.9938 ± 6e-04	0.9923 ± 8e-04
	FONDUE_B_BN	0.9976 ± 4e-04	0.9969 ± 5e-04	0.9954 ± 5e-04	0.9938 ± 7e-04	0.9923 ± 6e-04
	FONDUE_B1_BN	0.9979 ± 6e-04	0.9968 ± 5e-04	0.9954 ± 5e-04	0.994 ± 4e-04	0.9929 ± 6e-04
	FONDUE_B2_BN	0.9962 ± 5e-04	0.9959 ± 5e-04	0.995 ± 5e-04	0.994 ± 6e-04	0.9931 ± 8e-04
	FONDUE_B1_NOBN	0.9987 ± 6e-04	0.9966 ± 7e-04	0.995 ± 5e-04	0.9938 ± 7e-04	0.9928 ± 9e-04
	FONDUE_B2_NOBN	0.9985 ± 6e-04	0.9965 ± 6e-04	0.9949 ± 6e-04	0.9938 ± 7e-04	0.9928 ± 9e-04
	FONDUE_LT_X2	0.998 ± 7e-04	0.9966 ± 6e-04	0.9956 ± 6e-04	0.9943 ± 6e-04	0.9934 ± 8e-04
MSSSIM (higher is better): mean ± standard deviation	MCDnCNN	0.9997 ± 1e-04	0.9994 ± 1e-04	0.9991 ± 1e-04	0.9987 ± 1e-04	0.9983 ± 2e-04
	UNET_VINN	0.9997 ± 1e-04	0.9994 ± 1e-04	0.9991 ± 1e-04	0.9988 ± 1e-04	0.9985 ± 1e-04
	FONDUE_A	0.9997 ± 1e-04	0.9995 ± 1e-04	0.9992 ± 1e-04	0.9989 ± 1e-04	0.9986 ± 2e-04
	FONDUE_B	0.9997 ± 1e-04	0.9994 ± 1e-04	0.9991 ± 1e-04	0.9988 ± 1e-04	0.9985 ± 2e-04
	FONDUE_LT	0.9997 ± 1e-04	0.9995 ± 1e-04	0.9992 ± 1e-04	0.9989 ± 1e-04	0.9987 ± 2e-04
	AONLM	0.9997 ± 1e-04	0.9994 ± 1e-04	0.999 ± 1e-04	0.9986 ± 2e-04	0.9982 ± 2e-04
	MRONLM	0.9997 ± 1e-04	0.9993 ± 2e-04	0.9989 ± 2e-04	0.9986 ± 2e-04	0.9982 ± 2e-04
	ONLM	0.9997 ± 1e-04	0.9993 ± 1e-04	0.999 ± 2e-04	0.9986 ± 2e-04	0.9982 ± 2e-04
	ODCT	0.9997 ± 1e-04	0.9993 ± 1e-04	0.9986 ± 2e-04	0.9979 ± 3e-04	0.9975 ± 4e-04
	PRINLM	0.9998 ± 1e-04	0.9995 ± 1e-04	0.9991 ± 1e-04	0.9987 ± 2e-04	0.9983 ± 2e-04
	FONDUE_A_BN	0.9993 ± 1e-04	0.9993 ± 1e-04	0.9991 ± 1e-04	0.9988 ± 1e-04	0.9985 ± 1e-04
	FONDUE_B_BN	0.9995 ± 1e-04	0.9994 ± 1e-04	0.9991 ± 1e-04	0.9988 ± 1e-04	0.9985 ± 2e-04
	FONDUE_B1_BN	0.9996 ± 1e-04	0.9994 ± 1e-04	0.9991 ± 1e-04	0.9988 ± 1e-04	0.9986 ± 2e-04
	FONDUE_B2_BN	0.9993 ± 1e-04	0.9992 ± 1e-04	0.999 ± 1e-04	0.9988 ± 1e-04	0.9986 ± 2e-04
	FONDUE_B1_NOBN	0.9997 ± 1e-04	0.9994 ± 1e-04	0.999 ± 1e-04	0.9987 ± 2e-04	0.9984 ± 2e-04
	FONDUE_B2_NOBN	0.9997 ± 1e-04	0.9993 ± 1e-04	0.9991 ± 1e-04	0.9988 ± 2e-04	0.9985 ± 2e-04
	FONDUE_LT_X2	0.9996 ± 2e-04	0.9994 ± 1e-04	0.9991 ± 1e-04	0.9989 ± 1e-04	0.9987 ± 2e-04

Table S.15: *Comparison between denoising single repetition images with the different denoising methods vs averaging multiple acquisitions of the same image (rows 1 to 20) on CUSTOM_0.5_20rep dataset. In bold are shown the best methods for each metric for each type of technique, and underlined are the second-best methods. Note: Reference image was the average of 20 repetitions with final filtering using AONLM. Results for subject 1 of the validation set.*

	Image (number of repetitions averaged)	LPIPS	PSNR (dB)	SSIM	MSSSIM
AVERAGING	1_rep	0.0371	23.802	0.9484	0.9747
	2_rep	0.0241	26.319	0.9632	0.9854
	3_rep	0.0183	27.818	0.9702	0.9894
	4_rep	0.0148	29.397	0.9748	0.9922
	5_rep	0.0124	30.091	0.9779	0.9934
	6_rep	0.0107	31.345	0.9803	0.9946
	7_rep	0.0094	32.293	0.9823	0.9955
	8_rep	0.0082	32.394	0.9841	0.9958
	9_rep	0.0072	32.602	0.9856	0.9960
	10_rep	0.0064	34.297	0.9867	0.9969
	11_rep	0.0058	34.754	0.9878	0.9972
	12_rep	0.0052	35.900	0.9887	0.9976
	13_rep	0.0048	36.693	0.9895	0.9978
	14_rep	0.0043	35.744	0.9903	0.9978
	15_rep	0.0039	35.399	0.9912	0.9978
	16_rep	0.0036	37.346	0.9916	0.9982
	17_rep	0.0033	37.457	0.9924	0.9983
	18_rep	0.0031	36.558	0.9929	0.9982
	19_rep	<u>0.0029</u>	37.961	0.9934	0.9985
	20_rep	**0.0027**	38.009	0.9937	0.9986
NON-DL	AONLM	**0.0055 ± 0.00107**	**32.2 ± 1.19**	**0.981 ± 0.00239**	**0.994 ± 0.000876**
	MRONLM	<u>0.0125 ± 0.00129</u>	<u>26.4 ± 0.602</u>	<u>0.971 ± 0.00204</u>	<u>0.986 ± 0.00155</u>
	ONLM	0.0135 ± 0.00124	26.2 ± 0.535	0.97 ± 0.00199	0.985 ± 0.00152
	ODCT	0.0276 ± 0.00125	23.5 ± 0.397	0.953 ± 0.00186	0.973 ± 0.00227
DL STAND ALONE	UNET-VINN	0.00316 ± 0.000526	<u>36.8 ± 1.43</u>	0.985 ± 0.00178	<u>0.996 ± 0.00058</u>
	MCDNCNN	0.00819 ± 0.00104	30.1 ± 0.643	0.977 ± 0.00212	0.992 ± 0.00101
	FONDUE_A_BN	**0.00285 ± 0.000421**	34.6 ± 0.916	**0.987 ± 0.00134**	**0.996 ± 0.000427**
	FONDUE_A_NOBN	0.0039 ± 0.00112	36 ± 1.31	0.984 ± 0.00265	0.995 ± 0.000938
	FONDUE_B_BN	0.0043 ± 0.000665	33.8 ± 0.831	0.984 ± 0.0015	0.995 ± 0.000556
	FONDUE_B_NOBN	<u>0.0029 ± 0.000389</u>	**38.6 ± 2.08**	<u>0.986 ± 0.00128</u>	0.996 ± 0.000627
	FONDUE_LT	0.00714 ± 0.00137	30.8 ± 1.01	0.979 ± 0.00255	0.993 ± 0.00115
DL SECOND STAGE	FONDUE_LT_X2	0.00364 ± 0.000826	33.5 ± 1.6	0.986 ± 0.00231	0.995 ± 0.00109
	FONDUE_B1_BN	0.00314 ± 0.000577	33.5 ± 1.06	0.986 ± 0.0017	0.995 ± 0.000607
	FONDUE_B1_NOBN	<u>0.00208 ± 0.000309</u>	<u>39.2 ± 2.2</u>	0.989 ± 0.00129	0.997 ± 0.000636
	FONDUE_B2_BN	0.00211 ± 0.000286	37.2 ± 1.48	<u>0.99 ± 0.00153</u>	**0.997 ± 0.000412**
	FONDUE_B2_NOBN	**0.00188 ± 0.00029**	**39.5 ± 2.18**	**0.99 ± 0.00132**	<u>0.997 ± 0.00063</u>
